# Supplementary material for: What makes a sustainability tool valuable, practical and useful in real-world healthcare practice? A mixed-methods study on the development of the Long Term Success Tool in Northwest London
Source: BMJ Open. 2017 Sep 24;7(9):e014417. doi: 10.1136/bmjopen-2016-014417 (PMC5623390; doi:10.1136/bmjopen-2016-014417)
Supplement: Supplementary file 1 [file bmjopen-2016-014417supp001.pdf]

Appendix A

Authors	Year	Model/Concept	Purpose	Type	Sustainability Constructs Identified	Scoring
Goodman	1993	Level of institutionalization (LOIN) scales	Research instrument and diagnostic tool	Scale	8 factor model- 15 three part items. Production routine, Production niche saturation, Maintenance routine, Maintenance niche saturation, supportive routine, Supportive niche saturation, Managerial routine, Managerial niche saturation	Likert scale
Kotter	1995	Anchoring Change	Raise awareness of the common errors made during change initiatives	Model	Establishing a sense of urgency, Formation of a powerful guiding coalition, Creating a vision, Communicating the vision, Empowering others to act on the vision, Planning for and creating short term wins, Consolidating improvements and producing more change, Institutionalising new approaches.	none
Dale et al.	1997	The TQM sustainability audit tool (TQMSAT)	Looks for a specific set of predetermined negative factors; that have been identified as detrimental to sustaining TQM	Tool	Internal/external environment, Management Style, Policies, Organisation structure, Process of change. 23 sub themes including: communication, human resource, improvement infrastructure, education and training	Rate each of the issues on a scale of 1 to 6
Shediac-Rizkallah& Bone	1998	Conceptual framework for planning for sustainability of community based health programs	An organizing framework for conceptualizing and measuring sustainability and tentative guidelines to facilitate sustainability in community programs	Conceptual Framework	(1) Project design and implementation factors, Project negotiation process. Project effectiveness. Project duration. Project financing Project type, Training<br>(2) Factors within the organizational setting: Institutional strength Integration with existing programs/services<br>Program champion/leadership<br>(3) Factors in the broader community environment:<br>Socioeconomic and political considerations<br>Community participation.	none
Glasgow et al.	1999	RE-AIM	Provides a framework for determining what programs are worth sustained investment and for identifying those that work in real-world environments	Framework	Reach- patient and staff participation, Efficacy- positive and negative effects of interventions, Adoption-proportion and representation of settings, Implementation-extent to which intervention is delivered as intended and Maintenance-institutionalisation, routine, culture and norms.	0 to 1 (or 0% to 100%) scale.

Appendix A

Johnson et al	2004	A sustainability Planning Model	Addresses factors known to inhibit efforts to sustain an innovation	Model	Sustainability Capacity, Administrative structures and linkages, Champion roles and leadership actions, resources, policies and procedures, Expertise, Sustainable innovation attributes, Alignment, positive relationships, implementation quality and effectiveness, sustainable actions	none
Sirkin	2005	DICE tool	To rate and track transformation initiative progress	Tool	Duration, Integrity of performance, Senior management commitment, Local-level commitment, effort	Likert scale (1-4) Low scores being better.
Edwards	2007	Catholic Healthcare partners HF-GAP Sustainability Assessment (AHRQ)	To trigger planning for sustainability early in a project's design	Framework and checklist	Five components for developing sustainability: identity goals, infrastructure, incentives, incremental opportunities for participation, and integration	Rate your organisation: -1: Problem, 0: Neither positive or negative, 1: Strength
Gruen	2008	Health-programme sustainability	To provide comprehensive sustainability planning	Model	Context and resource availability, encompassing health concerns, interventions, and drivers, emphasising dynamic interactions between components	none
Feldstein & Glasgow	2008	A practical robust implementation and sustainability model (PRISM)	To enhance implementation and sustainability	Model	Program, external environment, Implementation and Sustainability Infrastructure, Recipients with 39 sub-elements to assess	none
Bowman	2008	QUERI Implementation Model	Proposes a number of recommendations regarding the design of a sustainability analysis	Framework and model	1) intervention fit, 2) intervention fidelity, 3) intervention dose, and<br>4) level of the intervention target	none
Johnson et al	2009	A sustainability toolkit for prevention using getting to outcomes	To provide a process to organize, implement, and evaluate an effective plan for sustaining prevention infrastructure and interventions	Toolkit	Capacity, Infrastructure, Intervention, Routinization, Benefits to diverse populations	none

Appendix A

Damschroder	2009	Consolidated Framework For Implementation Research	To guide formative evaluations and build the implementation knowledge base across multiple studies and settings	Framework	8 constructs related to the intervention (e.g., evidence strength and quality), 4 constructs to outer setting (e.g., patient needs and resources), 12 constructs to inner setting (e.g., culture, leadership engagement), 5 constructs related to individual characteristics, and 8 constructs related to process (e.g., plan, evaluate, and reflect)	none
Maher	2010	NHS sustainability Model	To predict the likelihood of sustainability and guide teams to things they could do to increase the chances that the change for improvement will be sustained	Model	10 factors: Staff involvement and training, Staff attitudes towards sustaining the change, Senior leadership engagement, Clinical leadership, Fit with the organisation's strategic aims and culture, Infrastructure for sustainability, Benefits beyond helping patient, Credibility of the benefits, Adaptability of improved process, Effectiveness of the system to monitor progress	Weighted scoring system- Max 30 points
Douglas	2012	The Program Sustainability Assessment Tool	To assess and plan for sustainability risks and develop an action plans	Tool	Political support, funding stability, partnerships organisational capacity, program evaluation, program adaptation, communications, strategic planning	7 point Likert scale (To little or no extent- To a very great extent)
Kaplan	2012	The Model for Understanding Success in Quality (MUSIQ)	To examine the role of context in QI and how context influences QI success	Model	Factors effecting context: External Environment, Organisation, QI support and Capacity, Microsystem, QI Team, Miscellaneous	none
