# Supplementary material for: What makes a sustainability tool valuable, practical and useful in real-world healthcare practice? A mixed-methods study on the development of the Long Term Success Tool in Northwest London
Source: BMJ Open. 2017 Sep 24;7(9):e014417. doi: 10.1136/bmjopen-2016-014417 (PMC5623390; doi:10.1136/bmjopen-2016-014417)
Supplement: Author's manuscript [file bmjopen-2016-014417.draft_revisions.pdf]

BMJ Open is committed to open peer review. As part of this commitment we make the peer review history of every article we publish publicly available.

When an article is published we post the peer reviewers' comments and the authors' responses online. We also post the versions of the paper that were used during peer review. These are the versions that the peer review comments apply to.

The versions of the paper that follow are the versions that were submitted during the peer review process. They are not the versions of record or the final published versions. They should not be cited or distributed as the published version of this manuscript.

BMJ Open is an open access journal and the full, final, typeset and author-corrected version of record of the manuscript is available on our site with no access controls, subscription charges or pay-per-view fees (http://bmjopen.bmj.com).

If you have any questions on BMJ Open's open peer review process please email info.bmjopen@bmj.com

BMJ Open

The Long Term Success Tool: a practical approach to sustaining improvements in healthcare

Journal:	*BMJ Open*
Manuscript ID	bmjopen-2016-014417
Article Type:	Research
Date Submitted by the Author:	23-Sep-2016
Complete List of Authors:	Lennox, Laura; CLAHRC for Northwest London, Medicine; Imperial College London, Medicine<br>Doyle, Cathal; NIHR CLAHRC for Northwest London<br>Reed, Julie; NIHR, CLAHRC for NW London<br>Bell, Derek; Imperial College, Acute Medicine
**Primary Subject Heading**:	Health services research
Secondary Subject Heading:	Research methods
Keywords:	Quality Improvement, Tool, Sustainability

SCHOLARONE™
Manuscripts

For peer review only

Title of the article:

The Long Term Success Tool: a practical approach to sustaining improvements in healthcare

Corresponding author:

Laura Lennox, 369 Fulham Road, Chelsea and Westminster Hospital, London, SW10 9NH. l.lennox@imperial.ac.uk, 0203 315 3392. Imperial College London.

Co-authors:

Cathal Doyle, NIHR CLAHRC Northwest London, United Kingdom
Julie Reed, NIHR CLAHRC Northwest London, United Kingdom
Derek Bell, NIHR CLAHRC Northwest London, United Kingdom

Word count: 3452

For peer review only

Abstract

Objectives: Although improvement initiatives show benefits to patient care, they often fail to sustain. Models and frameworks exist to address this challenge, but issues with design, clarity and usability have been barriers to past use in healthcare settings. This work aimed to collaborate with stakeholders to develop an evidence-based sustainability tool relevant to people in healthcare settings, and practical for use in improvement initiatives.

Design: A literature review of sustainability approaches was conducted, providing evidence of factors influencing sustainability. Facilitated group discussions and interviews gathered perspectives on relevance of identified themes and potential design of a sustainability tool. Piloting collected views to adapt the tool to ensure practicality for routine use.

Setting: Research was conducted within the National Institute for Health Research Collaboration for Leadership in Applied Health Research and Care for Northwest London (CLAHRC NWL).

Participants: CLAHRC NWL improvement initiative teams and staff.

Results: The Long Term Success Tool supports those implementing improvements to reflect on 12 sustainability factors: Commitment to the improvement, Involvement, Skills and capabilities, Leadership, Team functioning, Resources in place, Evidence of benefits, Progress monitored for feedback and learning, Robust and adaptable processes, Alignment with organisational culture and priorities, Support for improvement, Alignment with external political and financial environment. Iterative work with stakeholders identified improvements in articulation of the evidence base, relevance to stakeholders and practical application. The Tool is designed to provide a platform for improvement teams to identify sustainability risks and highlight actions influencing sustainability.

Conclusion: Developing a tool for sustainability that is comprehensive and practical has been a significant challenge throughout this work, but working collaboratively with stakeholders provided insight into how this balance can be achieved. Further research is required to study the use and effectiveness of the tool in practice and assess engagement with the method over time.

For peer review only

Article summary

Strengths and limitations of this study

- The LTST provides a mechanism for improvement teams to identify risks to sustainability and importantly can create an environment for team members to highlight specific actions to be taken and comment on ways to influence sustainability over time.
- The LTST builds on established literature and aligns well with other sustainability models but is distinguished from other approaches by its practical design and ability to draw on team suggestions for action planning.
- Developing a tool for sustainability that is both comprehensive and practical has been a key challenge throughout this work.
- While attempts have been made to respond to user preferences and create a simple tool, it is important to note that some challenges could not be avoided and require further research.
- Further research is required to study the use and effectiveness of the tool in practice and assess engagement with the method over time.

For peer review only

Introduction and Objectives

Significant financial and human resources are invested in initiatives to improve the quality of healthcare and deliver better patient outcomes. While many initiatives show patient benefits or improvements in care processes or clinical outcomes initially (e.g. in the period when resource is available to introduce new practice), these often fail to sustain in the longer term.(1–5) As a result there is growing research interest in this area, with studies showing wide variation in the sustainability of initiatives. Self-reporting measures have reported that up to 60% of programs sustain (at least in part), while studies using more objective measures of sustainability (such as independent observation) report lower rates of sustainability from 6.7% - 45%.(3,6)

This area of research is further complicated by several definitions of sustainability in the literature and little consensus on what constitutes 'achieving sustainability'.(1,7) Despite these issues, three domains of sustainability have been consistently used within the literature; *continuation of initiative activities* (maintenance of the intervention or practices that were introduced), *continuation of the health benefits* which resulted from the initiative (health outcomes remain stable or get better) and *capacity built in the workforce* (the skills gained by being involved in the initiative that can support on-going high quality care or the attainment of skills which enable the workforce to continually improve).(1)

Given the complexity and dynamic nature of health care and health care delivery, we believe that all three domains are necessary to define and assess sustainability. For these reasons we have chosen to define sustainability as: a dynamic process where staff and others involved have the capacity and capability to monitor and modify activities and interventions in relation to the health benefits they wish to achieve and in response to threats and opportunities that emerge over time. As sustainability is being seen as a process and not an end point, this definition does not include a specific timeframe for sustainability. Timeframes should be defined by initiative teams and stakeholders and be based on the goals of the improvement initiative with respect to the intervention, desired outcomes, disease area and setting.

Navigating the relationship between achieving initial 'successful' implementation and achieving long term sustainability is a challenge.(1,8–10) It has been noted that over 60% of implementation frameworks include sustainability stages.(11) Factors contributing to sustainability of improvement initiatives often relate to how the improvement initiative is planned and conducted from the outset, suggesting an interdependent relationship between factors that influence initial success and those that influence long term success.(1,8,9) Although the evidence shows an overlap in factors

For peer review only

influencing both implementation success and sustainability, there is lack of clarity on what conditions may result in initial success but may or may not result in the sustainability of improvements. For example, an initiative may achieve initial success by providing extra resource or putting pressure on the workforce, but once the resource or pressure are removed the benefits achieved are not sustained.

Addressing Sustainability in Practice:

Multiple approaches, including models, frameworks and tools have been designed to highlight sustainability factors.(10,12,13) Research on the application of quality improvement methods in healthcare has shown that using methods well in practice is a consistent challenge for improvement teams, highlighting the need for methods to be practical in real-world healthcare settings. (8,14–19)

The application of one such method, the NHS Institute for Innovation and Improvement Sustainability Model (SM), was previously described.(8,20) Results demonstrated that while the SM raised awareness of determinants of sustainability and was perceived as valuable, teams found it difficult to understand and to apply routinely.(8,20) In particular, concerns were raised about the clarity the language used within the model, the user-friendliness of design, the length of time taken to complete the questions and suitability for continuous use in healthcare settings.(8)

The purpose of this study was to develop a tool for improvement teams to plan, and reflect on, factors important to sustainability at all stages of an initiative, to prompt discussion and action to enhance chances of achieving sustainability. For the tool to be valuable to improvement teams we aimed for it to be:

1. Evidence-based – to ensure a comprehensive review of sustainability factors
2. Relevant to stakeholders in healthcare settings - Useful, meaningful and appropriate for stakeholder settings and knowledge base
2. Practical to use in improvement initiatives - Realistic time to complete and easy to use with regular review throughout initiatives to ensure changing priorities could be addressed

This article describes work undertaken with healthcare professionals, patients, and researchers to develop a tool to meet the needs of people in improvement initiatives.

Design

Setting:

For peer review only

Research was conducted within the National Institute for Health Research (NIHR) Collaboration for Leadership in Applied Health Research and Care for Northwest London (CLAHRC NWL).(21) CLAHRC NWL improvement projects cover a range of health problems and disease areas that include primary care, secondary care, and community settings that are delivered over 18-24 months with the aim of sustaining improvements beyond this period.

Scoping Literature Review:

A scoping literature review was undertaken to examine the extent, range and nature of research activity related to sustainability approaches.(22) A systematic review was not deemed necessary to identify factors for sustainability as a number of studies and reviews had previously been published.(3,6,23) These reviews were used as a starting point to identify relevant authors and publications including snowballing of relevant journal articles, reference lists and the PubMed options of 'similar article' and 'cited by-' articles. This technique allowed us to identify relevant sustainability approaches from the literature. All sustainability constructs (questions, themes, criteria) identified in the approaches were extracted for thematic analysis and aggregate themes were developed.

Group discussions:

Three facilitated group discussions were held with CLAHRC NWL team members to understand the perceived relevance of the literature review results against CLAHRC NWL team expertise and experience. Discussions were held during a routine weekly CLAHRC NWL meeting between core staff. Participants represented a 'natural group' of professionals with a wide range of expertise and experience in supporting improvement projects (Table 1)(24). The themes from the literature were provided on paper hand-outs to the attendees and an open discussion took place to determine the relevance, resonance, and clarity of the themes presented. Observation notes were taken during group discussions to inform iterations of language and representation of themes.

Table 1: Group Discussion Attendees by Role

Attendees	Discussion 1	Discussion 2	Discussion 3
Doctor	2	1	-
Information/Data Analyst	2	4	2
Nurse	1	-	-
Project Manager	1	1	1
Project Support Officer	1	2	1
QI Facilitator	2	4	4
QI programme Manager	1	2	1

For peer review only

Researcher	2	2	2
Total	12	16	11

Stakeholder engagement event:

Consolidated sustainability themes were presented to stakeholders at a CLAHRC NWL Collaborative Learning event in April 2014 to check the relevance and language against stakeholder views. In facilitated group discussions, participants (n=74 including past and current improvement project members, academic and industry partners, patients, and carers) provided their views on the clarity, relevance, and resonance of these factors. Designated note takers were assigned to take observation notes capturing key learning and suggestions.

Interviews:

Interviews (n=12) aimed to collect in-depth information on practicality of tool design. A purposive sampling strategy was used to recruit interviewees. Participants were selected based on their role within the improvement project, their level of knowledge of the project, and their experience with the SM. This approach aimed to maximize the diversity of perspectives gained from the interviews.(25) All participants were CLAHRC NWL stakeholders who were either 1) involved in a past project and had experience of using the SM 2) would be involved in a future project and had no experience of using the SM 3) CLAHRC NWL core team member. Table 2 highlights perspectives gained and interviewee roles. All interviews were carried out face-to-face in a workplace setting by one author (LL). Each interview lasted between 30-60 minutes. A semi-structured interview guide was used for all interviews. Interview questions explored the design of questions and statements used to draw attention to factors for sustainability as well as views on collating and presenting data to facilitate discussion and action. Interviews were audio recorded and uploaded onto qualitative software Nvivo (version 9). Audio recordings were coded directly on Nvivo using thematic content analysis by one author.(26)

Table 2: Interviewee perspective and role

Interview ID	Role	Perspective
I1	Doctor	Prospective team member
I2	Pharmacist	Prospective project member
I3	Doctor	Prospective project member
I4	Doctor	Prospective project member
I5	Project manager	Past project member
I6	Doctor	Past project member
I7	Pharmacist/Project manager	Past project member

For peer review only

I8	Patient representative	Past project member
I9	Nurse specialist	Past project member
I10	Project manager	Past project member
I11	QI facilitator	Core team
I12	QI facilitator	Core team

Small scale trialling:

A group of individuals involved in studying QI approaches and leading QI projects as part of a CLAHRC NWL fellowship programme (n=11) were asked to trial the draft version of the tool. This aimed to understand the practical application of the tool including the approximate amount of time to complete by a wide range of people with diverse experience and expertise in improvement initiatives. Critical feedback and suggestions for tool development were recorded as observation notes as the participants used the tool in the session.

Piloting:

The resulting tool was piloted in July 2014 with 83 participants. Piloting aimed to provide an opportunity for further comments and suggestions on practicality of the tool in healthcare settings, and to measure if the tool could be completed within an acceptable time (approximately 10-15 minutes). Participants were given 15 minutes to complete the tool and a 20 minute facilitated group discussion followed. Facilitated discussions took place and note takers recorded key observations and feedback.

Results

Tool Development

Each step in the methods allowed for iterative development of concepts, content, and design of the tool to achieve research aims of an evidenced based, relevant and practical tool for sustainability. Key iterations of the themes, factors, and tool design elements are summarized in Figure 1.

Figure 1: Factor and Tool Iteration

The literature review retrieved 16 sustainability approaches (frameworks, models, tools). Results showed reasonable consensus in the literature on what factors influence sustainability. Thematic analysis of the constructs with these approaches identified 25 themes impacting sustainability (fig.1). Appendix A summarises the approaches found and the key sustainability criteria extracted.

For peer review only

The internal CLAHRC NWL group discussions identified themes that could be consolidated to reduce the number of constructs presented to stakeholders and suggested changes to the language of the themes to be relevant to improvement settings. These discussions resulted in a consolidated list of 12 factors impacting sustainability.

The stakeholder engagement event demonstrated most factors from the literature resonated with stakeholders and were recognised as relevant to healthcare settings, but in some cases the literature findings needed to be adapted to align with stakeholder expertise and understanding. For example, 'Fit with Current Practice' was found to be problematic for participants. Although this factor was meant to convey the importance of interventions being aligned with current practice, many stakeholders mentioned that often improvements must be different from the current ways of working so trying to fit in with 'current practices' would not be desirable or possible. From this discussion the need to amend this factor to aid shared understanding was identified and the factor was changed to 'Robust and Adaptable Processes' highlighting the need for interventions with the ability to adapt to local settings. Participants stated that team members entering scores should have the opportunity to comment and suggest actions to improve the prospects for sustainability. They suggested that comments could be brought together for each factor to provide a starting point for action planning based on team member ideas. These suggestions were brought forward to adapt the factor language and design the scoring mechanism of the tool.

Interviews allowed detailed views from diverse stakeholders to be identified. Stakeholders unanimously expressed a desire for a tool that is simple to use and quick to complete: *"Brevity I think is the theme. It is very hard to have yet another form to fill or another algorithm to think about, for people who are already over worked and over stretched."(I3)* Interviewees desired a flexible tool with the option of quick review of the factors with any guidance or supportive text being brief and concise. Participants felt that using reflective statements to illicit an overall rating was a good way to get people thinking and provide an engaging format for the tool: *"I think overall impressions are powerful. You get a general feel and I think that is all you can hope for because otherwise…it will not be possible to make it user friendly" (I4)* The data and outputs used to stimulate discussions needed to be simple to access, interpret, and present back to team members: *"I think most clinicians are familiar with a RAG (Red, Amber, Green) rating system so that would be easy for people to understand quickly." (I7)* From this feedback a draft tool was developed.

Piloting with stakeholders demonstrated that majority of participants completed the tool in the projected 10-15 minute time period. Stakeholders engaged well with the statements within the

For peer review only - http://bmjopen.bmj.com/site/about/guidelines.xhtml

tool, commenting that they provided a simple format to begin consideration on how each factor may impact their initiatives. Participants commented that the tool was easy to use and that the statements and questions enabled good discussion and 'promoted deeper thinking' allowing them to think about things they had not previously considered.

Regular scoring and review of factors was discussed and participants agreed in the necessity of consistently reviewing the changes to sustainability throughout their initiatives. Use every three months was recommended by stakeholders, as they felt this timeframe would be feasible given the ease and design of the tool and the potential for changes and turnover of staff in settings. Participants suggested the addition of a 'don't know' and 'no opinion' option to the tool as they did not want to make a forced choice and rate a factor that they did not have enough information to make an accurate rating. During piloting stakeholders questioned the appropriateness of the term 'sustainability'. Many stakeholders felt that 'sustainability' did not accurately capture the need for potential adaptation of initiatives or the desire to continually improve practice. Stakeholders wanted a term that would include both sustained improvements as well as the long term commitment to improvement. These discussions resulted in the term 'long term success' being used in place of sustainability to represent the aim that stakeholders desired. Feedback was used to iteratively develop the tool, which was then rolled out for wider use by CLAHRC NWL teams in January 2015.

The Long Term Success Tool

Purpose:

The Long Term Success Tool (LTST) aims to support those implementing improvements reflect on 12 key factors to identify risks and prompt actions to increase chances of sustainability over time.

The Factors:

The factors included in the tool are: *Commitment to the improvement, Involvement, Skills and capabilities, Leadership, Team functioning, Resources in place, Evidence of benefits, Progress monitored for feedback and learning, Robust and adaptable processes, Alignment with organisational culture and priorities, Support for improvement, and Alignment with external political and financial environment.* The factors and their effects have been well documented in the literature(1,3,6,27) The presentation and language used to articulate the factors has been carefully developed and adapted with stakeholders to improve ease of understanding, and user-friendliness. The wide range of factors addresses the holistic and multifaceted nature of sustainability with each factor influencing more than one of the sustainability domains (continuing activities, health benefits or capacity built). For example, '*skills and capabilities of those involved*' influences both the training

of staff to build capacity in the workforce and the need to consistently deliver the intervention activities on a day to day basis. '*Evidence of benefits*' communicates the importance of ensuring the intervention delivers the intended benefits for patients as well as the need to communicate these findings with the workforce to support the intervention practices. The 12 factors have been organised within 3 areas; People, Practice and Setting. Table 3 describes the factors and provides the statements for rating and supporting questions included within the tool.

For peer review only

Table 3: Long Term Success Factors: Purpose, Statement for rating and questions to consider

Factor	Purpose	Statement	Addition questions to support reflection
**People**			
**Commitment to the Improvement**	To reflect on both own personal commitment to the initiative and impression of commitment across the team as a whole to the initiative	*My team understands what the project is trying to achieve and believe this work will lead to improved processes and outcomes.*	Do you feel committed to the project? Do you understand what the project is trying to achieve?<br><br>Do you believe that this work will improve processes and outcomes? Do you believe there is reliable evidence (e.g. from literature, guidelines etc.) that the project will produce the desired benefits? Do you think there is commitment across the team as a whole?<br><br>Has a shared aim been established for your project?<br>If you think commitment is lacking, what do you think is the reason for this? What do you think should be done to address this?
**Involvement**	Reflect upon who has been involved and who may need to be engaged further for the initiative to achieve long term success. Asks about personal involvement and contribution and explores the involvement of patients, carers and members of the public who are impacted by the changes being made	*a) I have the opportunity to input into the project and I feel a sense of ownership towards the work. I am able to express my ideas freely which are openly considered by the team.*<br><br>*b) There is wide breadth of involvement from stakeholders including patients and members of the public who regularly feed into the project.*	a) Do you personally feel involved in the project? Are you given the opportunity to express your ideas and recommend changes to the project when necessary?<br><br>b) Do you think the project has involved the right people? Does your project involve patients affected by the improvement? Is there involvement from staff who will be delivering the improvement as part of their day-to day practice? Are the views of these groups taken on board?<br><br>Does the project have a good spread of views, skills and expertise? Are people with knowledge of mental as well as physical health involved? Do you believe involvement can be improved? Are there groups of people you still need to involve?
**Skills and capabilities**	Explores whether the staff and other people delivering the change have the skills to do so successfully and whether training of new members of the team has been planned for	*Staff have the necessary skills to deliver the improvement. Training and development opportunities are available to all staff, volunteers and other people involved.*	Do you feel able to fulfil your role within the project? Do you require further training or education? Do staff who will be delivering the improvement (e.g. frontline or support staff) have the skills to do so consistently and effectively?<br><br>Are new staff informed about the project and their role in it?<br><br>Do you think there are training needs associated with the improvement that should be addressed? What should be done to address to these needs?

For peer review only

**Leadership**	Asks if there is strong leadership in place and if the leaders are approachable, available and able to garner support for the initiative	*My project has supportive and respected leaders and/or champions who advocate for the improvement, communicate the vision, and effectively manage the process.*	Do you believe your project has strong leadership?<br>Are your project leaders actively involved in the project?<br>Are they able to garner support and enthusiasm for the work?<br>Are they available and approachable to members of the team if necessary?<br>Do the project leads effectively communicate the need for the change?<br>How do you think leadership could be strengthened?
**Team functioning:**	Explores the accountability and responsibilities for the workload involved in the initiative and ask if the team is working well together	*My project team is working well together. There are clear responsibilities for individuals and the work is shared across the team and does not rely on particular individuals.*	How well do you feel your project team is working together? Does the project team meet and communicate on a regular basis?<br><br>Have clear roles and responsibilities for project team members been established?<br>In your opinion, are team members fulfilling these roles and responsibilities?<br><br>Are skills and expertise of team members considered and put to use? What do you think can be done to improve team functioning?
**Practice**			
**Resources in place**	Explores if the necessary resources such as staff time, equipment and facilities have been dedicated to the initiative	*My project has financial support that will allow the improvement to achieve long term success. We have the necessary staff, material and equipment and I am given sufficient time to dedicate to the improvement.*	In your opinion, have enough resources been dedicated to support the project?<br><br>Do you believe the financial support provided will allow the improvement the project is trying to achieve to become part of normal working practice in the long term?<br><br>Does the project have enough staff to achieve the project aims? Do staff have enough time to spend on the improvement?<br><br>Are the materials needed (e.g. physical facilities, sites, equipment etc.) available to staff when they need them?<br><br>Are resources needed discussed by the team on a regular basis?<br>What resources do you think are lacking?
**Progress monitored for feedback and learning**	Encourages teams to consider what systems are in place to monitor the initiative overtime and how this information will be used to inform staff of further changes needed	*There is a monitoring system in place that allows the team to collect, manage and regularly review data. Feedback from the project is shared with me and other stakeholders on a regular basis.*	Have measures to enable continuous monitoring for your project been defined by the team?<br><br>Do you think these measures are able to assess the impact of the improvement? Can you suggest any changes to improve this?<br><br>Are these measures regularly assessed? Is this information used to make changes and improve project progress?<br>If the measures show lack of progress are the causes for this investigated?<br><br>Are project members and staff regularly informed about what is working well and what could be better?

For peer review only

**Evidence of benefits**	Asks if and how the benefits of the initiative are communicated to both staff and patients overtime	*There is evidence of benefits emerging from the project and this evidence is regularly communicated and visible to staff and patients.*	Does the evidence for your project include both the impact on physical and mental well-being of patients? Is there evidence (process and outcome measures) that the project is producing the desired impact on patients?<br><br>Is evidence of the projects' impact regularly shared with staff, patients and other stakeholders?<br>If evidence shows lack of progress, does the team investigate reasons for this?
**Robust and adaptable processes**	Reflects upon the need for initiatives to be adapted to local processes and emerging needs. It also asks about the process for recording successes and failures of changes made	*There is the opportunity to adapt the project to reflect local needs, setting and emerging evidence. Adaptations are documented and the successes and failures of changes are reported.*	Is there regular review of how the project is working?<br>How well does the project fit within current practices?<br><br>Do staff and team members need to adapt how they implement the improvement in response to challenges or changing care needs?<br><br>Does your team use PDSA cycles, Statistical Process Control and other quality improvement methods to test and document the changes made to the improvement?
**Setting**			
**Alignment with organisational culture and priorities**	Encourages teams to consider the need to align improvement initiatives to organisational strategies to gain executive buy-in and support as well as have the initiative become part of organisational policies and procedures	*The improvement my project is trying to achieve is aligned with the strategic aims and priorities of the organisation(s) we work within and our work contributes to these aims. Our work is supported by the policies and procedures within the organisation.*	Is the improvement your project is trying to achieve aligned with the organisational priorities?<br>Has this been promoted as something to help further the organisation's aims and priorities?<br><br>From your perspective, how well is the work of the project being integrated into the everyday operations of the organisation?<br><br>Does the project conflict with any other changes taking place within the organisation?<br>What could be done to better align your improvement to these priorities?
**Support for Improvement**	Explores the values and beliefs held within organisations related to continuous improvement and looks at the support given to staff and patients to be involved	*There are values and beliefs in my organisation(s) that emphasise the need to improve. Staff and management are supportive of improvement initiatives and continuous improvement is a priority for the organisation, staff and patients.*	Do you feel continuous improvement is a priority within your organisation?<br><br>Are staff and senior management receptive to improvement initiatives?<br><br>Are you supported by your leaders to participate in the improvement initiatives? Do senior leaders actively participate in improvement projects?

For peer review only

**Alignment with external political and financial environment**	Looks at the need for teams to be aware of the potential political and financial changes that may impact the initiative	*My project exists in a supportive economic and political environment. My team is aware of external pressures and incentives that may influence the project.*	Has your team considered the impact of the external environment on the project? For example are there economic pressures or political developments that may impact the project?<br><br>Is there political support for the implementation of your project?<br><br>Does your project help address external political or economic concerns? Does it contribute to the achievement of political objectives?<br><br>Are there plans to mitigate risks due to the external environment?

For peer review only

How it works:

The LTST is designed to create a platform for people to share their own views on sustainability as well as learn about the different views held within their team and to prompt discussion on any difference in opinion.

To ensure teams are aware of how systems are evolving overtime, teams are encouraged to use the tool approximately every 3 months to assess progress and identify emerging risks continuously. Team members are asked to provide their overall impression of how their team is performing in each factor. Responses are collected on a paper questionnaire form or on the CLAHRC NWL Web Improvement System for Healthcare (WISH).[12] The full paper questionnaire can be found in Appendix B.

For each factor, team members are provided with a statement intended to prompt reflection. Supporting questions are available for each factor if team members would like more detail on what to potentially consider (Table 2). Team members score each factor individually and anonymously using a simple 5 point Likert scale (as well as no opinion and don't know options). Team members provide comments to suggest actions, explanations of their rating or concerns about progress against each factor.

Team scores are then brought together to produce aggregated outputs demonstrating how the initiative is performing against the given factors. Figures 2 and 3 show examples of visual charts produced highlighting risks or differences in opinions or both, as well as don't know and no opinion responses. Table 4 shows an example of aggregated comments and actions highlighted within the tool.

Visual charts and comments are intended to facilitate discussion, bring differences of opinion or concerns into the open, and encourage actions to increase the chances of improvements being sustained. For CLAHRC NWL teams, time is allocated at progress meetings to review scores and plan actions.

Figure 2: Team level graph highlighting areas where the team is doing well, where more work is needed and differences of opinion.

Figure 3: Don't Know/No opinion graph showing areas where the team may need more information

Table 4: Comments and actions provided by team members during scoring which can be a starting point for discussion

Factor	Comments and Actions
Commitment to the	* Clear summary of project components and effects now in place from last time

For peer review only

Improvement	* make sure all stakeholders attend meeting<br>* As a commissioner I didn't understand expectations and my role in the group - others seem very committed.<br>* Need to look at those engaging with the project
Involvement	
a)	* difficulties moving forward as until all stakeholders are engaged - unable to move forward<br>* need to consider who is not involved and who would bring influence and value to the project
b)	* more patient/parent engagement at local level helpful<br>* more needed<br>* Patient and Public Involvement needs to be broadened<br>* No public / patient - don't feel it would be appropriate<br>* Patients/ Patient group and primary care practices poorly represented.
Skills and Capabilities of those involved	* of current clinical staff that I'm aware of<br>* capacity issues potentially can limit progress<br>* more nurse input<br>* not enough nursing staff employed to deliver project currently<br>* needs consultant/ general practitioner and nurse shadowing and specific training<br>* limited number of staff needs expansion

Discussion

The aim of this work was to develop an evidenced-based, relevant and practical tool for sustainability that meets the needs of people in improvement initiatives. The value of receiving ongoing feedback during the development period from those who will use the tool has allowed us to design an approach that has responded to user needs and has addressed issues with language, length, and practicality along the way.

The LTST provides a mechanism for improvement teams to identify risks to sustainability and importantly can create an environment for team members to highlight specific actions to be taken and comment on ways to influence sustainability over time. The tool can be used by clinical staff and improvement teams in practice as it is quick to complete, contains user-friendly language and simple outputs for interpretation and presentation.

The LTST builds on established literature and aligns well with other sustainability models and frameworks with all LTST factors reflected in one or more of the other approaches.(1,2,4,9,18,28–31,12,13,32–35) LTST is distinguished from other approaches by its practical design and ability to draw on team suggestions for action planning. Using participant ideas as a platform for action is a unique feature of the tool that is not present in other tools currently used in this area.

While our view of sustainability as a complex process reinforces that all factors are relevant and necessary to consider throughout initiatives, it has been proposed specific factors need consideration at particular time points: for example, engagement with champions and leaders at an

For peer review only - http://bmjopen.bmj.com/site/about/guidelines.xhtml

'early stage' was seen as key in sustaining services in one report.(36) However, there is a need to revisit and review such factors in the later stages of an initiative as changes such as staff turnover occur. Research is needed to explore this area further. Studying the use of the LTST may highlight actions taken throughout different phases of initiatives which may allow us to explore when and if particular factors are more relevant to sustainability.

Developing a tool for sustainability that is both comprehensive and practical has been a key challenge throughout this work. The ongoing tension between achieving theoretical depth and conceptual rigour along with practicality and real-world value has been evident throughout the process. While attempts have been made to respond to user preferences and create a simple tool, it is important to note that some challenges could not be avoided and require further research. Challenges include judging multiple concepts within every factor. For example within 'Commitment to the improvement' participants are asked to judge both their commitment and the wider team commitment. This can result in a factor being scored differently based on what concepts the scorer has deemed most important. Expanding all factors to individual questions and concepts was considered but required a significant increase to the workload involved in using the tool. To offset this challenge, the tool includes supporting questions (Table 3) to describe the potential items to consider within each factor. The questions serve to clarify what users may want to consider when scoring but are not aimed at producing a precise scoring criteria for each factor. The approach has allowed the tool to remain concise and practical, which is ultimately what users prioritised. Further research is required to study the use and effectiveness of the tool in practice and assess engagement with the method over time.

In order to facilitate the use of the tool by those outside of Northwest London, the tool is freely available in paper form along with a structured excel spreadsheet for data entry to produce automated graphs and charts (Table 5).

Table 5: Information on using the Long Term Success Tool

Using the Long Term Success Tool in Your Setting
The Long Term Success tool has been designed on the CLAHRC NWL WISH system. For those who do not have access to this system, the tool can be used with the Long Term Success Excel Spreadsheet and paper form which can be downloaded with this paper. <br>***The paper version of the tool can be used by individuals and teams. Responses can be input into the spreadsheet which enables users to produce similar graphs and outputs to ones shown in this paper. The spreadsheet enables 8 possible entry points for a team (up to 20 team members) and will aggregate team data overtime for review and action planning. (Appendices B and C)***

For peer review only

Conclusion

The development of the LTST has reinforced the importance of working with stakeholders to design strategies which respond to their needs and preferences and can practicality be implemented in real-world settings. This study provides valuable information on the process of developing a new approach to sustainability that is both conceptually rigorous and practical for use with healthcare improvement teams.

Acknowledgments: The authors would like to thank Dr Alan Poots for his contribution to designing and developing the Long Term Success Tool spreadsheets. We would also like to thank the stakeholders involved throughout the development process.

Competing Interests: None

Author Contribution: LL, CD, JR and DB were involved in conceiving the study. LL conducted the interviews. LL, CD, JR and DB contributed to data collection in the group discussions and piloting. CD and LL conducted the analysis. LL drafted the initial manuscript. CD, JR and DB contributed to revisions and the final draft of the manuscript.

Funding: This work was funded by the National Institute for Health Research in the Collaboration for Leadership in Applied Health Research and Care for Northwest London (CLAHRC) programme.

Disclaimer: This article presents independent research commissioned by the National Institute for Health Research (NIHR) under the Collaborations for Leadership in Applied Health Research and Care (CLAHRC) programme for North West London. The views expressed in this publication are those of the author(s) and not necessarily those of the NHS, the NIHR or the Department of Health.

Ethics statement: Ethics approval was not required for this work as it was part of a service evaluation and improvement project. All participants were academics, NHS staff and/or project team members. All interviewees provided verbal consent for the recording of the interviews and were informed that all data would be anonymised for publication.

Data sharing statement: No further data is available.

For peer review only

References

1. Shediac-Rizkallah MC, Bone LR. Planning for the sustainability of community-based health programs: conceptual frameworks and future directions for research, practice and policy. Health Educ Res [Internet]. 1998 Mar;13(1):87–108. Available from: http://www.ncbi.nlm.nih.gov/pubmed/10178339

2. Sirkin HL, Keenan P, Jackson A. The hard side of change mangement. Harv Bus Rev. 2005;(October):109–18.

3. Stirman SW, Kimberly J, Cook N, Calloway A, Castro F, Charns M. The sustainability of new programs and innovations : a review of the empirical literature and recommendations for future research. Implement Sci [Internet]. BioMed Central Ltd; 2012;7(1):17. Available from: http://www.implementationscience.com/content/7/1/17

4. Bowman CC, Sobo EJ, Asch SM, Gifford AL. Measuring persistence of implementation: QUERI Series. Implement Sci [Internet]. 2008 Jan [cited 2014 Aug 27];3(21). Available from: http://www.pubmedcentral.nih.gov/articlerender.fcgi?artid=2390585&tool=pmcentrez&rendertype=abstract

5. Williams L, Daggett V, Slaven JE, Yu Z, Sager D, Myers J, et al. A cluster-randomised quality improvement study to improve two inpatient stroke quality indicators. BMJ Qual Saf [Internet]. 2015;(August):bmjqs – 2015–004188. Available from: http://qualitysafety.bmj.com/lookup/doi/10.1136/bmjqs-2015-004188

6. Scheirer MA. Is Sustainability Possible? A Review and Commentary on Empirical Studies of Program Sustainability. Am J Eval [Internet]. 2005 Sep 1 [cited 2014 Jun 8];26(3):320–47. Available from: http://aje.sagepub.com/cgi/doi/10.1177/1098214005278752

7. Martin GP, Weaver S, Currie G, Finn R, Mcdonald R. Innovation sustainability in challenging health-care contexts: embedding clinically led change in routine practice. Heal Serv Manag Res. 2012;25(190-199).

8. Doyle C, Howe C, Woodcock T, Myron R, Phekoo K, McNicholas C, et al. Making change last: applying the NHS institute for innovation and improvement sustainability model to healthcare improvement. Implement Sci [Internet]. Implementation Science; 2013 Jan [cited 2014 Oct 5];8(1):127. Available from: http://www.pubmedcentral.nih.gov/articlerender.fcgi?artid=3827618&tool=pmcentrez&rendertype=abstract

9. Feldstein AC, Glasgow RE. A Practical , Robust Implementation for Integrating Research Findings into Practice. Jt Comm J Qual patient Saf. 2008;34(4):228–43.

10. Chambers D a, Glasgow RE, Stange KC. The dynamic sustainability framework: addressing the paradox of sustainment amid ongoing change. Implement Sci

For peer review only

[Internet]. Implementation Science; 2013 Jan [cited 2014 Jul 23];8(1):117. Available from: http://www.pubmedcentral.nih.gov/articlerender.fcgi?artid=3852739&tool=pmcentrez&rendertype=abstract

11. Moullin JC, Sabater-hernández D, Fernandez-llimos F, Benrimoj SI. A systematic review of implementation frameworks of innovations in healthcare and resulting generic implementation framework. Heal Res Policy Syst. 2015;13(16):1–11.

12. Maher L, Gustafson D, Evans A. Sustainability Model and guide. Coventry, UK: NHS Institute for Innovation and Improvement; 2005.

13. Luke D a, Calhoun A, Robichaux CB, Elliott MB, Moreland-Russell S. The Program Sustainability Assessment Tool: a new instrument for public health programs. Prev Chronic Dis [Internet]. 2014;11(3):130184. Available from: http://www.pubmedcentral.nih.gov/articlerender.fcgi?artid=3900326&tool=pmcentrez&rendertype=abstract

14. Reed JE, McNicholas C, Woodcock T, Issen L, Bell D. Designing quality improvement initiatives: the action effect method, a structured approach to identifying and articulating programme theory. BMJ Qual Saf [Internet]. 2014 Oct 15 [cited 2014 Oct 16];1–9. Available from: http://qualitysafety.bmj.com/content/early/2014/10/15/bmjqs-2014-003103.full

15. Reed JE, Card AJ. The problem with Plan-Do-Study-Act cycles. BMJ Qual Saf. 2015;(December):1–6.

16. Waring JJ, Bishop S. Lean healthcare: Rhetoric, ritual and resistance. Soc Sci Med [Internet]. Elsevier Ltd; 2010;71(7):1332–40. Available from: http://dx.doi.org/10.1016/j.socscimed.2010.06.028

17. Taylor MJ, McNicholas C, Nicolay C, Darzi A, Bell D, Reed JE. Systematic review of the application of the plan-do-study-act method to improve quality in healthcare. BMJ Qual Saf [Internet]. 2014 Apr [cited 2014 Mar 21];23(4):290–8. Available from: http://www.ncbi.nlm.nih.gov/pubmed/24025320

18. Johnson K, Hays C, Center H, Daley C. Building capacity and sustainable prevention innovations: a sustainability planning model. Eval Program Plann [Internet]. 2004 May [cited 2014 May 23];27(2):135–49. Available from: http://linkinghub.elsevier.com/retrieve/pii/S0149718904000035

19. Ullrich PM, Sahay A, Stetler CB. Use of implementation theory: A focus on PARIHS. Worldviews Evidence-Based Nurs. 2014;11(1):26–34.

20. Howe C, Randall K, Chalkley S, Bell D. Supporting improvement in a quality collaborative. Br J Healthc Manag. 2013;19(9).

21. Caldwell S, Mays N. Studying policy implementation using a macro, meso and micro frame analysis: the case of the Collaboration for Leadership in Applied Health Research & Care (CLAHRC) programme nationally and in North West London. Heal Res Policy Syst. 15 October. 2012;10(32).

22. Arksey H, O'Malley L. Scoping studies: towards a methodological framework. Int J Soc Res Methodol. 2004;8(1):19–32.

23. Buchanan D, Fitzgerald L, Ketley D, Gollop R, Jones JL, Lamont S Saint, et al. No going

For peer review only

back: A review of the literature on sustaining organizational change. Int J Manag Rev [Internet]. 2005 Sep;7(3):189–205. Available from: http://doi.wiley.com/10.1111/j.1468-2370.2005.00111.x

24. Kitzinger J. Introducing focus groups. BMJ Br Med J. 1995;311:299–302.

25. Onwuegbuzie AJ, Leech NL. Sampling Designs in Qualitative Research : Making the Sampling Process More Public. Qual Rep. 2007;12(2):19–20.

26. Wainwright M, Russell A. Using NVivo Audio-Coding: Practical, Sensorial and Epistemological Considerations. Soc Res Updat. 2010;(60):1–4.

27. NHS Modernisation Agency. Complexity of sustaining healthcare improvements: what have we learned so far : Research into Practice report 13. 2004.

28. Goodman RM, McLeroy KR, Steckler AB, Hoyle RH. Development of level of institutionalization scales for health promotion programs. Health Educ Q. 1993;20(2):161–78.

29. Dale BG, Boaden RJ, Wilcox M, Mcquater RE. Total quality management sustaining audit tool: Description and use. Total Qual Manag [Internet]. 1997 Dec [cited 2014 Jun 27];8(6):395–408. Available from: http://www.tandfonline.com/doi/abs/10.1080/0954412979406

30. Glasgow RE, Vogt TM, Boles SM. Evaluating the public health impact of health promotion interventions: the RE-AIM framework. Am J Public Health [Internet]. 1999 Sep;89(9):1322–7. Available from: http://www.pubmedcentral.nih.gov/articlerender.fcgi?artid=1508772&tool=pmcentrez&rendertype=abstract

31. Gruen RL, Elliott JH, Nolan ML, Lawton PD, Parkhill A, McLaren CJ, et al. Sustainability science: an integrated approach for health-programme planning. Lancet [Internet]. Elsevier Ltd; 2008 Nov 1 [cited 2014 May 27];372(9649):1579–89. Available from: http://www.ncbi.nlm.nih.gov/pubmed/18984192

32. Kaplan HC, Provost LP, Froehle CM, Margolis PA. The Model for Understanding Success in Quality (MUSIQ): building a theory of context in healthcare quality improvement. BMJ Qual Saf [Internet]. BMJ Publishing Group Ltd; 2012 Jan 1 [cited 2012 Sep 11];21(1):13–20. Available from: http://qualitysafety.bmj.com/content/21/1/13.full

33. Johnson K, Fisher D, Wandersman A, Collins D. A Sustainability Toolkit for Prevention: Using getting to Outcomes. Pacific Institute for Research and Evaluation. 2012.

34. Kotter P. Leading change : why transformation efforts fail. Harv Bus Rev. 1995;(March-April):59–67.

35. Damschroder LJ, Aron DC, Keith RE, Kirsh SR, Alexander J a, Lowery JC. Fostering implementation of health services research findings into practice: a consolidated framework for advancing implementation science. Implement Sci [Internet]. 2009 Jan [cited 2014 Jul 10];4:50. Available from: http://www.pubmedcentral.nih.gov/articlerender.fcgi?artid=2736161&tool=pmcentrez&rendertype=abstract

36. Martin GP, Weaver S, Currie G, Mcdonald R. The medium-term sustainability of organisational change in the National Health Service : a comparative case study of

For peer review only

clinically led organisational innovations. 2013.

For peer review only

Fig 2. Factor and Tool iterations

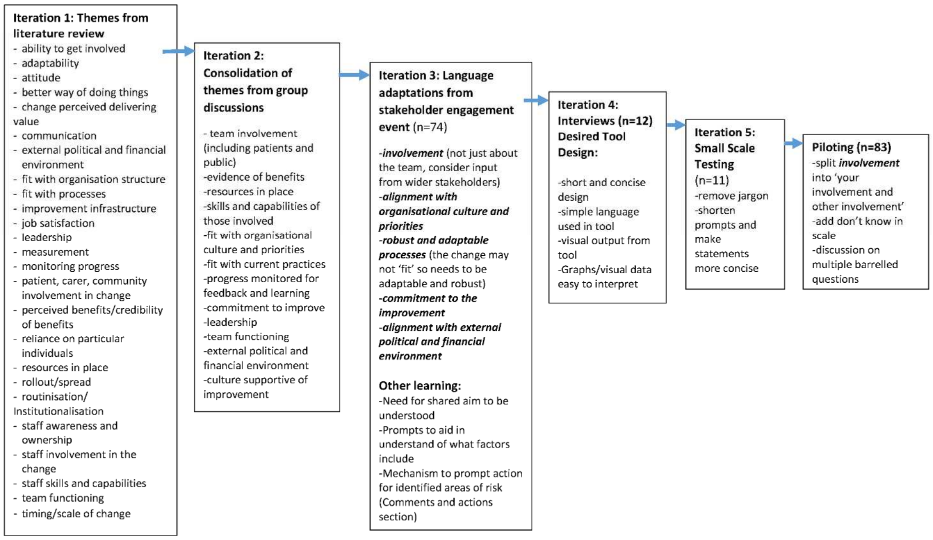

Fig.1

297x210mm (300 x 300 DPI)

iew only

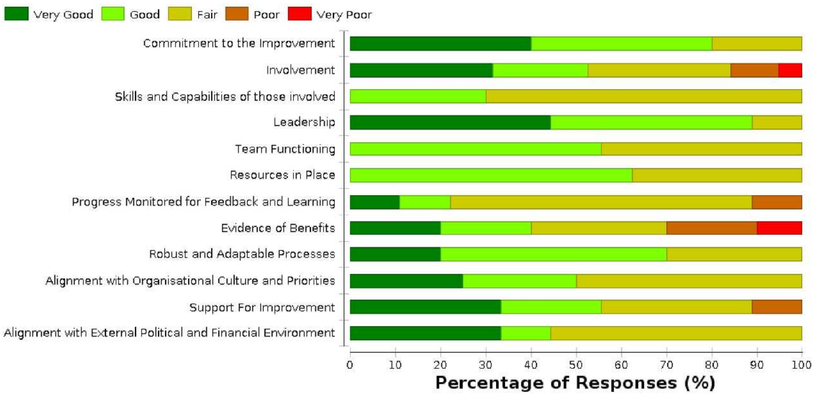

Fig. 2

297x210mm (300 x 300 DPI)

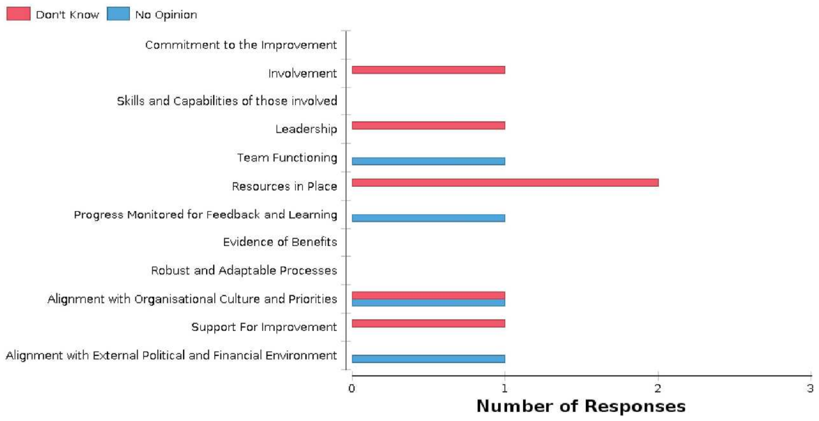

Fig 3

297x210mm (300 x 300 DPI)

Appendix A

Authors	Year	Model/Concept	Purpose	Type	categories identified	Scoring
Goodman	1993	Level of institutionalization (LOIN) scales	Research instrument and diagnostic tool	Scale	8 factor model- 15 three part items. Production routine, Production niche saturation, Maintenance routine, Maintenance niche saturation, supportive routine, Supportive niche saturation, Managerial routine, Managerial niche saturation	Likert scale
Kotter	1995	Anchoring Change	Raise awareness of the common errors made during change initiatives. 8 steps to transforming your organisation.	Model	Establishing a sense of urgency, Formation of a powerful guiding coalition, Creating a vision, Communicating the vision, Empowering others to act on the vision, Planning for and creating short term wins, Consolidating improvements and producing more change, Institutionalising new approaches.	none
Dale et al.	1997	The TQM sustainability audit tool (TQMSAT)	Looks for a specific set of predetermined negative factors; that have been identified as detrimental to sustaining TQM	Tool	Internal/external environment, Management Style, Policies, Organisation structure, Process of change. 23 sub themes including: communication, human resource, improvement infrastructure, education and training	Rate each of the issues on a scale of 1 to 6
Shediac-Rizkallah& Bone	1998	Conceptual framework for planning for sustainability of community based health programs	An organizing framework for conceptualizing and measuring sustainability and tentative guidelines to facilitate sustainability in community programs.	Conceptual Framework	(1) Project design and implementation factors, Project negotiation process. Project effectiveness. Project duration. Project financing Project type, Training<br>(2) factors within the organizational setting: Institutional strength Integration with existing programs/services<br>Program champion/leadership<br>(3) factors in the broader community environment: Socioeconomic and political considerations<br>Community participation.	none
Glasgow et al.	1999	RE-AIM	RE-AIM provides a framework for determining what programs are worth sustained investment and for identifying those that work in real-world environments.	Framework	Reach- patient and staff participation, Efficacy- positive and negative effects of interventions, Adoption-proportion and representation of settings, Implementation-extent to which intervention is delivered as intended and Maintenance-institutionalisation, routine, culture and norms.	0 to 1 (or 0% to 100%) scale.

Appendix A

Johnson et al	2004	A sustainability Planning Model	The model assumes a five-step process (i.e. assessment, development, implementation, evaluation, and reassessment/modification) and addresses factors known to inhibit efforts to sustain an innovation.	Model	Sustainability Capacity, Administrative structures and linkages, Champion roles and leadership actions, resources, policies and procedures, Expertise, Sustainable innovation attributes, Alignment, positive relationships, implementation quality and effectiveness, sustainable actions	none
Sirkin	2005	DICE tool	Allows executives to rate transformation initiatives effectively. Enables organizations to track the progress of projects.	tool	Duration, Integrity of performance, Senior management commitment, Local-level commitment, effort	Likert scale (1-4) Low scores being better.
Edwards	2007	Catholic Healthcare partners HF-GAP Sustainability Assessment (AHRQ)	The checklist is designed to trigger planning for sustainability early in a project's design.	framework and checklist	five components for developing sustainability: identity goals, infrastructure, incentives, incremental opportunities for participation, and integration	Rate your organisation: -1: Problem, 0: Neither positive or negative, 1: Strength
Gruen	2008	Health-programme sustainability	Comprehensive sustainability planning.	A comprehensive approach	Context and resource availability, encompassing health concerns, interventions, and drivers, emphasising dynamic interactions between components	none
Feldstein & Glasgow	2008	A practical robust implementation and sustainability model (PRISM)	a tool to enhance implementation and sustainability. Useful in conceptualizing, implementing, and evaluating health care improvement programs	Model	Program, external environment, Implementation and Sustainability Infrastructure, Recipients with 39 sub-elements to assess	none
Bowman	2008	QUERI Implementation Model	Propose a number of recommendations regarding the design of a sustainability analysis	Framework and model	1) intervention fit, 2) intervention fidelity, 3) intervention dose, and 4) level of the intervention target	none

Appendix A

Johnson et al	2009	A sustainability toolkit for prevention using getting to outcomes	The purpose of this toolkit is to give you a straightforward process to organize, implement, and evaluate an effective plan for sustaining prevention infrastructure and interventions. Based on Johnson's Sustainability model above	Toolkit	Capacity, Infrastructure, Intervention, Routinization, Benefits to diverse populations	none
Damschroder	2009	Consolidated Framework For Implementation Research	Used to guide formative evaluations and build the implementation knowledge base across multiple studies and settings	Framework	8 constructs related to the intervention (e.g., evidence strength and quality), 4 constructs to outer setting (e.g., patient needs and resources), 12 constructs to inner setting (e.g., culture, leadership engagement), 5 constructs related to individual characteristics, and 8 constructs related to process (e.g., plan, evaluate, and reflect)	None
Maher	2010	NHS sustainability Model	To predict the likelihood of sustainability and guide teams to things they could do to increase the chances that the change for improvement will be sustained	Model	10 factors: Staff involvement and training, Staff attitudes towards sustaining the change, Senior leadership engagement, Clinical leadership, Fit with the organisation's strategic aims and culture, Infrastructure for sustainability, Benefits beyond helping patient, Credibility of the benefits, Adaptability of improved process, Effectiveness of the system to monitor progress	Weighted scoring system- Max 30 points
Douglas	2012	The Program Sustainability Assessment Tool	To assess and plan for sustainability risks and Develop an Action Plan with specific action steps	Tool	Political support, funding stability, partnerships organisational capacity, program evaluation, program adaptation, communications, strategic planning	7 point Likert scale (To little or no extent- To a very great extent)
Kaplan	2012	The Model for Understanding Success in Quality (MUSIQ)	A lens to examine the role of context in QI and how context influences QI success	Model	Factors effecting context: External Environment, Organisation, QI support and Capacity, Microsystem, QI Team, Miscellaneous	none

For peer review only - http://bmjopen.bmj.com/site/about/guidelines.xhtml

Long Term Success Tool

This tool aims to aid you in planning for long term success of your work. You will be asked to rate 12 factors that have been identified to impact long term success from current literature and evidence.

Each rating should represent an overall impression of how you believe your project is doing. Please use the boxes to highlight any comments or actions needed to address the factors.

A. Please specify which project you are completing the form for:

B. Please rate the project in the following factors:

1. Commitment to the improvement

My team understands what the project is trying to achieve and believe this work will lead to improved processes and outcomes.

❍ Very Good ❍ Good ❍ Fair ❍ Poor ❍ Very Poor ❍ No Opinion ❍ Don't Know

Comments and actions:

2. Involvement

a) I have the opportunity to input into the project and I feel a sense of ownership towards the work. I am able to express my ideas freely which are openly considered by the team.

❍ Very Good ❍ Good ❍ Fair ❍ Poor ❍ Very Poor ❍ No Opinion ❍ Don't Know

Comments and actions:

b) There is wide breadth of involvement from stakeholders including patients and members of the public who regularly feed into the project.

❍ Very Good ❍ Good ❍ Fair ❍ Poor ❍ Very Poor ❍ No Opinion ❍ Don't Know

Comments and actions:

3. Skills and capabilities

Staff have the necessary skills to deliver the improvement. Training and development opportunities are available to all staff, volunteers and other people involved.

❍ Very Good ❍ Good ❍ Fair ❍ Poor ❍ Very Poor ❍ No Opinion ❍ Don't Know

Comments and actions:

4. Leadership

My project has supportive and respected leaders and/or champions who advocate for the improvement, communicate the vision, and effectively manage the process.

❍ Very Good ❍ Good ❍ Fair ❍ Poor ❍ Very Poor ❍ No Opinion ❍ Don't Know

Comments and actions:

5. Team functioning

My project team is working well together. There are clear responsibilities for individuals and the work is shared across the team and does not rely on particular individuals.

❍ Very Good ❍ Good ❍ Fair ❍ Poor ❍ Very Poor ❍ No Opinion ❍ Don't Know

Comments and actions:

Please turn over

©NIHR CLAHRC
Northwest London

For peer review only - http://bmjopen.bmj.com/site/about/guidelines.xhtml

Long Term Success Tool

6. Resources in place
My project has financial support that will allow the improvement to achieve long term success. We have the necessary staff, material and equipment. I am given sufficient time to dedicate to the improvement.

❍ Very Good ❍ Good ❍ Fair ❍ Poor ❍ Very Poor ❍ No Opinion ❍ Don't Know

Comments and actions:

7. Progress monitored for feedback and learning
There is a monitoring system in place that allows the team to collect, manage and regularly review data. Feedback from the project is shared with me and other stakeholders on a regular basis.

❍ Very Good ❍ Good ❍ Fair ❍ Poor ❍ Very Poor ❍ No Opinion ❍ Don't Know

Comments and actions:

8. Evidence of benefits
There is evidence of benefits emerging from the project and this evidence is regularly communicated and visible to staff and patients.

❍ Very Good ❍ Good ❍ Fair ❍ Poor ❍ Very Poor ❍ No Opinion ❍ Don't Know

Comments and actions:

9. Robust and adaptable processes
There is the opportunity to adapt the project to reflect local needs, setting and emerging evidence. Adaptations are documented and the successes and failures of changes are reported.

❍ Very Good ❍ Good ❍ Fair ❍ Poor ❍ Very Poor ❍ No Opinion ❍ Don't Know

Comments and actions:

10. Alignment with organisational culture and priorities
The improvement my project is trying to achieve is aligned with the strategic aims and priorities of the organisation(s) we work within and our work contributes to these aims. Our work is supported by the policies and procedures within the organisation.

❍ Very Good ❍ Good ❍ Fair ❍ Poor ❍ Very Poor ❍ No Opinion ❍ Don't Know

Comments and actions:

11. Support for improvement
There are values and beliefs in my organisation(s) that emphasise the need to improve. Staff and management are supportive of improvement initiatives and continuous improvement is a priority for the organisation, staff and patients.

❍ Very Good ❍ Good ❍ Fair ❍ Poor ❍ Very Poor ❍ No Opinion ❍ Don't Know

Comments and actions:

12. Alignment with the political and financial environment
My project exists in a supportive economic and political environment. My team is aware of external pressures and incentives that may influence the project.

❍ Very Good ❍ Good ❍ Fair ❍ Poor ❍ Very Poor ❍ No Opinion ❍ Don't Know

Comments and actions:

Thank you for your time

©NIHR CLAHRC
Northwest London

For peer review only

Table 1 Consolidated criteria for reporting qualitative studies (COREQ): 32-item checklist

**No Item Guide questions/description**	**Response**
Personal Characteristics	
1. Interviewer/facilitator: Which author/s conducted the interview or focus group?	Pg. 7
2. Credentials: What were the researcher's credentials? E.g. PhD, MD	RN, MSc PH, PhD Student. Not reported in paper.
3. Occupation: What was their occupation at the time of the study?	Research Assistant, Not reported in paper.
4. Gender: Was the researcher male or female?	Female. Not reported in paper.
5. Experience and training: What experience or training did the researcher have?	4 years research experience, conducting qualitative research. Not reported in paper.
**Relationship with participants**	
6. Relationship established: Was a relationship established prior to study commencement?	No. Not reported in paper.
7. Participant knowledge of the interviewer: What did the participants know about the researcher? e.g. personal goals, reasons for doing the research	Various knowledge based on previous professional encounters. No personal knowledge. Not reported in paper.
8. Interviewer characteristics: What characteristics were reported about the interviewer/facilitator? e.g. Bias, assumptions, reasons and interests in the research topic	PhD student interested in sustainability of improvements. Previous experience of working in healthcare as a registered nursing. Not reported in paper.
**Domain 2: study design**	
Theoretical framework	
9. Methodological orientation and Theory: What methodological orientation was stated to underpin the study? e.g. grounded theory, discourse analysis, ethnography, phenomenology, content analysis	This study was underpinned with thematic content analysis. Pg 7
Participant selection	
10. Sampling: How were participants selected? e.g. purposive, convenience, consecutive, snowball	Purposive Pg 7.
11. Method of approach How were participants approached? e.g. face-to-face, telephone, mail, email	Face-to-face Pg. 7
12. Sample size: How many participants were in the study?	(n=12) Pg 7.
13. Non-participation How many people refused to participate or dropped out? Reasons?	No participants refused. One participant accepted but was unable to meet with in the interview timeline so was replaced with a colleague. Not reported in paper.

For peer review only

Setting	
14. Setting of data collection Where was the data collected? e.g. home, clinic, workplace	Workplace pg.7
15. Presence of non-participants Was anyone else present besides the participants and researchers?	No. Not reported in paper.
16. Description of sample What are the important characteristics of the sample? e.g. demographic data, date	CLAHRC NWL team improvement team members and CLAHRC NWL Staff members Pg. 7
Data collection	
17. Interview guide Were questions, prompts, guides provided by the authors? Was it pilot tested?	Yes. Pg 7.
18. Repeat interviews Were repeat interviews carried out? If yes, how many?	none
19. Audio/visual recording Did the research use audio or visual recording to collect the data?	Audio recorded p.7
20. Field notes Were field notes made during and/or after the interview or focus group?	Yes. Pg. 6, 7 & 8.
21. Duration What was the duration of the interviews or focus group?	30 -60 minutes Pg 7.
22. Data saturation Was data saturation discussed?	Saturation was not the goal of the interviews. Not reported in paper.
23. Transcripts returned Were transcripts returned to participants for comment and/or correction?	No.
**Domain 3: analysis and findings**	
Data analysis	
24. Number of data coders How many data coders coded the data?	1 coder. Main themes discussed with co-authors. Pg 7.
25. Description of the coding tree Did authors provide a description of the coding tree?	Not reported in paper.
26. Derivation of themes Were themes identified in advance or derived from the data?	Derived from the data with content analysis. Pg. 7
27. Software What software, if applicable, was used to manage the data?	Nvivo software was used pg. 7.
28. Participant checking: Did participants provide feedback on the findings?	Participants were able to comment on the findings when given the chance to pilot the tool. Pg. 8.
Reporting	
29. Quotations presented Were participant quotations presented to illustrate the themes / findings? Was each quotation identified? e.g. participant number	Yes pg. 9.
30. Data and findings consistent: Was there consistency between the data presented and the findings?	Yes pg. 9.

For peer review only

31. Clarity of major themes Were major themes clearly presented in the findings?	Yes pg. 9
32. Clarity of minor themes Is there a description of diverse cases or discussion of minor themes?	Not reported.

For peer review only

BMJ Open

What makes a sustainability tool valuable, practical, and useful in real world healthcare practice? A qualitative study on the development of the Long Term Success Tool in Northwest London

For peer review only

Journal:	*BMJ Open*
Manuscript ID	bmjopen-2016-014417.R1
Article Type:	Research
Date Submitted by the Author:	25-May-2017
Complete List of Authors:	Lennox, Laura; CLAHRC for Northwest London, Medicine; Imperial College London, Medicine<br>Doyle, Cathal; NIHR CLAHRC for Northwest London<br>Reed, Julie; NIHR, CLAHRC for NW London<br>Bell, Derek; Imperial College, Acute Medicine
**Primary Subject Heading**:	Health services research
Secondary Subject Heading:	Research methods, Qualitative research
Keywords:	Quality Improvement, Tool, Sustainability

SCHOLARONE™
Manuscripts

Title:

What makes a sustainability tool valuable, practical, and useful in real world healthcare practice? A qualitative study on the development of the Long Term Success Tool in Northwest London

Corresponding author:

Laura Lennox, 369 Fulham Road, Chelsea and Westminster Hospital, London, SW10 9NH. l.lennox@imperial.ac.uk, 0203 315 3392. Imperial College London.

Co-authors:

Cathal Doyle, NIHR CLAHRC Northwest London, United Kingdom
Julie Reed, NIHR CLAHRC Northwest London, United Kingdom
Derek Bell, NIHR CLAHRC Northwest London, United Kingdom

Word count: 4550

For peer review only

For peer review only - http://bmjopen.bmj.com/site/about/guidelines.xhtml

Abstract

Objectives: Although improvement initiatives show benefits to patient care, they often fail to sustain. Models and frameworks exist to address this challenge, but issues with design, clarity and usability have been barriers to use in healthcare settings. This work aimed to collaborate with stakeholders to develop a sustainability tool relevant to people in healthcare settings, and practical for use in improvement initiatives.

Design: Tool development was conducted in 6 stages. A scoping literature review, group discussions, and a stakeholder engagement event explored literature findings and their resonance with stakeholders in this healthcare settings. Interviews, small scale trialling and piloting explored the design and tested the practicality of the tool in improvement initiatives.

Setting: National Institute for Health Research Collaboration for Leadership in Applied Health Research and Care for Northwest London (CLAHRC NWL).

Participants: CLAHRC NWL improvement initiative teams and staff.

Results: The iterative design process and engagement of stakeholders informed the articulation of the sustainability factors identified from the literature and guided tool design for practical application. Key iterations of factors and tool design are discussed. From the development process, The Long Term Success Tool has been designed. The Tool supports those implementing improvements to reflect on 12 sustainability factors to identify risks to increase chances of sustainability overtime. The Tool is designed to provide a platform for improvement teams to share their own views on sustainability as well as learn about the different views held within their team to prompt discussion and actions.

Conclusion: The development of the LTST has reinforced the importance of working with stakeholders to design strategies which respond to their needs and preferences and can practicality be implemented in real-world settings. Further research is required to study the use and effectiveness of the tool in practice and assess engagement with the method over time.

For peer review only

Article summary

Strengths and limitations of this study

- Feedback received from potential users of the Long Term Success tool throughout its development allowed us to design an approach that has responded to user preferences and addressed issues with language, length, and practicality.
- The Long Term Success Tool supports those implementing improvements to reflect on 12 key factors to identify risks and prompt actions to increase chances of sustainability over time.
- The Long Term Success Tool builds on established literature and aligns well with other sustainability models but is distinguished from other approaches by its practical design and ability to draw on team suggestions for action planning.
- A programme of research is currently underway to investigate tool impact on initiative processes and practices and examine actions taken by improvement teams to sustain improvements across diverse settings and environments.
- A systematic review of the literature may have strengthened our approach and uncovered further articles but due to the practical time constraints of our programme this was not possible. The results of our review have fed into a protocol for a full systematic review on available sustainability approaches which is now underway.

For peer review only

For peer review only - http://bmjopen.bmj.com/site/about/guidelines.xhtml

Introduction and Objectives

Significant financial and human resources are invested in initiatives to improve the quality of healthcare and deliver better patient outcomes. While many initiatives show patient benefits or improvements in care processes or clinical outcomes initially (e.g. in the period when resource is available to introduce new practice), these often fail to sustain in the longer term.[1–5] As a result there is growing research interest in this area, with studies showing wide variation in the sustainability of initiatives. Self-reporting measures have reported that up to 60% of programs sustain (at least in part), while studies using more objective measures of sustainability (such as independent observation) report lower rates of sustainability from 6.7% - 45%.[3,6]

This area of research is further complicated by several definitions of sustainability in the literature and little consensus on what constitutes 'achieving sustainability'.[1,7] Despite these issues, three domains of sustainability have been consistently used within the literature; *continuation of initiative activities* (maintenance of the intervention or practices that were introduced), *continuation of the health benefits* which resulted from the initiative (health outcomes remain stable or get better) and *capacity built in the workforce* (the skills gained by being involved in the initiative that can support on-going high quality care or the attainment of skills which enable the workforce to continually improve).[1] Given the complexity and dynamic nature of health care and health care delivery, we believe that all three domains are necessary to define and assess sustainability. For these reasons we have chosen to define sustainability as: a dynamic process where staff and others involved have the capacity and capability to monitor and modify activities and interventions in relation to the health benefits they wish to achieve and in response to threats and opportunities that emerge over time. As sustainability is being seen as a process and not an end point, this definition does not include a specific timeframe for sustainability. Timeframes should be defined by initiative teams and stakeholders and be based on the goals of the improvement initiative with respect to the intervention, desired outcomes, disease area and setting.

Navigating the relationship between achieving initial 'successful' implementation and achieving long term sustainability is a challenge.[1,8–10] It has been noted that over 60% of implementation frameworks include sustainability stages.[11] Factors contributing to sustainability of improvement initiatives often relate to how the improvement initiative is planned and conducted from the outset, suggesting an interdependent relationship between factors that influence initial success and those that influence long term success.[1,8,9] Although the evidence shows an overlap in factors influencing both implementation success and sustainability, there is lack of clarity on what conditions may result in initial success but may or may not result in the sustainability of

For peer review only - http://bmjopen.bmj.com/site/about/guidelines.xhtml

improvements. For example, an initiative may achieve initial success by providing extra resource or putting pressure on the workforce, but once the resource or pressure are removed the benefits achieved are not sustained.

Addressing Sustainability in Practice:

In the current healthcare climate of increasing demands and competing priorities for resources, healthcare planners and stakeholders are increasingly concerned with the long term impact of their investments.[3,10] This has highlighted a need to understand how sustainability of improvement initiatives can be influenced and how specific approaches may help support sustainability.[3,10]

Defined procedures for addressing sustainability in improvement initiatives do not exist but many have suggested that sustainability indicators or factors can be used to monitor and influence sustainability over-time.[1,4,12–14] Multiple strategies and approaches such as models and frameworks have been created to highlight such factors but issues with tool design and content have been identified as barriers to their use in healthcare settings.[10,15–18] Specifically, poorly designed constructs, inadequate coverage of items, and lack of clear definitions have impacted application and outcomes in past use.[15–18] Using methods well in practice is a recognised challenge for improvement teams, highlighting the need for all methods to be designed to be practical for use in real-world healthcare settings.[19–22]

The application of one sustainability method, the NHS Institute for Innovation and Improvement Sustainability Model (SM), has been previously described.[8,23] The SM is a self-assessment tool that details key factors that increase the likelihood of sustainability and continuous improvement.[24] The model is used to raise awareness of 10 factors for sustainability, and prompt teams to consider actions to increase the likelihood of sustainability.[24] Application of this model demonstrated that while the SM raised awareness of determinants of sustainability and was perceived as valuable, teams found it difficult to understand and to apply the model routinely.[8,23] In particular, concerns were raised about the clarity the language used within the model, the user-friendliness of design, the length of time taken to complete the questions and suitability for continuous use in healthcare settings.[8]

The purpose of this study was to collaborate with stakeholders to develop a sustainability tool relevant to people in healthcare settings, and practical for use in improvement initiatives. In order to inform the tool development we explored the following research questions:

For peer review only - http://bmjopen.bmj.com/site/about/guidelines.xhtml

1. How do sustainability factors identified in the literature resonate with the experience of those in improvement projects in healthcare?
2. What features or characteristics make a sustainability tool valuable, practical and useful in real world healthcare practice?

Design

Setting: Research was conducted within the National Institute for Health Research (NIHR) Collaboration for Leadership in Applied Health Research and Care for Northwest London (CLAHRC NWL).[25] CLAHRC NWL improvement projects cover a range of health problems and disease areas that include primary care, secondary care, and community settings that are delivered over 18-24 months with the aim of sustaining improvements beyond this period. To support multidisciplinary teams to implement changes CLAHRC NWL systematically applies Quality Improvement (QI) methods such as the Model for Improvement and Action Effect Method.[19,23] The approach previously included use of the SM (2008-2013) but following internal evaluation and published research, it was acknowledged that a new more user-friendly method for sustainability was required to meet the needs of improvement teams.[8,23]

Participant Information: Participants in this study included members of CLAHRC NWL improvement initiative teams and staff. These members come from various backgrounds: multi-disciplinary healthcare practitioners (doctors, nurses, allied healthcare professionals), patients, carers, healthcare managers, directors, analysts and researchers (many participants hold overlapping roles ie. nurse who is also a project manager). Other participants were also included at the engagement event and piloting. Although the majority of attendance is from improvement teams, these event were are open to the public so additional participants included students, fellows, community members and industry partners. Specific participation from these groups in is outlined within each development stage and summarised in the results.

Methods: Tool development was conducted in 6 stages. The first 3 stages: scoping review, group discussions, and the stakeholder engagement event focussed on reviewing the literature findings and their resonance with stakeholders in this setting. The last 3 stages: interviews, small scale trialling and piloting contributed to designing and testing usability of the tool. The researchers within this study had participant observer roles.[26] They provided teaching, facilitation, and explanation throughout the development stages.

For peer review only - http://bmjopen.bmj.com/site/about/guidelines.xhtml

I. **Scoping Literature Review:** A scoping literature review was undertaken to examine the extent, range and nature of research activity related to sustainability approaches.[27] The *research question* guiding this review was: 'what approaches have been proposed to assess sustainability in healthcare and what sustainability factors are examined in each method'? *Identifying relevant studies:* A number of reviews had previously been published to identify factors for sustainability.[3,6,28] These reviews were used as a starting point to identify relevant authors and publications including snowballing of relevant journal articles, reference lists and the PubMed options of 'similar article' and 'cited by-' articles. *Selecting studies:* We sought approaches (published models, tools, strategies, and frameworks) that identified sustainability factors and themes. Papers that introduced or described a sustainability approach were included. Commentary, posters, protocols, conference proceedings, editorials and perspectives were excluded. *Charting the data:* One author (LL) screened the retrieved papers for inclusion and extracted the data from the articles. Data extraction was independently checked against the full-text articles by a second author (CD). Any differences were discussed and inclusion and exclusion criteria were refined to reflect these discussions. *Summarizing the results:* All sustainability constructs (factors, questions, criteria etc.) identified in the approaches were extracted for thematic analysis. Aggregate themes were developed by combining similar or overlapping concepts and removing duplicate or redundant labels. Overarching sustainability themes were created using a mapping software.[29]

II. **Group discussions:** Three facilitated group discussions were held with CLAHRC NWL team members to understand the perceived relevance of the literature review results against CLAHRC NWL team expertise and experience. Discussions were held during a weekly CLAHRC NWL meeting between core staff. The themes from the scoping review were provided on paper hand-outs to the attendees and an open discussion took place to determine the resonance, and clarity of the themes presented. Observation notes were taken during group discussions. Notes were transcribed and findings were discussed amongst the research team to inform iterations of language and representation of themes which were iteratively adapted and presented at consecutive discussions.

III. **Stakeholder engagement event:** Consolidated sustainability themes were presented to stakeholders at a CLAHRC NWL Collaborative Learning event in April 2014 to check the relevance and language against stakeholder views. In facilitated group discussions, participants provided their views on the resonance of these themes as well as identified any

For peer review only - http://bmjopen.bmj.com/site/about/guidelines.xhtml

For peer review only

missing themes not seen in the literature. Designated note takers captured key learning and suggestions from the discussions. Field notes were collected and transcribed by one researcher. Findings were summarised and fed back to the research team to inform next steps and tool iteration.

IV. **Interviews:** Interviews aimed to collect in-depth information on value and practicality of tool design. A purposive sampling strategy was used to recruit interviewees. Participants were selected based on their role within diverse CLAHRC NWL improvement projects, their level of knowledge of their project, and their experience with the SM (we sought both those with and without experience in using the SM to ensure we had a balanced sample of those with prior opinions of the SM). This approach aimed to maximize the diversity of perspectives gained from the interviews.[30] All interviews were carried out face-to-face in a workplace setting by one author (LL). A semi-structured interview guide was used for all interviews. Interview questions explored the design of questions and statements used to draw attention to factors for sustainability as well as views on collating and presenting data to facilitate discussion and action. Interviews were audio recorded and uploaded onto qualitative software Nvivo (version 9). Audio recordings were coded directly on Nvivo using thematic content analysis.[31] A preliminary coding structure was developed using the interview questions as coding nodes, with themes inductively derived to summarise responses and record patterns in the data. The coding structure was iteratively developed, integrated and refined as further interviews were added to the dataset.[32] Results have been summarized using descriptive summaries and example quotes with explicit links to source text.

V. **Small scale trialling:** A group of individuals involved leading QI projects as part of a CLAHRC NWL fellowship programme were asked to trial a draft version of the tool. Trialling with this group aimed to understand the practical application of the tool including the approximate amount of time to complete by a wide range of people with diverse experience and expertise in improvement initiatives. Each participant filled out the tool for their own QI project. After completion, the group discussed the experience and posed questions on use. Critical feedback and suggestions for tool development were recorded as observation notes and summarised by the research team to inform tool iterations and piloting.

For peer review only - http://bmjopen.bmj.com/site/about/guidelines.xhtml

VI. **Piloting:** The resulting tool was piloted in July 2014. Piloting aimed to provide an opportunity for further comments and suggestions on practicality of the tool in healthcare settings, and to measure if the tool could be completed within an acceptable timeframe. A brief presentation given to participants to outline tool design and instructions for use. Participants were asked to fill out the tool for their individual QI projects. Individuals without a formal project were asked to fill out the tool with a hypothetical project in mind. Participants were given 15 minutes to complete the tool and a 20 minute facilitated group discussion followed. Designated note takers recorded key observations and feedback to inform tool iteration.

Results

Each development stage allowed for iterative adaptation and refinement of concepts, content, and design of the tool. Key iterations from each stage are summarized in Figure 1. The number and roles of participants is outlined in Table 1. The following section discusses results from each development stage and concludes with an introduction to the resultant tool.

Fig 1. Tool Development Stage and Iterations

Table 1: Number of Participants by Roles

Number of Participants by Role								
**Development stage**	**Multi-disciplinary Healthcare Practitioner**	**Healthcare or Project Manager**	**Patient or Carer**	**Researcher or academic**	**Student/ Fellow**	**Data Analyst**	**Other**	**Total**
**I. Scoping Review**	-	-	-	-	-	-	-	0
**II. Group Discussions**	5	9	0	3	-	3	2	22
**III. Stakeholder Engagement Event**	22	12	10	8	7	1	7	74
**IV. Interviews**	6	3	1	-	-	-	2	12
**V. Small Scale testing**	6	1	3	1	11	-	-	11
**VI. Piloting**	30	16	17	6	8	-	20	106

For peer review only - http://bmjopen.bmj.com/site/about/guidelines.xhtml

For peer review only

I. **Scoping review:** The scoping review identified 81 publications with 35 articles retrieved in full text for full documentary analysis. In total 16 publications which identified sustainability approaches were included in this review. Thematic analysis identified 25 overarching themes impacting sustainability (Fig.1). Appendix A summarises the approaches found and the key sustainability constructs extracted. Results demonstrated reasonable consensus in the literature on factors influencing sustainability. The review uncovered themes not explicitly covered in the SM such as considering *resources for the improvement*, and *the impact of the wider environment* on initiatives. No strategy explicitly included the importance of involving patients or carers as an aspect of sustainability which was an key finding from previous CLAHRC NWL work.[8]

II. **Group Discussions:** In total 22 individuals participated in the internal CLAHRC NWL group discussions. Discussions lead to combining themes that had different labels but were seen as having related or overlapping definitions. Discussions also identified where themes may be confusing and need to be expanded to underlying concepts to be relevant to improvement setting. For example, the literature theme of 'staff skills and capabilities' was expanded to include skills and capabilities of all those involved which may include as patients, carers or other stakeholders who participate in QI projects. Academic jargon and terms were also removed such as 'routinisation' which were seen as unhelpful or potentially confusing. These discussions resulted in changes to the language used and theme consolidation to form a list of 12 factors impacting sustainability.(Fig 1)

III. **Stakeholder Engagement Event:** These factors were presented to stakeholders (n=74) in April 2014. The majority of the factors resonated well with stakeholders and were recognised as relevant to healthcare settings but in some cases the factor language needed to be adapted to align with stakeholder expertise and understanding. For example, the factor, 'Fit with Current Practice' was found to be problematic for participants. Although this factor was meant to convey the importance of interventions being aligned with current practice, many stakeholders mentioned that often improvements must be different from the current ways of working so trying to fit in with 'current practices' would not be desirable or possible. The factor was changed to 'Robust and Adaptable Processes' highlighting the need for interventions with the ability to adapt to local settings.

Stakeholders also identified missing concepts and concepts they felt were not clearly represented in the current factors. For example, *establishing a shared aim* for a project was suggested as an explicit prompt underlying the factor 'commitment to the improvement'.

For peer review only - http://bmjopen.bmj.com/site/about/guidelines.xhtml

Desirable design elements were also highlighted. Participants stated that team members entering scores should have the opportunity to comment and suggest actions to improve the prospects for sustainability. They suggested that comments could be brought together for each factor to provide a starting point for action planning based on team member ideas. Suggested changes were used to adapt language and definitions as well as inform design of the scoring mechanism of the tool.

IV. **Interviews:** Interviews (n=12) allowed detailed views from diverse stakeholders to be identified. Interviewees discussed sustainability measurement, tool value and functionality.
Stakeholders unanimously expressed a desire for a tool that is simple to use and quick to complete: "*Brevity I think is the theme. It is very hard to have yet another form to fill or another algorithm to think about, for people who are already over worked and over stretched*."(I3)

Interviewees desired a flexible tool with the option of quick review of the factors with any guidance or supportive text being brief and concise. Participants felt that using reflective statements to illicit an overall rating was a good way to get people thinking and provide an engaging format for the tool: "*I think overall impressions are powerful. You get a general feel and I think that is all you can hope for because otherwise…it will not be possible to make it user friendly" (I4)*

The data and outputs used to stimulate discussions needed to be simple to access, interpret, and present back to team members: *"I think most clinicians are familiar with a RAG (Red, Amber, Green) rating system so that would be easy for people to understand quickly." (I7)* From this feedback a draft tool was developed.

V. **Small Scale Trailing:** CLAHRC NWL fellows (n=11) trialled a draft version of the tool in June 2014. Each fellow was undertaking a QI project across diverse topic areas and settings (for example, service redesign, app development, patient experience measurement and staff training package development). Trialling the tool resulted in refinement the tool's prompt text to reduce the overall length. Stakeholders commented that the tool was a good reminder what to consider for sustainability but suggested changes to some of the language within the tool to remove terms perceived as 'jargon'. For example, in the factor 'Resources in place' original prompt text read: '*I am given sufficient **headspace** and **time** to dedicate to the improvement',* after discussion the term *'headspace'* was removed as it was seen as confusing to some participants. All participants completed the tool within 15 minutes. This timeframe was discussed and seen as acceptable,

For peer review only - http://bmjopen.bmj.com/site/about/guidelines.xhtml

with the fellows commenting that no more than 15 minutes should be allotted for routine tool use in practice.

VI. **Piloting:** Piloting tool place with 106 participants (83 of which returned a completed tool to the research team). Fifty-two participants indicated that were involved in active QI projects. This included 9 CLAHRC NWL QI projects across diverse topics (such as Sickle Cell Disease, Allergic conditions in children, Polypharmacy in the Elderly, Chronic Obstructive Pulmonary Disease and Congestive Heart failure) as well as 19 projects outside of the CLAHRC NWL programme. Piloting with stakeholders demonstrated that majority of participants completed the tool in the projected 10-15 minute time period. Stakeholders engaged well with the prompts within the tool, commenting that they provided a simple format to begin consideration on how each factor may impact their initiatives. Participants commented that the tool was easy to use and that the statements and questions enabled good discussion and 'promoted deeper thinking' allowing them to think about things they had not previously considered.

Regular scoring and review of factors was discussed and participants agreed in the necessity of consistently reviewing the changes to sustainability throughout their initiatives. Use every three months was recommended by stakeholders, as they felt this timeframe would be feasible given the ease and design of the tool and the potential for changes and turnover of staff in settings. Participants suggested the addition of a 'don't know' and 'no opinion' option to the tool as they did not want to make a forced choice and rate a factor that they did not have enough information to make an accurate rating. During piloting stakeholders questioned the appropriateness of the term 'sustainability'. Many stakeholders felt that 'sustainability' did not accurately capture the need for potential adaptation of initiatives or the desire to continually improve practice. Stakeholders wanted a term that would include both sustained improvements as well as the long term commitment to improvement. These discussions resulted in the term 'long term success' being used in place of sustainability to represent the aim that stakeholders desired. Feedback was used to iteratively develop the tool, which was then rolled out for wider use by CLAHRC NWL teams in January 2015. The final design of the tool and description for use is discussed below.

The Long Term Success Tool

For peer review only - http://bmjopen.bmj.com/site/about/guidelines.xhtml

Purpose: The Long Term Success Tool (LTST) aims to support those implementing improvements reflect on 12 key factors to identify risks and prompt actions to increase chances of sustainability over time.

The Factors: The factors included in the tool are: *Commitment to the improvement, Involvement, Skills and capabilities, Leadership, Team functioning, Resources in place, Evidence of benefits, Progress monitored for feedback and learning, Robust and adaptable processes, Alignment with organisational culture and priorities, Support for improvement, and Alignment with external political and financial environment.* The factors and their effects have been well documented in the literature[1,3,6,33] The presentation and language used to articulate the factors has been carefully developed and adapted with stakeholders to improve ease of understanding, and user-friendliness. The 12 factors have been organised within 3 emergent areas; People, Practice and Setting. Table 2 describes the factors and provides the statements for rating and supporting questions included within the tool.

For peer review only

For peer review only - http://bmjopen.bmj.com/site/about/guidelines.xhtml

Table 2: Long Term Success Factors: Purpose, Statement for rating and questions to consider

Factor	Purpose	Statement	Addition questions to support reflection
**People**			
**1. Commitment to the Improvement**	To reflect on both own personal commitment to the initiative and impression of commitment across the team as a whole to the initiative	*My team understands what the project is trying to achieve and believe this work will lead to improved processes and outcomes.*	Do you feel committed to the project? Do you understand what the project is trying to achieve?<br><br>Do you believe that this work will improve processes and outcomes? Do you believe there is reliable evidence (e.g. from literature, guidelines etc.) that the project will produce the desired benefits? Do you think there is commitment across the team as a whole?<br><br>Has a shared aim been established for your project?<br>If you think commitment is lacking, what do you think is the reason for this? What do you think should be done to address this?
**2. Involvement**	Reflect upon who has been involved and who may need to be engaged further for the initiative to achieve long term success. Asks about personal involvement and contribution and explores the involvement of patients, carers and members of the public who are impacted by the changes being made	*a) I have the opportunity to input into the project and I feel a sense of ownership towards the work. I am able to express my ideas freely which are openly considered by the team.*<br><br>*b) There is wide breadth of involvement from stakeholders including patients and members of the public who regularly feed into the project.*	a) Do you personally feel involved in the project? Are you given the opportunity to express your ideas and recommend changes to the project when necessary?<br><br>b) Do you think the project has involved the right people? Does your project involve patients affected by the improvement? Is there involvement from staff who will be delivering the improvement as part of their day-to day practice? Are the views of these groups taken on board?<br><br>Does the project have a good spread of views, skills and expertise? Are people with knowledge of mental as well as physical health involved? Do you believe involvement can be improved? Are there groups of people you still need to involve?
**3. Skills and capabilities**	Explores whether the staff and other people delivering the change have the skills to do so successfully and whether training of new members of the team has been planned for	*Staff have the necessary skills to deliver the improvement. Training and development opportunities are available to all staff, volunteers and other people involved.*	Do you feel able to fulfil your role within the project? Do you require further training or education? Do staff who will be delivering the improvement (e.g. frontline or support staff) have the skills to do so consistently and effectively?<br><br>Are new staff informed about the project and their role in it?<br><br>Do you think there are training needs associated with the improvement that should be addressed? What should be done to address to these needs?

For peer review only - http://bmjopen.bmj.com/site/about/guidelines.xhtml

**4. Leadership**	Asks if there is strong leadership in place and if the leaders are approachable, available and able to garner support for the initiative	*My project has supportive and respected leaders and/or champions who advocate for the improvement, communicate the vision, and effectively manage the process.*	Do you believe your project has strong leadership?<br>Are your project leaders actively involved in the project?<br>Are they able to garner support and enthusiasm for the work?<br>Are they available and approachable to members of the team if necessary?<br>Do the project leads effectively communicate the need for the change?<br>How do you think leadership could be strengthened?
**5. Team functioning:**	Explores the accountability and responsibilities for the workload involved in the initiative and ask if the team is working well together	*My project team is working well together. There are clear responsibilities for individuals and the work is shared across the team and does not rely on particular individuals.*	How well do you feel your project team is working together? Does the project team meet and communicate on a regular basis?<br><br>Have clear roles and responsibilities for project team members been established?<br>In your opinion, are team members fulfilling these roles and responsibilities?<br><br>Are skills and expertise of team members considered and put to use? What do you think can be done to improve team functioning?
**Practice**			
**6. Resources in place**	Explores if the necessary resources such as staff time, equipment and facilities have been dedicated to the initiative	*My project has financial support that will allow the improvement to achieve long term success. We have the necessary staff, material and equipment and I am given sufficient time to dedicate to the improvement.*	In your opinion, have enough resources been dedicated to support the project?<br><br>Do you believe the financial support provided will allow the improvement the project is trying to achieve to become part of normal working practice in the long term?<br><br>Does the project have enough staff to achieve the project aims? Do staff have enough time to spend on the improvement?<br><br>Are the materials needed (e.g. physical facilities, sites, equipment etc.) available to staff when they need them?<br><br>Are resources needed discussed by the team on a regular basis?<br>What resources do you think are lacking?
**7. Progress monitored for feedback and learning**	Encourages teams to consider what systems are in place to monitor the initiative overtime and how this information will be used to inform staff of further changes needed	*There is a monitoring system in place that allows the team to collect, manage and regularly review data. Feedback from the project is shared with me and other stakeholders on a regular basis.*	Have measures to enable continuous monitoring for your project been defined by the team?<br><br>Do you think these measures are able to assess the impact of the improvement? Can you suggest any changes to improve this?<br><br>Are these measures regularly assessed? Is this information used to make changes and improve project progress?<br>If the measures show lack of progress are the causes for this investigated?<br><br>Are project members and staff regularly informed about what is working well and what could be better?

For peer review only - http://bmjopen.bmj.com/site/about/guidelines.xhtml

**8. Evidence of benefits**	Asks if and how the benefits of the initiative are communicated to both staff and patients overtime	*There is evidence of benefits emerging from the project and this evidence is regularly communicated and visible to staff and patients.*	Does the evidence for your project include both the impact on physical and mental well-being of patients? Is there evidence (process and outcome measures) that the project is producing the desired impact on patients?<br><br>Is evidence of the projects' impact regularly shared with staff, patients and other stakeholders?<br>If evidence shows lack of progress, does the team investigate reasons for this?
**9. Robust and adaptable processes**	Reflects upon the need for initiatives to be adapted to local processes and emerging needs. It also asks about the process for recording successes and failures of changes made	*There is the opportunity to adapt the project to reflect local needs, setting and emerging evidence. Adaptations are documented and the successes and failures of changes are reported.*	Is there regular review of how the project is working?<br>How well does the project fit within current practices?<br><br>Do staff and team members need to adapt how they implement the improvement in response to challenges or changing care needs?<br><br>Does your team use PDSA cycles, Statistical Process Control and other quality improvement methods to test and document the changes made to the improvement?
**Setting**			
**10. Alignment with organisational culture and priorities**	Encourages teams to consider the need to align improvement initiatives to organisational strategies to gain executive buy-in and support as well as have the initiative become part of organisational policies and procedures	*The improvement my project is trying to achieve is aligned with the strategic aims and priorities of the organisation(s) we work within and our work contributes to these aims. Our work is supported by the policies and procedures within the organisation.*	Is the improvement your project is trying to achieve aligned with the organisational priorities?<br>Has this been promoted as something to help further the organisation's aims and priorities?<br><br>From your perspective, how well is the work of the project being integrated into the everyday operations of the organisation?<br><br>Does the project conflict with any other changes taking place within the organisation?<br>What could be done to better align your improvement to these priorities?
**11. Support for Improvement**	Explores the values and beliefs held within organisations related to continuous improvement and looks at the support given to staff and patients to be involved	*There are values and beliefs in my organisation(s) that emphasise the need to improve. Staff and management are supportive of improvement initiatives and continuous improvement is a priority for the organisation, staff and patients.*	Do you feel continuous improvement is a priority within your organisation?<br><br>Are staff and senior management receptive to improvement initiatives?<br><br>Are you supported by your leaders to participate in the improvement initiatives? Do senior leaders actively participate in improvement projects?

For peer review only - http://bmjopen.bmj.com/site/about/guidelines.xhtml

**12. Alignment with external political and financial environment**	Looks at the need for teams to be aware of the potential political and financial changes that may impact the initiative	*My project exists in a supportive economic and political environment. My team is aware of external pressures and incentives that may influence the project.*	Has your team considered the impact of the external environment on the project? For example are there economic pressures or political developments that may impact the project?<br><br>Is there political support for the implementation of your project?<br><br>Does your project help address external political or economic concerns? Does it contribute to the achievement of political objectives?<br><br>Are there plans to mitigate risks due to the external environment?

For peer review only

For peer review only - http://bmjopen.bmj.com/site/about/guidelines.xhtml

How it works:

The LTST is designed to create a platform for people to share their own views on sustainability as well as learn about the different views held within their team and to prompt discussion on any difference in opinion. To ensure teams are aware of how systems are evolving overtime, teams are encouraged to use the tool approximately every 3 months to assess progress and identify emerging risks continuously. Team members are asked to provide their overall impression of how their team is performing in each factor. Responses are collected on a paper questionnaire form or on the CLAHRC NWL Web Improvement System for Healthcare (WISH).[34] The full paper questionnaire can be found in Appendix B.

For each factor, team members are provided with a statement intended to prompt reflection. Supporting questions are available for each factor if team members would like more detail on what to potentially consider (Table 2). Team members score each factor individually and anonymously using a simple 5 point Likert scale (as well as no opinion and don't know options). Team members provide comments to suggest actions, explanations of their rating or concerns about progress against each factor.

Team scores are then brought together to produce aggregated outputs demonstrating how the initiative is performing against the given factors. Figure 2 shows an example a visual chart produced highlighting risks and differences in opinions. Table 3 shows an example of aggregated comments and actions highlighted within the tool.

Visual charts and comments are intended to facilitate discussion, bring differences of opinion or concerns into the open, and encourage actions to increase the chances of improvements being sustained. For CLAHRC NWL teams, time is allocated at progress meetings to review scores and plan actions.

Figure 2: Team level graph highlighting areas where the team is doing well, where more work is needed and differences of opinion.

Table 3: Comments and actions provided by team members during scoring which can be a starting point for discussion

Factor	Comments and Actions
Commitment to the Improvement	* Clear summary of project components and effects now in place from last time<br>* make sure all stakeholders attend meeting<br>* As a commissioner I didn't understand expectations and my role in the group - others seem very committed.<br>* Need to look at those engaging with the project
Involvement	
a)	* difficulties moving forward as until all stakeholders are engaged - unable to move forward

	* need to consider who is not involved and who would bring influence and value to the project
b)	* More patient/parent engagement at local level helpful<br>* more needed<br>* Patient and Public Involvement needs to be broadened<br>* No public / patient - don't feel it would be appropriate<br>* Patients/ Patient group and primary care practices poorly represented.
Skills and Capabilities of those involved	* of current clinical staff that I'm aware of<br>* capacity issues potentially can limit progress<br>* more nurse input<br>* not enough nursing staff employed to deliver project currently<br>* needs consultant/ general practitioner and nurse shadowing and specific training<br>* limited number of staff needs expansion

For peer review only

Discussion

The aim of this work was to develop a relevant and practical tool for sustainability that meets the needs of people in improvement initiatives. We explored how sustainability factors identified in the literature resonated with those in improvement projects and the features or characteristics which make a sustainability tool most valuable in real world healthcare practice. This work has shown that the majority of factors from the literature resonated well with stakeholders and were recognised as relevant to healthcare settings. In some cases, the literature findings needed to be adapted through changes to the language used to align with stakeholder preferences and understanding. Engaging stakeholder in the design process demonstrated that stakeholders valued clarity, conciseness, and simplicity for tool design with simple data interpretation and visual graphs. Receiving ongoing feedback during the development period from those who will use the tool has allowed us to design an approach that has responded to user needs and has addressed issues with language, length, and practicality along the way.

The LTST provides a mechanism for improvement teams to identify risks to sustainability and importantly can create an environment for team members to highlight specific actions to be taken and comment on ways to influence sustainability over time. The LTST builds on established literature and aligns well with other sustainability models and frameworks with all LTST factors reflected in one or more of the other approaches.[1,2,4,9,24,35–44] LTST is distinguished from other approaches by its practical design and ability to draw on team suggestions for action planning. Using participant ideas as a platform for action is a unique feature of the tool that is not present in other tools currently used in this area. Also unique to the LTST is that the allotted time for use, a identified barrier and challenge to other method use, has been explicitly tested and informed by end-users.[8,45] While many other methods involve either unknown or substantial time commitments, the LTST can be completed in approximately 10-15 minutes.[42,45]

There is also potential to supplement the use of other models or frameworks to complement the LTST. For example, if a project receives a low rating for the factor 'Robust and Adaptable Processes', The Model for Highly Adoptable Improvement toolkit may be used to aid the team in further understanding of where the intervention can be adapted.[46]

Limitations: A limitation of this work is the use of a snowballing scoping review opposed to a systematic review. Conducting a full systematic review may have uncovered further articles and/or approaches but due to the practical time constraints of our programme this was not possible. The results of our review have fed into a protocol for a full systematic review on available sustainability approaches which is now underway.[47] The results of this review will inform future adaptation of the LTST.

Another limitation of this work is the potential for responder bias throughout development stages. Prior relationships between researchers and participants was identified as a possible source of bias, namely, social desirability bias, as participants may have responder in ways that were seen as more desirable to the researchers.[48] As the development of the tool was centred on user preferences attempts were made to communicate and reiterate there were no 'right' answer to questions. We also attempted to mitigate this effect by having multiple stages for feedback, with diverse facilitators and a wide variety of participants. We also had a researcher unknown to the majority of the interviewees conduct the interviews.

Future Research: While attempts have been made to respond to user preferences and create a practical tool, further research is required to assess tool effectiveness and engagement over time. A 3-year programme of research with teams at CLAHRC NWL and other groups internationally is currently underway to investigate tool impact on initiative processes and practices and examine actions taken by improvement teams to sustain improvements across diverse settings and environments. This longitudinal study will also investigate tool links to sustainability outcomes to assess what impact tool use may have on sustained QI projects. In order to facilitate and study the use of the tool by those outside of Northwest London, the tool is freely available along with a structured excel spreadsheet for data entry to produce automated graphs and charts (Table 4).

Table 4: Information on using the Long Term Success Tool

Using the Long Term Success Tool in Your Setting
The Long Term Success tool has been designed on the CLAHRC NWL WISH system. For those who do not have access to this system, the Long Term Success Tool questionnaire form and Excel spreadsheet can be downloaded with this paper. The tool can be used along with Table 2 which

For peer review only

provides supporting questions to describe the potential items to consider within each factor. The tool can be used by individuals and teams. Responses can be input into the Excel spreadsheet which enables users to produce similar graphs and outputs to ones shown in this paper. The spreadsheet enables 8 possible entry points for a team (up to 20 team members) and will aggregate team data overtime for review and action planning. (Appendices B and C)

Conclusion

The development of the LTST has reinforced the importance of working with stakeholders to design strategies which respond to their needs and preferences and can practicality be implemented in real-world settings. This study provides valuable information on the process of developing a new approach to sustainability that is both conceptually rigorous and practical for use with healthcare improvement teams.

Acknowledgments: The authors would like to thank Dr Alan Poots for his contribution to designing and developing the Long Term Success Tool spreadsheets. We would also like to thank the stakeholders involved throughout the development process.

Competing Interests: None

Author Contribution: LL, CD, JR and DB were involved in conceiving the study. LL conducted the interviews. LL, CD, JR and DB contributed to data collection in the group discussions and piloting. CD and LL conducted the analysis. LL drafted the initial manuscript. CD, JR and DB contributed to revisions and the final draft of the manuscript.

Funding: This work was funded by the National Institute for Health Research in the Collaboration for Leadership in Applied Health Research and Care for Northwest London (CLAHRC) programme.

Disclaimer: This article presents independent research commissioned by the National Institute for Health Research (NIHR) under the Collaborations for Leadership in Applied Health Research and Care (CLAHRC) programme for North West London. The views expressed in this publication are those of the author(s) and not necessarily those of the NHS, the NIHR or the Department of Health.

Ethics statement: Ethics approval was not required for this work as it was part of a service evaluation and improvement project. All interviewees provided verbal consent for the recording of the interviews and were informed that all data would be anonymised for publication.

Data sharing statement: No further data is available.

For peer review only

References

1 Shediac-Rizkallah MC, Bone LR. Planning for the sustainability of community-based health programs: conceptual frameworks and future directions for research, practice and policy. *Health Educ Res* 1998;**13**:87–108.

2 Sirkin HL, Keenan P, Jackson A. The hard side of change mangement. *Harv Bus Rev* 2005;:109–18.

3 Stirman SW, Kimberly J, Cook N, *et al.* The sustainability of new programs and innovations : a review of the empirical literature and recommendations for future research. *Implement Sci* 2012;**7**:17. doi:10.1186/1748-5908-7-17

4 Bowman CC, Sobo EJ, Asch SM, *et al.* Measuring persistence of implementation: QUERI Series. *Implement Sci* 2008;**3**. doi:10.1186/1748-5908-3-21

5 Williams L, Daggett V, Slaven JE, *et al.* A cluster-randomised quality improvement study to improve two inpatient stroke quality indicators. *BMJ Qual Saf* 2015;:bmjqs-2015-004188. doi:10.1136/bmjqs-2015-004188

6 Scheirer MA. Is Sustainability Possible? A Review and Commentary on Empirical Studies of Program Sustainability. *Am J Eval* 2005;**26**:320–47. doi:10.1177/1098214005278752

7 Martin GP, Weaver S, Currie G, *et al.* Innovation sustainability in challenging health-care contexts: embedding clinically led change in routine practice. *Heal Serv Manag Res* 2012;**25**. doi:10.1177/0951484812474246

8 Doyle C, Howe C, Woodcock T, *et al.* Making change last: applying the NHS institute for innovation and improvement sustainability model to healthcare improvement. *Implement Sci* 2013;**8**:127. doi:10.1186/1748-5908-8-127

9 Feldstein AC, Glasgow RE. A Practical , Robust Implementation for Integrating Research Findings into Practice. *Jt Comm J Qual patient Saf* 2008;**34**:228–43.

10 Chambers D a, Glasgow RE, Stange KC. The dynamic sustainability framework: addressing the paradox of sustainment amid ongoing change. *Implement Sci* 2013;**8**:117. doi:10.1186/1748-5908-8-117

11 Moullin JC, Sabater-hernández D, Fernandez-llimos F, *et al.* A systematic review of implementation frameworks of innovations in healthcare and resulting generic implementation framework. *Heal Res Policy Syst* 2015;**13**:1–11. doi:10.1186/s12961-015-0005-z

12 Scheirer MA, Dearing JW. An agenda for research on the sustainability of public health programs. *Am J Public Health* 2011;**101**:2059–67. doi:10.2105/AJPH.2011.300193

13 Wiek A, Ness B, Schweizer-Ries P, *et al.* From complex systems analysis to transformational change: A comparative appraisal of sustainability science projects. *Sustain Sci* 2012;**7**:5–24. doi:10.1007/s11625-011-0148-y

14 Grol RPTM, Bosch MC, Hulscher MEJL, *et al.* Planning and Studying Improvement in Patient Care: The Use of Theoretical Perspectives. *Milbank Q* 2007;**85**:93–138.

15 Ullrich PM, Sahay A, Stetler CB. Use of implementation theory: A focus on PARIHS. *Worldviews Evidence-Based Nurs* 2014;**11**:26–34. doi:10.1111/wvn.12016

For peer review only

16 Drew S, Judge A, May C, *et al.* Implementation of secondary fracture prevention services after hip fracture: a qualitative study using extended Normalization Process Theory. *Implement Sci* 2015;**10**:57.

17 Leon N, Lewin S, Mathews C, *et al.* Implementing a provider-initiated testing and counselling (PITC) intervention in Cape town, South Africa: a process evaluation using the normalisation process model. *Implement Sci* 2013;**8**:97.

18 Chilundo BGM, Cliff JL, Mariano ARE, *et al.* Relaunch of the official community health worker programme in Mozambique : is there a sustainable basis for iCCM policy ? 2015;:54–64. doi:10.1093/heapol/czv036

19 Reed JE, McNicholas C, Woodcock T, *et al.* Designing quality improvement initiatives: the action effect method, a structured approach to identifying and articulating programme theory. *BMJ Qual Saf* 2014;:1–9.

20 Reed JE, Card AJ. The problem with Plan-Do-Study-Act cycles. *BMJ Qual Saf* 2015;:1–6. doi:10.1136/bmjqs-2015-005076

21 Waring JJ, Bishop S. Lean healthcare: Rhetoric, ritual and resistance. *Soc Sci Med* 2010;**71**:1332–40. doi:10.1016/j.socscimed.2010.06.028

22 Taylor MJ, McNicholas C, Nicolay C, *et al.* Systematic review of the application of the plan-do-study-act method to improve quality in healthcare. *BMJ Qual Saf* 2014;**23**:290–8. doi:10.1136/bmjqs-2013-001862

23 Howe C, Randall K, Chalkley S, *et al.* Supporting improvement in a quality collaborative. *Br J Healthc Manag* 2013;**19**.

24 Maher L, Gustafson D, Evans A. *Sustainability Model and guide*. Coventry, UK: : NHS Institute for Innovation and Improvement 2005.

25 Caldwell S, Mays N. Studying policy implementation using a macro, meso and micro frame analysis: the case of the Collaboration for Leadership in Applied Health Research & Care (CLAHRC) programme nationally and in North West London. *Heal Res Policy Syst* 2012;**10**. doi:doi:10.1186/1478-4505-10-32

26 Gold R. Roles in sociological field observation. *Soc Forces* 1958;**36**:213–7.

27 Arksey H, O'Malley L. Scoping studies: towards a methodological framework. *Int J Soc Res Methodol* 2004;**8**:19–32.

28 Buchanan D, Fitzgerald L, Ketley D, *et al.* No going back: A review of the literature on sustaining organizational change. *Int J Manag Rev* 2005;**7**:189–205. doi:10.1111/j.1468-2370.2005.00111.x

29 Ltd Xm. XMind. 2016.

30 Onwuegbuzie AJ, Leech NL. Sampling Designs in Qualitative Research : Making the Sampling Process More Public. *Qual Rep* 2007;**12**:19–20. doi:10.1007/s11135-006-9000-3

31 Wainwright M, Russell A. Using NVivo Audio-Coding: Practical, Sensorial and Epistemological Considerations. *Soc Res Updat* 2010;:1–4. doi:10.1177/1473325010009002100

32 Elo S, Kyngäs H. The qualitative content analysis process. *J Adv Nurs* 2008;**62**:107–15. doi:10.1111/j.1365-2648.2007.04569.x

For peer review only

33 NHS Modernisation Agency. Complexity of sustaining healthcare improvements: what have we learned so far : Research into Practice report 13. 2004.

34 Curcin V, Woodcock T, Poots AJ, *et al.* Model-driven approach to data collection and reporting for quality improvement. *J Biomed Inform* 2014;**52**:151–62.

35 Goodman RM, McLeroy KR, Steckler AB, *et al.* Development of level of institutionalization scales for health promotion programs. *Health Educ Q* 1993;**20**:161–78.

36 Dale BG, Boaden RJ, Wilcox M, *et al.* Total quality management sustaining audit tool: Description and use. *Total Qual Manag* 1997;**8**:395–408. doi:10.1080/0954412979406

37 Glasgow RE, Vogt TM, Boles SM. Evaluating the public health impact of health promotion interventions: the RE-AIM framework. *Am J Public Health* 1999;**89**:1322–7.

38 Johnson K, Hays C, Center H, *et al.* Building capacity and sustainable prevention innovations: a sustainability planning model. *Eval Program Plann* 2004;**27**:135–49. doi:10.1016/j.evalprogplan.2004.01.002

39 Gruen RL, Elliott JH, Nolan ML, *et al.* Sustainability science: an integrated approach for health-programme planning. *Lancet* 2008;**372**:1579–89. doi:10.1016/S0140-6736(08)61659-1

40 Luke D a, Calhoun A, Robichaux CB, *et al.* The Program Sustainability Assessment Tool: a new instrument for public health programs. *Prev Chronic Dis* 2014;**11**:130184. doi:10.5888/pcd11.130184

41 Kaplan HC, Provost LP, Froehle CM, *et al.* The Model for Understanding Success in Quality (MUSIQ): building a theory of context in healthcare quality improvement. *BMJ Qual Saf* 2012;**21**:13–20.

42 Johnson K, Fisher D, Wandersman A, *et al.* A Sustainability Toolkit for Prevention: Using getting to Outcomes. 2012. doi:10.1073/pnas.0703993104

43 Kotter P. Leading change : why transformation efforts fail. *Harv Bus Rev* 1995;:59–67.

44 Damschroder LJ, Aron DC, Keith RE, *et al.* Fostering implementation of health services research findings into practice: a consolidated framework for advancing implementation science. *Implement Sci* 2009;**4**:50. doi:10.1186/1748-5908-4-50

45 Hooker L, Small R, Humphreys C, *et al.* Applying normalization process theory to understand implementation of a family violence screening and care model in maternal and child health nursing practice: a mixed method process evaluation of a randomised controlled trial. *Implement Sci* 2015;**10**:39.

46 Hayes CW, Batalden PB, Goldmann D. A 'work smarter, not harder' approach to improving healthcare quality. *BMJ Qual Saf* 2014;**24**:100–2. doi:10.1136/bmjqs-2014-003673

47 Lennox L, Maher L, Bell D, *et al.* The sustainability landscape: a systematic review of sustainability strategies and their impact in healthcare settings. *PROSPERO Int Prospect Regist Syst Rev* 2016;**CRD4201604**:1–4.

48 Kalton G, Schuman H. The Effect of the Question on Survey Responses: A Review. *J R Stat Soc* 1982;**145**:42–73.

For peer review only

Figure Legend:

Figure	Page
**Figure 1.** Tool Development Stage and Iterations	9
**Figure 2:** Team level graph highlighting areas where the team is doing well, where more work is needed and differences of opinion	18

For peer review only

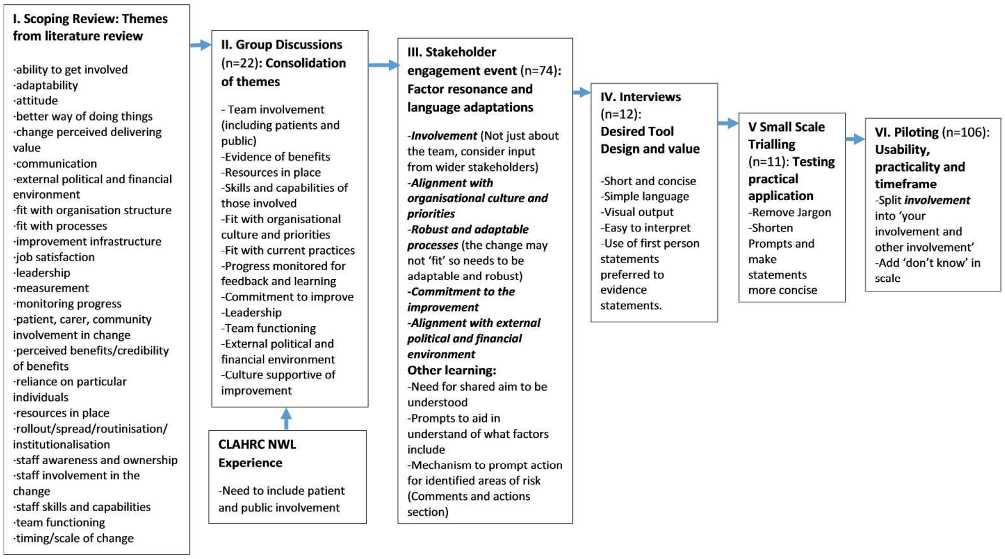

Fig 1

261x144mm (300 x 300 DPI)

review only

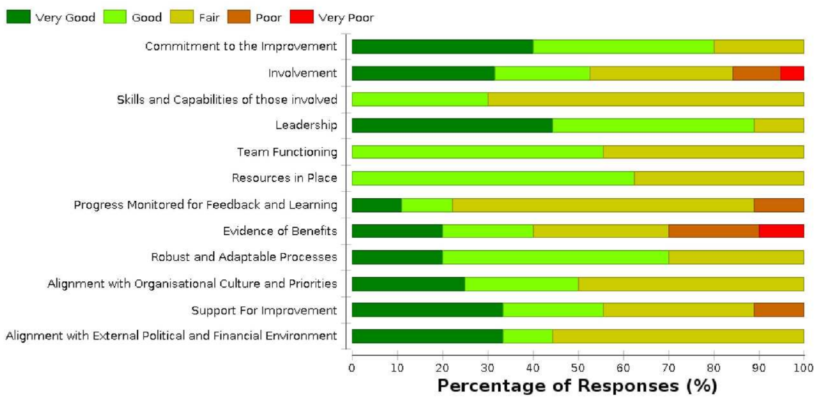

Fig. 2

297x210mm (300 x 300 DPI)

Appendix A

Authors	Year	Model/Concept	Purpose	Type	Sustainability Constructs Identified	Scoring
Goodman	1993	Level of institutionalization (LOIN) scales	Research instrument and diagnostic tool	Scale	8 factor model- 15 three part items. Production routine, Production niche saturation, Maintenance routine, Maintenance niche saturation, supportive routine, Supportive niche saturation, Managerial routine, Managerial niche saturation	Likert scale
Kotter	1995	Anchoring Change	Raise awareness of the common errors made during change initiatives. 8 steps to transforming your organisation.	Model	Establishing a sense of urgency, Formation of a powerful guiding coalition, Creating a vision, Communicating the vision, Empowering others to act on the vision, Planning for and creating short term wins, Consolidating improvements and producing more change, Institutionalising new approaches.	none
Dale et al.	1997	The TQM sustainability audit tool (TQMSAT)	Looks for a specific set of predetermined negative factors; that have been identified as detrimental to sustaining TQM	Tool	Internal/external environment, Management Style, Policies, Organisation structure, Process of change. 23 sub themes including: communication, human resource, improvement infrastructure, education and training	Rate each of the issues on a scale of 1 to 6
Shediac-Rizkallah& Bone	1998	Conceptual framework for planning for sustainability of community based health programs	An organizing framework for conceptualizing and measuring sustainability and tentative guidelines to facilitate sustainability in community programs.	Conceptual Framework	(1) Project design and implementation factors, Project negotiation process. Project effectiveness. Project duration. Project financing Project type, Training<br>(2) Factors within the organizational setting: Institutional strength Integration with existing programs/services Program champion/leadership<br>(3) Factors in the broader community environment: Socioeconomic and political considerations Community participation.	none
Glasgow et al.	1999	RE-AIM	RE-AIM provides a framework for determining what programs are worth sustained investment and for identifying those that work in real-world environments.	Framework	Reach- patient and staff participation, Efficacy- positive and negative effects of interventions, Adoption-proportion and representation of settings, Implementation-extent to which intervention is delivered as intended and Maintenance-institutionalisation, routine, culture and norms.	0 to 1 (or 0% to 100%) scale.

Appendix A

Johnson et al	2004	A sustainability Planning Model	The model assumes a five-step process (i.e. assessment, development, implementation, evaluation, and reassessment/modification) and addresses factors known to inhibit efforts to sustain an innovation.	Model	Sustainability Capacity, Administrative structures and linkages, Champion roles and leadership actions, resources, policies and procedures, Expertise, Sustainable innovation attributes, Alignment, positive relationships, implementation quality and effectiveness, sustainable actions	none
Sirkin	2005	DICE tool	Allows executives to rate transformation initiatives effectively. Enables organizations to track the progress of projects.	tool	Duration, Integrity of performance, Senior management commitment, Local-level commitment, effort	Likert scale (1-4) Low scores being better.
Edwards	2007	Catholic Healthcare partners HF-GAP Sustainability Assessment (AHRQ)	The checklist is designed to trigger planning for sustainability early in a project's design.	framework and checklist	Five components for developing sustainability: identity goals, infrastructure, incentives, incremental opportunities for participation, and integration	Rate your organisation: -1: Problem, 0: Neither positive or negative, 1: Strength
Gruen	2008	Health-programme sustainability	Comprehensive sustainability planning.	A comprehensive approach	Context and resource availability, encompassing health concerns, interventions, and drivers, emphasising dynamic interactions between components	none
Feldstein & Glasgow	2008	A practical robust implementation and sustainability model (PRISM)	a tool to enhance implementation and sustainability. Useful in conceptualizing, implementing, and evaluating health care improvement programs	Model	Program, external environment, Implementation and Sustainability Infrastructure, Recipients with 39 sub-elements to assess	none
Bowman	2008	QUERI Implementation Model	Propose a number of recommendations regarding the design of a sustainability analysis	Framework and model	1) intervention fit, 2) intervention fidelity, 3) intervention dose, and 4) level of the intervention target	none

Appendix A

Johnson et al	2009	A sustainability toolkit for prevention using getting to outcomes	The purpose of this toolkit is to give you a straightforward process to organize, implement, and evaluate an effective plan for sustaining prevention infrastructure and interventions. Based on Johnson's Sustainability model above	Toolkit	Capacity, Infrastructure, Intervention, Routinization, Benefits to diverse populations	none
Damschroder	2009	Consolidated Framework For Implementation Research	Used to guide formative evaluations and build the implementation knowledge base across multiple studies and settings	Framework	8 constructs related to the intervention (e.g., evidence strength and quality), 4 constructs to outer setting (e.g., patient needs and resources), 12 constructs to inner setting (e.g., culture, leadership engagement), 5 constructs related to individual characteristics, and 8 constructs related to process (e.g., plan, evaluate, and reflect)	None
Maher	2010	NHS sustainability Model	To predict the likelihood of sustainability and guide teams to things they could do to increase the chances that the change for improvement will be sustained	Model	10 factors: Staff involvement and training, Staff attitudes towards sustaining the change, Senior leadership engagement, Clinical leadership, Fit with the organisation's strategic aims and culture, Infrastructure for sustainability, Benefits beyond helping patient, Credibility of the benefits, Adaptability of improved process, Effectiveness of the system to monitor progress	Weighted scoring system- Max 30 points
Douglas	2012	The Program Sustainability Assessment Tool	To assess and plan for sustainability risks and Develop an Action Plan with specific action steps	Tool	Political support, funding stability, partnerships organisational capacity, program evaluation, program adaptation, communications, strategic planning	7 point Likert scale (To little or no extent- To a very great extent)
Kaplan	2012	The Model for Understanding Success in Quality (MUSIQ)	A lens to examine the role of context in QI and how context influences QI success	Model	Factors effecting context: External Environment, Organisation, QI support and Capacity, Microsystem, QI Team, Miscellaneous	none

Long Term Success Tool

This tool aims to aid you in planning for long term success of your work. You will be asked to rate 12 factors that have been identified to impact long term success from current literature and evidence.

Each rating should represent an overall impression of how you believe your project is doing. Please use the boxes to highlight any comments or actions needed to address the factors.

A. Please specify which project you are completing the form for:

B. Please rate the project in the following factors:

1. Commitment to the improvement

My team understands what the project is trying to achieve and believe this work will lead to improved processes and outcomes.

❍ Very Good ❍ Good ❍ Fair ❍ Poor ❍ Very Poor ❍ No Opinion ❍ Don't Know

Comments and actions:

2. Involvement

a) I have the opportunity to input into the project and I feel a sense of ownership towards the work. I am able to express my ideas freely which are openly considered by the team.

❍ Very Good ❍ Good ❍ Fair ❍ Poor ❍ Very Poor ❍ No Opinion ❍ Don't Know

Comments and actions:

b) There is wide breadth of involvement from stakeholders including patients and members of the public who regularly feed into the project.

❍ Very Good ❍ Good ❍ Fair ❍ Poor ❍ Very Poor ❍ No Opinion ❍ Don't Know

Comments and actions:

3. Skills and capabilities

Staff have the necessary skills to deliver the improvement. Training and development opportunities are available to all staff, volunteers and other people involved.

❍ Very Good ❍ Good ❍ Fair ❍ Poor ❍ Very Poor ❍ No Opinion ❍ Don't Know

Comments and actions:

4. Leadership

My project has supportive and respected leaders and/or champions who advocate for the improvement, communicate the vision, and effectively manage the process.

❍ Very Good ❍ Good ❍ Fair ❍ Poor ❍ Very Poor ❍ No Opinion ❍ Don't Know

Comments and actions:

5. Team functioning

My project team is working well together. There are clear responsibilities for individuals and the work is shared across the team and does not rely on particular individuals.

❍ Very Good ❍ Good ❍ Fair ❍ Poor ❍ Very Poor ❍ No Opinion ❍ Don't Know

Comments and actions:

Please turn over

©NIHR CLAHRC
Northwest London

For peer review only

Long Term Success Tool

6. Resources in place

My project has financial support that will allow the improvement to achieve long term success. We have the necessary staff, material and equipment. I am given sufficient time to dedicate to the improvement.

❍ Very Good ❍ Good ❍ Fair ❍ Poor ❍ Very Poor ❍ No Opinion ❍ Don't Know

Comments and actions:

7. Progress monitored for feedback and learning

There is a monitoring system in place that allows the team to collect, manage and regularly review data. Feedback from the project is shared with me and other stakeholders on a regular basis.

❍ Very Good ❍ Good ❍ Fair ❍ Poor ❍ Very Poor ❍ No Opinion ❍ Don't Know

Comments and actions:

8. Evidence of benefits

There is evidence of benefits emerging from the project and this evidence is regularly communicated and visible to staff and patients.

❍ Very Good ❍ Good ❍ Fair ❍ Poor ❍ Very Poor ❍ No Opinion ❍ Don't Know

Comments and actions:

9. Robust and adaptable processes

There is the opportunity to adapt the project to reflect local needs, setting and emerging evidence. Adaptations are documented and the successes and failures of changes are reported.

❍ Very Good ❍ Good ❍ Fair ❍ Poor ❍ Very Poor ❍ No Opinion ❍ Don't Know

Comments and actions:

10. Alignment with organisational culture and priorities

The improvement my project is trying to achieve is aligned with the strategic aims and priorities of the organisation(s) we work within and our work contributes to these aims. Our work is supported by the policies and procedures within the organisation.

❍ Very Good ❍ Good ❍ Fair ❍ Poor ❍ Very Poor ❍ No Opinion ❍ Don't Know

Comments and actions:

11. Support for improvement

There are values and beliefs in my organisation(s) that emphasise the need to improve. Staff and management are supportive of improvement initiatives and continuous improvement is a priority for the organisation, staff and patients.

❍ Very Good ❍ Good ❍ Fair ❍ Poor ❍ Very Poor ❍ No Opinion ❍ Don't Know

Comments and actions:

12. Alignment with the political and financial environment

My project exists in a supportive economic and political environment. My team is aware of external pressures and incentives that may influence the project.

❍ Very Good ❍ Good ❍ Fair ❍ Poor ❍ Very Poor ❍ No Opinion ❍ Don't Know

Comments and actions:

Thank you for your time

©NIHR CLAHRC
Northwest London

For peer review only

The Long Term Success Tool- Excel spreadsheet for Data input

Instructions for Use

This tool aims to help you ensure the improvements you are attempting to make with your project deliver lasting benefits to patients.

This tool is designed to encourage reflection on 12 factors identified in the literature as important to long term success and to identify what action your team can take. The tool provides a simple way to rate your impressions on these factors (e.g. to give an overall impression of how your project is doing in relation to each of these factors) and suggest action that is likely to increase the prospects of your work having lasting benefits. Although some factors may feel more relevant at different stages in the project, there is value in considering all of these factors at an early stage.

Your rating may be influenced by how you feel about a particular factor (e.g. do you personally feel committed to this project?) and/or your overall impression of how your team views a factor (e.g. do you get the impression there is commitment across the team?). A comment box is provided for you to explain concerns you may have and to suggest actions for your team.

Your ratings, comments and suggested actions will be combined with those of other team members and an overall graphs representing the team's views will be produced. These outputs can be brought to designated meetings where the team can discuss results and actions that should be taken to improve specific factors.

The tool will take approximately 15 minutes to complete.

How to:

1. Every team member should use the Long Term Success Paper form to score and comment on each factor. The Long Term Success Questions within the framework can be used to support team members in scoring the factors.
2. Team scores should be entered in the Excel spreadsheet for each time point.
3. The scores are aggreagted in included in the automated table and visual charts.
4. Outputs can be used to begin a team conversation on risks and possible next steps to enhance the chances of making lasting improvements.
5. The spreadsheet enables 8 possible entry points for a team (up to 20 team members) and will aggregate team data overtime for review and action planning.

For peer review only

	factor	Commitment to the Improvement	Involvement (A)	Involvement (B)	Skills and Capabilities of those involved	Leadership	Team Functioning	Resources in Place	Progress Monitored for Feedback and Learning	Evidence of Benefits	Robust and Adaptable Processes	Alignment with Organisational Culture and Priorities	Support for Improvement	Alignment with External Political and Financial Environment
person	1	very good	very good	good	very good	very good	very good	good	very good	good	don't know	no opinion	good	good
	2	poor	very poor	fair	fair	fair	poor	poor	don't know	no opinion	good	very good	very good	fair
	3	good	fair	very good	good	poor	fair	good	poor	no opinion	don't know	good	fair	good
	4													
	5													
	6													
	7													
	8													
	9													
	10													
	11													
	12													
	13													
	14													
	15													
	16													
	17													
	18													
	19													
	20													

Comments	Commitment to the Improvement	Involvement (A)	Involvement (B)	Skills and Capabilities of those involved	Leadership	Team Functioning	Resources in Place	Progress Monitored for Feedback and Learning	Evidence of Benefits	Robust and Adaptable Processes	Alignment with Organisational Culture and Priorities	Support for Improvement	Alignment with External Political and Financial Environment
1													
2													
3													
4													
5													
6													
7													
8													
9													
10													
11													
12													
13													
14													
15													
16													
17													
18													
19													
20													

For peer review only - http://bmjopen.bmj.com/site/about/guidelines.xhtml

factor	Commitment to the Improvement	Involvement (A)	Involvement (B)	Skills and Capabilities of those involved	Leadership	Team Functioning	Resources in Place	Progress Monitored for Feedback and Learning	Evidence of Benefits	Robust and Adaptable Processes	Alignment with Organisational Culture and Priorities	Support for Improvement	Alignment with External Political and Financial Environment
very good	1	1	1	1	1	1	0	1	0	0	1	1	0
good	1	0	1	1	0	0	2	0	1	1	1	1	2
fair	0	1	1	1	1	1	0	0	0	0	0	1	1
poor	1	0	0	0	1	1	1	1	0	0	0	0	0
very poor	0	1	0	0	0	0	0	0	0	0	0	0	0
total respons	3	3	3	3	3	3	3	2	1	1	2	3	3
don't know	0	0	0	0	0	0	0	1	0	2	0	0	0
no opinion	0	0	0	0	0	0	0	0	2	0	1	0	0
not able to ra	0	0	0	0	0	0	0	1	2	2	1	0	0
Total answers	3	3	3	3	3	3	3	3	3	3	3	3	3
max	very good	very good	very good	very good	very good	very good	good	very good	good	good	very good	very good	good
min	poor	very poor	fair	fair	poor	poor	poor	poor	good	good	good	fair	fair
MEDIAN	good	fair	good	good	fair	fair	good	G/F	good	good	VG/G	good	good

review only

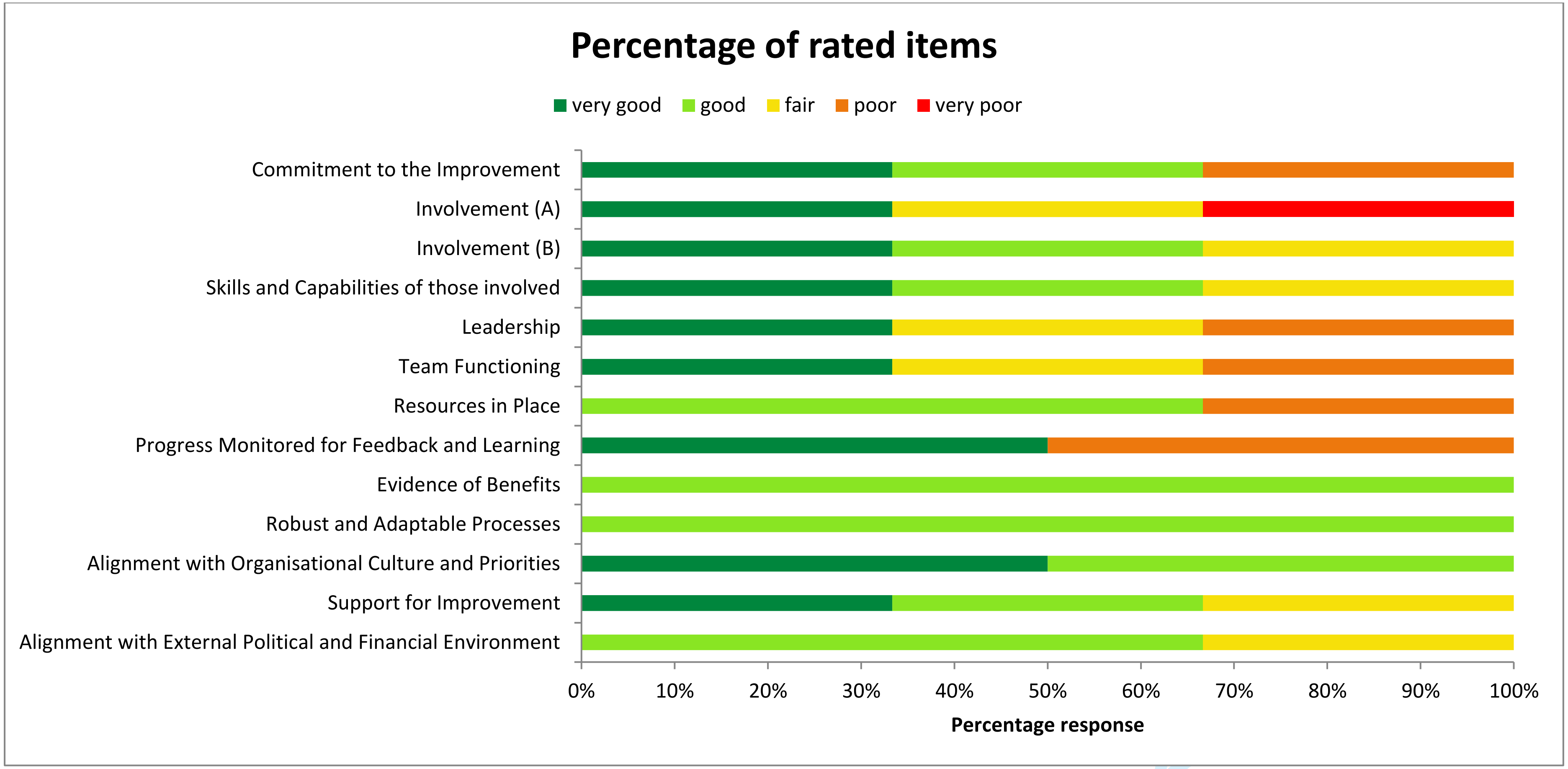
Percentage of rated items
very good
good
fair
poor
very poor
Commitment to the Improvement
Involvement (A)
Involvement (B)
Skills and Capabilities of those involved
Leadership
Team Functioning
Resources in Place
Progress Monitored for Feedback and Learning
Evidence of Benefits
Robust and Adaptable Processes
Alignment with Organisational Culture and Priorities
Support for Improvement
Alignment with External Political and Financial Environment
0%
10%
20%
30%
40%
50%
60%
70%
80%
90%
100%
Percentage response

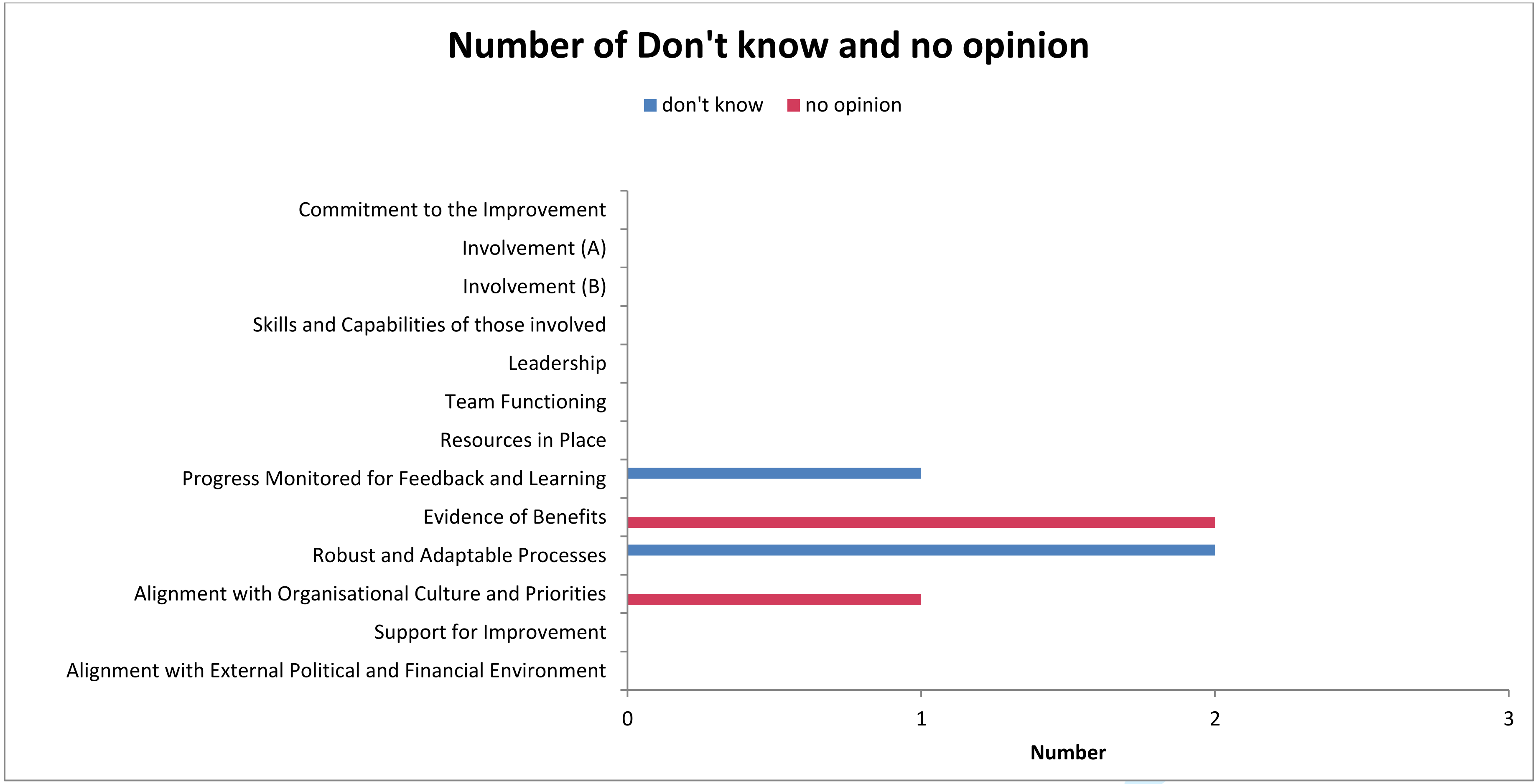
Number of Don't know and no opinion
don't know
no opinion
Commitment to the Improvement
Involvement (A)
Involvement (B)
Skills and Capabilities of those involved
Leadership
Team Functioning
Resources in Place
Progress Monitored for Feedback and Learning
Evidence of Benefits
Robust and Adaptable Processes
Alignment with Organisational Culture and Priorities
Support for Improvement
Alignment with External Political and Financial Environment
0
1
2
3
Number

Consolidated criteria for reporting qualitative studies (COREQ): 32-item checklist

**No Item Guide questions/description**	**Response**
Personal Characteristics	
1. Interviewer/facilitator: Which author/s conducted the interview or focus group?	Pg. 8
2. Credentials: What were the researcher's credentials? E.g. PhD, MD	RN, MSc PH, PhD Student. Not reported in paper.
3. Occupation: What was their occupation at the time of the study?	Research Assistant, Not reported in paper.
4. Gender: Was the researcher male or female?	Female. Not reported in paper.
5. Experience and training: What experience or training did the researcher have?	4 years research experience, conducting qualitative research. Not reported in paper.
**Relationship with participants**	
6. Relationship established: Was a relationship established prior to study commencement?	The researchers were known to and had prior relationships with the participants in the CLAHRC NWL discussion groups. The researcher conducting the interviews had no prior relationship with any of the interviewees besides 2 QI facilitators who worked with teams but were employed within CLAHRC NWL. Page 20
7. Participant knowledge of the interviewer: What did the participants know about the researcher? e.g. personal goals, reasons for doing the research	Various knowledge based on previous professional encounters. No personal knowledge. Not reported in paper.
8. Interviewer characteristics: What characteristics were reported about the interviewer/facilitator? e.g. Bias, assumptions, reasons and interests in the research topic	PhD student interested in sustainability of improvements. Previous experience of working in healthcare as a registered nursing. Not reported in paper.
**Domain 2: study design**	
Theoretical framework	
9. Methodological orientation and Theory: What methodological orientation was stated to underpin the study? e.g. grounded theory, discourse analysis, ethnography, phenomenology, content analysis	This study was underpinned with thematic content analysis. Pg 8
Participant selection	

For peer review only

10. Sampling: How were participants selected? e.g. purposive, convenience, consecutive, snowball	Purposive Pg 8
11. Method of approach How were participants approached? e.g. face-to-face, telephone, mail, email	Face-to-face Pg. 8
12. Sample size: How many participants were in the study?	Pg. 9<br>CLAHRC NWL Group Discussions: (n=22) Pg.<br>Stakeholder engagement event (74)<br>Interviews (n=12)<br>Small scale trialling (n=11)<br>Piloting (n=106)
13. Non-participation How many people refused to participate or dropped out? Reasons?	For interviews, No participants refused. One participant accepted but was unable to meet with in the interview timeline so was replaced with a colleague. Not reported in paper.<br>For the other group stages (discussion event, testing and piloting) participation was voluntary so no formal refusals were noted.
Setting	
14. Setting of data collection Where was the data collected? e.g. home, clinic, workplace	Workplace or public space for engagement event and piloting. Pg. 8
15. Presence of non-participants Was anyone else present besides the participants and researchers?	No. Not reported in paper.
16. Description of sample What are the important characteristics of the sample? e.g. demographic data, date	CLAHRC NWL team improvement team members and CLAHRC NWL Staff members Pg. 6
Data collection	
17. Interview guide Were questions, prompts, guides provided by the authors? Was it pilot tested?	Yes. Pg 8.
18. Repeat interviews Were repeat interviews carried out? If yes, how many?	none
19. Audio/visual recording Did the research use audio or visual recording to collect the data?	Audio recorded p.8
20. Field notes Were field notes made during and/or after the interview or focus group?	Yes. Pg. 7-9.
21. Duration What was the duration of the interviews or focus group?	30 -60 minutes Not reported in paper.
22. Data saturation Was data saturation discussed?	Saturation was not the goal of the interviews. Not reported in paper.

For peer review only

23. Transcripts returned Were transcripts returned to participants for comment and/or correction?	No.
**Domain 3: analysis and findings**	
Data analysis	
24. Number of data coders How many data coders coded the data?	1 coder. Main themes and coding labels discussed with co-authors and amended as needed. Not reported in paper
25. Description of the coding tree Did authors provide a description of the coding tree?	Not reported in paper.
26. Derivation of themes Were themes identified in advance or derived from the data?	Deductively derived from the data with content analysis with a predefined coding structure to respond to interview questions. Pg. 8
27. Software What software, if applicable, was used to manage the data?	Nvivo software was used pg. 8.
28. Participant checking: Did participants provide feedback on the findings?	Participants were able to comment on the findings when given the chance to test and pilot the tool. Pg. 11-12.
Reporting	
29. Quotations presented Were participant quotations presented to illustrate the themes / findings? Was each quotation identified? e.g. participant number	Yes pg. 10.
30. Data and findings consistent: Was there consistency between the data presented and the findings?	Yes pg. 9-12.
31. Clarity of major themes Were major themes clearly presented in the findings?	Yes pg. 10
32. Clarity of minor themes Is there a description of diverse cases or discussion of minor themes?	Not reported in paper

For peer review only

BMJ Open

What makes a sustainability tool valuable, practical, and useful in real world healthcare practice? A mixed methods study on the development of the Long Term Success Tool in Northwest London

Journal:	*BMJ Open*
Manuscript ID	bmjopen-2016-014417.R2
Article Type:	Research
Date Submitted by the Author:	11-Jul-2017
Complete List of Authors:	Lennox, Laura; CLAHRC for Northwest London, Medicine; Imperial College London, Medicine<br>Doyle, Cathal; NIHR CLAHRC for Northwest London<br>Reed, Julie; NIHR, CLAHRC for NW London<br>Bell, Derek; Imperial College, Acute Medicine
**Primary Subject Heading**:	Health services research
Secondary Subject Heading:	Research methods, Qualitative research
Keywords:	Quality Improvement, Tool, Sustainability

SCHOLARONE™
Manuscripts

For peer review only

Title:

What makes a sustainability tool valuable, practical, and useful in real world healthcare practice? A mixed methods study on the development of the Long Term Success Tool in Northwest London

Corresponding author:

Laura Lennox, 369 Fulham Road, Chelsea and Westminster Hospital, London, SW10 9NH. l.lennox@imperial.ac.uk, 0203 315 3392. Imperial College London.

Co-authors:

Cathal Doyle, NIHR CLAHRC Northwest London, United Kingdom
Julie Reed, NIHR CLAHRC Northwest London, United Kingdom
Derek Bell, NIHR CLAHRC Northwest London, United Kingdom

Word count: 4975

For peer review only

For peer review only - http://bmjopen.bmj.com/site/about/guidelines.xhtml

Abstract

Objectives: Although improvement initiatives show benefits to patient care, they often fail to sustain. Models and frameworks exist to address this challenge, but issues with design, clarity and usability have been barriers to use in healthcare settings. This work aimed to collaborate with stakeholders to develop a sustainability tool relevant to people in healthcare settings, and practical for use in improvement initiatives.

Design: Tool development was conducted in 6 stages. A scoping literature review, group discussions, and a stakeholder engagement event explored literature findings and their resonance with stakeholders in this healthcare settings. Interviews, small scale trialling and piloting explored the design and tested the practicality of the tool in improvement initiatives.

Setting: National Institute for Health Research Collaboration for Leadership in Applied Health Research and Care for Northwest London (CLAHRC NWL).

Participants: CLAHRC NWL improvement initiative teams and staff.

Results: The iterative design process and engagement of stakeholders informed the articulation of the sustainability factors identified from the literature and guided tool design for practical application. Key iterations of factors and tool design are discussed. From the development process, The Long Term Success Tool has been designed. The Tool supports those implementing improvements to reflect on 12 sustainability factors to identify risks to increase chances of sustainability overtime. The Tool is designed to provide a platform for improvement teams to share their own views on sustainability as well as learn about the different views held within their team to prompt discussion and actions.

Conclusion: The development of the LTST has reinforced the importance of working with stakeholders to design strategies which respond to their needs and preferences and can practicality be implemented in real-world settings. Further research is required to study the use and effectiveness of the tool in practice and assess engagement with the method over time.

For peer review only - http://bmjopen.bmj.com/site/about/guidelines.xhtml

Article summary

Strengths and limitations of this study

- Feedback received from potential users of the Long Term Success tool throughout its development allowed us to design an approach that has responded to user preferences and addressed issues with language, length, and practicality.
- The Long Term Success Tool builds on established literature and aligns well with other sustainability models but is distinguished from other approaches by its practical design and ability to draw on team suggestions for action planning.
- A systematic review of the literature may have strengthened our approach and uncovered further articles but due to the practical time constraints of our programme this was not possible.
- A limitation of this work is the potential for responder bias throughout development stages.
- Prior relationships between researchers and participants was identified as a possible source of bias, as participants may have responded in ways that were seen as more desirable to the researchers.

For peer review only

For peer review only - http://bmjopen.bmj.com/site/about/guidelines.xhtml

Introduction and Objectives

Significant financial and human resources are invested in initiatives to improve the quality of healthcare and deliver better patient outcomes. While many initiatives show patient benefits or improvements in care processes or clinical outcomes initially (e.g. in the period when resource is available to introduce new practice), these often fail to sustain in the longer term.[1–5] As a result there is growing research interest in this area, with studies showing wide variation in the sustainability of initiatives. Self-reporting measures have reported that up to 60% of programs sustain (at least in part), while studies using more objective measures of sustainability (such as independent observation) report lower rates of sustainability from 6.7% - 45%.[3,6]

This area of research is further complicated by several definitions of sustainability in the literature and little consensus on what constitutes 'achieving sustainability'.[1,7] Despite these issues, three domains of sustainability have been consistently used within the literature; *continuation of initiative activities* (maintenance of the intervention or practices that were introduced), *continuation of the health benefits* which resulted from the initiative (health outcomes remain stable or get better) and *capacity built in the workforce* (the skills gained by being involved in the initiative that can support on-going high quality care or the attainment of skills which enable the workforce to continually improve).[1] Given the complexity and dynamic nature of health care and health care delivery, we believe that all three domains are necessary to define and assess sustainability. For these reasons we have chosen to define sustainability as: a dynamic process where staff and others involved have the capacity and capability to monitor and modify activities and interventions in relation to the health benefits they wish to achieve and in response to threats and opportunities that emerge over time. As sustainability is being seen as a process and not an end point, this definition does not include a specific timeframe for sustainability. Timeframes should be defined by initiative teams and stakeholders and be based on the goals of the improvement initiative with respect to the intervention, desired outcomes, disease area and setting.

Navigating the relationship between achieving initial 'successful' implementation and achieving long term sustainability is a challenge.[1,8–10] It has been noted that over 60% of implementation frameworks include sustainability stages.[11] Factors contributing to sustainability of improvement initiatives often relate to how the improvement initiative is planned and conducted from the outset, suggesting an interdependent relationship between factors that influence initial success and those that influence long term success.[1,8,9] Although the evidence shows an overlap in factors influencing both implementation success and sustainability, there is lack of clarity on what conditions may result in initial success but may or may not result in the sustainability of

For peer review only - http://bmjopen.bmj.com/site/about/guidelines.xhtml

improvements. For example, an initiative may achieve initial success by providing extra resource or putting pressure on the workforce, but once the resource or pressure are removed the benefits achieved are not sustained.

Addressing Sustainability in Practice:

In the current healthcare climate of increasing demands and competing priorities for resources, healthcare planners and stakeholders are increasingly concerned with the long term impact of their investments.[3,10] This has highlighted a need to understand how sustainability of improvement initiatives can be influenced and how specific approaches may help support sustainability.[3,10]

Defined procedures for addressing sustainability in improvement initiatives do not exist but many have suggested that sustainability indicators or factors can be used to monitor and influence sustainability over-time.[1,4,12–14] Multiple strategies and approaches such as models and frameworks have been created to highlight such factors but issues with tool design and content have been identified as barriers to their use in healthcare settings.[10,15–18] Specifically, poorly designed constructs, inadequate coverage of items, and lack of clear definitions have impacted application and outcomes in past use.[15–18] Using methods well in practice is a recognised challenge for improvement teams, highlighting the need for all methods to be designed to be practical for use in real-world healthcare settings.[19–22]

The application of one sustainability method, the NHS Institute for Innovation and Improvement Sustainability Model (SM), has been previously described.[8,23] The SM is a self-assessment tool that details key factors that increase the likelihood of sustainability and continuous improvement.[24] The model is used to raise awareness of 10 factors for sustainability, and prompt teams to consider actions to increase the likelihood of sustainability.[24] Application of this model demonstrated that while the SM raised awareness of determinants of sustainability and was perceived as valuable, teams found it difficult to understand and to apply the model routinely.[8,23] In particular, concerns were raised about the clarity the language used within the model, the user-friendliness of design, the length of time taken to complete the questions and suitability for continuous use in healthcare settings.[8]

The purpose of this study was to collaborate with stakeholders to develop a sustainability tool relevant to people in healthcare settings, and practical for use in improvement initiatives. In order to inform the tool development we explored the following research questions:

For peer review only - http://bmjopen.bmj.com/site/about/guidelines.xhtml

1. How do sustainability factors identified in the literature resonate with the experience of those in improvement projects in healthcare?
2. What features or characteristics make a sustainability tool valuable, practical and useful in real world healthcare practice?

Design

Setting: Research was conducted within the National Institute for Health Research (NIHR) Collaboration for Leadership in Applied Health Research and Care for Northwest London (CLAHRC NWL).[25] CLAHRC NWL improvement projects cover a range of health problems and disease areas that include primary care, secondary care, and community settings that are delivered over 18-24 months with the aim of sustaining improvements beyond this period. To support multidisciplinary teams to implement changes CLAHRC NWL systematically applies Quality Improvement (QI) methods such as the Model for Improvement and Action Effect Method.[19,23] The approach previously included use of the SM (2008-2013) but following internal evaluation and published research, it was acknowledged that a new more user-friendly method for sustainability was required to meet the needs of improvement teams.[8,23]

Participant Information: Participants in this study included members of CLAHRC NWL improvement initiative teams and staff. These members come from various backgrounds: multi-disciplinary healthcare practitioners (doctors, nurses, allied healthcare professionals), patients, carers, healthcare managers, directors, analysts and researchers (many participants hold overlapping roles ie. nurse who is also a project manager). Other participants were also included at the engagement event and piloting. Although the majority of attendance is from improvement teams, these event were are open to the public so additional participants included students, fellows, community members and industry partners. Specific participation from these groups in is outlined within each development stage and summarised in the results.

Methods: Tool development was conducted in 6 stages. The first 3 stages: scoping review, group discussions, and the stakeholder engagement event focussed on reviewing the literature findings and their resonance with stakeholders in this setting. The last 3 stages: interviews, small scale trialling and piloting contributed to designing and testing usability of the tool. The researchers within this study had participant observer roles.[26] They provided teaching, facilitation, and explanation throughout the development stages.

For peer review only - http://bmjopen.bmj.com/site/about/guidelines.xhtml

I. **Scoping Literature Review:** A scoping literature review was undertaken to examine the extent, range and nature of research activity related to sustainability approaches.[27] The *research question* guiding this review was: 'what approaches have been proposed to assess sustainability in healthcare and what sustainability factors are examined in each method'? *Identifying relevant studies:* A number of reviews had previously been published to identify factors for sustainability.[3,6,28] These reviews were used as a starting point to identify relevant authors and publications including snowballing of relevant journal articles, reference lists and the PubMed options of 'similar article' and 'cited by-' articles. *Selecting studies:* We sought approaches (published models, tools, strategies, and frameworks) that identified sustainability factors and themes. Papers that introduced or described a sustainability approach were included. Papers only defining or constructing concepts of sustainability outside of a structured approach were excluded. Commentary, posters, protocols, conference proceedings, editorials and perspectives were excluded. *Charting the data:* A data extraction form was developed for identified articles. Data extraction included: approach name, approach purpose, year published, type (model, scale, tool, checklist, framework), sustainability themes identified, and scoring mechanism. One author (LL) screened the retrieved papers for inclusion and extracted the data from the articles. Data extraction was independently checked against the full-text articles by a second author (CD). Any discrepancies were discussed between authors and were resolved by consensus. Inclusion and exclusion criteria were refined to reflect these discussions Agreement was reached for accuracy of all studies. *Summarizing the results:* All sustainability constructs (factors, questions, criteria etc.) identified in the approaches were extracted for thematic analysis. Aggregate themes were developed by combining similar or overlapping concepts and removing duplicate or redundant labels. Overarching sustainability themes were created using a mapping software.[29]

II. **Group discussions:** Three facilitated group discussions were held with CLAHRC NWL team members to understand the perceived relevance of the literature review results against CLAHRC NWL team expertise and experience. Discussions were held during a weekly CLAHRC NWL meeting between core staff. The themes from the scoping review were provided on paper hand-outs to the attendees and an open discussion took place to determine the resonance, and clarity of the themes presented. Observation notes were taken during group discussions. Notes were transcribed and findings were discussed

For peer review only - http://bmjopen.bmj.com/site/about/guidelines.xhtml

amongst the research team to inform iterations of language and representation of themes which were iteratively adapted and presented at consecutive discussions.

III. **Stakeholder engagement event:** Consolidated sustainability themes were presented to stakeholders at a CLAHRC NWL Collaborative Learning event in April 2014 to check the relevance and language against stakeholder views. In facilitated group discussions, participants provided their views on the resonance of these themes as well as identified any missing themes not seen in the literature. Designated note takers captured key learning and suggestions from the discussions. Field notes were collected and transcribed by one researcher. Findings were summarised and fed back to the research team to inform next steps and tool iteration.

IV. **Interviews:** Interviews aimed to collect in-depth information on value and practicality of tool design. A purposive sampling strategy was used to recruit interviewees. Participants were selected based on their role within diverse CLAHRC NWL improvement projects, their level of knowledge of their project, and their experience with the SM (we sought both those with and without experience in using the SM to ensure we had a balanced sample of those with prior opinions of the SM). This approach aimed to maximize the diversity of perspectives gained from the interviews.[30] All interviews were carried out face-to-face in a workplace setting by one author (LL). A semi-structured interview guide was used for all interviews. The interview guide used open ended questions on tool value and features that would be most or least desirable to identify interviewee priorities. Interview questions explored the design of questions and statements used to draw attention to factors for sustainability as well as views on collating and presenting data to facilitate discussion and action. No specific questions on the sustainability themes were asked as the themes and factors had undergone two iterations with participant comments so further in-depth study was deemed unnecessary. The final interview question showed participants an early mock-up of the tool design on which they commented freely. Interviews were audio recorded and uploaded onto qualitative software Nvivo (version 9). Audio recordings were coded directly on Nvivo using thematic content analysis.[31] A preliminary coding structure was developed using the interview questions as coding nodes, with themes inductively derived to summarise responses and record patterns in the data. The coding structure was iteratively developed, integrated and refined as further interviews were added to the dataset.[32] Results have been summarized using descriptive summaries and example quotes with explicit links to source text.

For peer review only - http://bmjopen.bmj.com/site/about/guidelines.xhtml

V. **Small scale trialling:** A group of individuals involved leading QI projects as part of a CLAHRC NWL fellowship programme were asked to trial a draft version of the tool. Trialling with this group aimed to understand the practical application of the tool including the approximate amount of time to complete by a wide range of people with diverse experience and expertise in improvement initiatives. Each participant filled out the tool for their own QI project. After completion, the group discussed the experience and posed questions on use. Critical feedback and suggestions for tool development were recorded as observation notes and summarised by the research team to inform tool iterations and piloting.

VI. **Piloting:** The resulting tool was piloted in July 2014. Piloting aimed to provide an opportunity for further comments and suggestions on practicality of the tool in healthcare settings, and to measure if the tool could be completed within an acceptable timeframe. A brief presentation given to participants to outline tool design and instructions for use. Participants were asked to fill out the tool for their individual QI projects. Individuals without a formal project were asked to fill out the tool with a hypothetical project in mind. Participants were given 15 minutes to complete the tool and a 20 minute facilitated group discussion followed. Designated note takers recorded key observations and feedback to inform tool iteration.

Results

Each development stage allowed for iterative adaptation and refinement of concepts, content, and design of the tool. Key iterations from each stage are summarized in Figure 1. The number and roles of participants is outlined in Table 1. The following section discusses results from each development stage and concludes with an introduction to the resultant tool.

Fig 1. Tool Development Stage and Iterations

Table 1: Number of Participants by Roles

	Number of Participants by Role							
**Development stage**	**Multi-disciplinary Healthcare Practitioner**	**Healthcare or Project Manager**	**Patient or Carer**	**Researcher or academic**	**Student/ Fellow**	**Data Analyst**	**Other**	**Total**
**I. Scoping Review**	-	-	-	-	-	-	-	0

For peer review only - http://bmjopen.bmj.com/site/about/guidelines.xhtml

**II. Group Discussions**	5	9	0	3	-	3	2	22
**III. Stakeholder Engagement Event**	22	12	10	8	7	1	7	74
**IV. Interviews**	6	3	1	-	-	-	2	12
**V. Small Scale testing**	6	1	3	1	11	-	-	11
**VI. Piloting**	30	16	17	6	8	-	20	106

I. **Scoping review:** The scoping review identified 81 publications for potential inclusion. Titles and abstracts were examined and 35 articles were retrieved in full text for full documentary analysis. Of these, 19 were excluded (16=no sustainability approach identified, 3=protocol, commentary or conference poster). In total 16 publications which identified sustainability approaches were included in this review. The sustainability approaches consisted of 6 models, 5 frameworks, 4 tools and 1 scale. The approaches aimed to evaluate sustainability, plan for sustainability or provided guidance to study or influence sustainability of initiatives. Thematic analysis identified 25 overarching themes impacting sustainability (Fig.1). Appendix A summarises the approaches found and the sustainability constructs extracted. Results demonstrated reasonable consensus in the literature on factors influencing sustainability. The review uncovered themes not explicitly covered in the SM such as considering *resources for the improvement*, and *the impact of the wider environment* on initiatives. No strategy explicitly included the importance of involving patients or carers as an aspect of sustainability which was an key finding from previous CLAHRC NWL work.[8]

II. **Group Discussions:** In total 22 individuals participated in the internal CLAHRC NWL group discussions. Discussions lead to combining themes that had different labels but were seen as having related or overlapping definitions. Discussions also identified where themes may be confusing and need to be expanded to underlying concepts to be relevant to improvement setting. For example, the literature theme of 'staff skills and capabilities' was expanded to include skills and capabilities of all those involved which may include as patients, carers or other stakeholders who participate in QI projects. Academic jargon and terms were also removed such as 'routinisation' which were seen as unhelpful or potentially confusing. These discussions

For peer review only

resulted in changes to the language used and theme consolidation to form a list of 12 factors impacting sustainability.(Fig 1)

III. **Stakeholder Engagement Event:** These factors were presented to stakeholders (n=74) in April 2014. The majority of the factors resonated well with stakeholders and were recognised as relevant to healthcare settings but in some cases the factor language needed to be adapted to align with stakeholder expertise and understanding. For example, the factor, 'Fit with Current Practice' was found to be problematic for participants. Although this factor was meant to convey the importance of interventions being aligned with current practice, many stakeholders mentioned that often improvements must be different from the current ways of working so trying to fit in with 'current practices' would not be desirable or possible. The factor was changed to 'Robust and Adaptable Processes' highlighting the need for interventions with the ability to adapt to local settings.

Stakeholders also identified missing concepts and concepts they felt were not clearly represented in the current factors. For example, *establishing a shared aim* for a project was suggested as an explicit prompt underlying the factor 'commitment to the improvement'. Desirable design elements were also highlighted. Participants stated that team members entering scores should have the opportunity to comment and suggest actions to improve the prospects for sustainability. They suggested that comments could be brought together for each factor to provide a starting point for action planning based on team member ideas. Suggested changes were used to adapt language and definitions as well as inform design of the scoring mechanism of the tool.

IV. **Interviews:** Interviews (n=12) allowed detailed views from diverse stakeholders to be identified. Interviewees represented perspectives from multiple QI projects. Projects included frailty assessment in acute care, patient experience measurement for sickle cell disease, clinical pathway development for allergic conditions in children, medication review in the elderly, bundle development for chronic obstructive pulmonary disease and diabetes education in community settings.

Interviewees discussed sustainability measurement, tool value and functionality. Stakeholders unanimously expressed a desire for a tool that is simple to use and quick to complete: "*Brevity I think is the theme. It is very hard to have yet another form to fill or another algorithm to think about, for people who are already over worked and over stretched*."(I3)

For peer review only - http://bmjopen.bmj.com/site/about/guidelines.xhtml

Interviewees desired a flexible tool with the option of quick review of the factors with any guidance or supportive text being brief and concise. Participants felt that using reflective statements to illicit an overall rating was a good way to get people thinking and provide an engaging format for the tool: "*I think overall impressions are powerful. You get a general feel and I think that is all you can hope for because otherwise…it will not be possible to make it user friendly" (I4)*

The data and outputs used to stimulate discussions needed to be simple to access, interpret, and present back to team members: *"I think most clinicians are familiar with a RAG (Red, Amber, Green) rating system so that would be easy for people to understand quickly." (I7)* From this feedback a draft tool was developed.

V. **Small Scale Trailing:** CLAHRC NWL fellows (n=11) trialled a draft version of the tool in June 2014. Each fellow was undertaking a QI project across diverse topic areas and settings (for example, service redesign, app development, patient experience measurement and staff training package development). Trialling the tool resulted in refinement the tool's prompt text to reduce the overall length. Stakeholders commented that the tool was a good reminder what to consider for sustainability but suggested changes to some of the language within the tool to remove terms perceived as 'jargon'. For example, in the factor 'Resources in place' original prompt text read: '*I am given sufficient **headspace** and **time** to dedicate to the improvement',* after discussion the term *'headspace'* was removed as it was seen as confusing to some participants. All participants completed the tool within 15 minutes. This timeframe was discussed and seen as acceptable, with the fellows commenting that no more than 15 minutes should be allotted for routine tool use in practice.

VI. **Piloting:** Piloting tool place with 106 participants (83 of which returned a completed tool to the research team). Fifty-two participants indicated that were involved in active QI projects. This included 9 CLAHRC NWL QI projects across diverse topics (such as sickle cell disease, allergic conditions in children, polypharmacy in the elderly, chronic obstructive pulmonary disease and congestive heart failure) as well as 19 projects outside of the CLAHRC NWL programme. Piloting with stakeholders demonstrated that majority of participants completed the tool in the projected 10-15 minute time period. Stakeholders engaged well with the prompts within the tool, commenting that they provided a simple format to begin consideration on how each factor may impact their initiatives. Participants commented that the tool was easy to use and that the

For peer review only - http://bmjopen.bmj.com/site/about/guidelines.xhtml

statements and questions enabled good discussion and 'promoted deeper thinking' allowing them to think about things they had not previously considered.

Regular scoring and review of factors was discussed and participants agreed in the necessity of consistently reviewing the changes to sustainability throughout their initiatives. Use every three months was recommended by stakeholders, as they felt this timeframe would be feasible given the ease and design of the tool and the potential for changes and turnover of staff in settings. Participants suggested the addition of a 'don't know' and 'no opinion' option to the tool as they did not want to make a forced choice and rate a factor that they did not have enough information to make an accurate rating. During piloting stakeholders questioned the appropriateness of the term 'sustainability'. Many stakeholders felt that 'sustainability' did not accurately capture the need for potential adaptation of initiatives or the desire to continually improve practice. Stakeholders wanted a term that would include both sustained improvements as well as the long term commitment to improvement. These discussions resulted in the term 'long term success' being used in place of sustainability to represent the aim that stakeholders desired. Feedback was used to iteratively develop the tool, which was then rolled out for wider use by CLAHRC NWL teams in January 2015. The final design of the tool and description for use is discussed below.

The Long Term Success Tool

Purpose: The Long Term Success Tool (LTST) aims to support those implementing improvements reflect on 12 key factors to identify risks and prompt actions to increase chances of sustainability over time.

The Factors: The factors included in the tool are: *Commitment to the improvement, Involvement, Skills and capabilities, Leadership, Team functioning, Resources in place, Evidence of benefits, Progress monitored for feedback and learning, Robust and adaptable processes, Alignment with organisational culture and priorities, Support for improvement, and Alignment with external political and financial environment.* The factors and their effects have been well documented in the literature[1,3,6,33] The presentation and language used to articulate the factors has been carefully developed and adapted with stakeholders to improve ease of understanding, and user-friendliness. The 12 factors have been organised within 3 emergent areas; People, Practice and Setting. Table 2 describes the factors and provides the statements for rating and supporting questions included within the tool.

For peer review only - http://bmjopen.bmj.com/site/about/guidelines.xhtml

Table 2: Long Term Success Factors: Purpose, Statement for rating and questions to consider

Factor	Purpose	Statement	Addition questions to support reflection
**People**			
**1. Commitment to the Improvement**	To reflect on both own personal commitment to the initiative and impression of commitment across the team as a whole to the initiative	*My team understands what the project is trying to achieve and believe this work will lead to improved processes and outcomes.*	Do you feel committed to the project? Do you understand what the project is trying to achieve?<br><br>Do you believe that this work will improve processes and outcomes? Do you believe there is reliable evidence (e.g. from literature, guidelines etc.) that the project will produce the desired benefits? Do you think there is commitment across the team as a whole?<br><br>Has a shared aim been established for your project?<br>If you think commitment is lacking, what do you think is the reason for this? What do you think should be done to address this?
**2. Involvement**	Reflect upon who has been involved and who may need to be engaged further for the initiative to achieve long term success. Asks about personal involvement and contribution and explores the involvement of patients, carers and members of the public who are impacted by the changes being made	*a) I have the opportunity to input into the project and I feel a sense of ownership towards the work. I am able to express my ideas freely which are openly considered by the team.*<br><br>*b) There is wide breadth of involvement from stakeholders including patients and members of the public who regularly feed into the project.*	a) Do you personally feel involved in the project? Are you given the opportunity to express your ideas and recommend changes to the project when necessary?<br><br>b) Do you think the project has involved the right people? Does your project involve patients affected by the improvement? Is there involvement from staff who will be delivering the improvement as part of their day-to day practice? Are the views of these groups taken on board?<br><br>Does the project have a good spread of views, skills and expertise? Are people with knowledge of mental as well as physical health involved? Do you believe involvement can be improved? Are there groups of people you still need to involve?
**3. Skills and capabilities**	Explores whether the staff and other people delivering the change have the skills to do so successfully and whether training of new members of the team has been planned for	*Staff have the necessary skills to deliver the improvement. Training and development opportunities are available to all staff, volunteers and other people involved.*	Do you feel able to fulfil your role within the project? Do you require further training or education? Do staff who will be delivering the improvement (e.g. frontline or support staff) have the skills to do so consistently and effectively?<br><br>Are new staff informed about the project and their role in it?<br><br>Do you think there are training needs associated with the improvement that should be addressed? What should be done to address to these needs?

For peer review only - http://bmjopen.bmj.com/site/about/guidelines.xhtml

**4. Leadership**	Asks if there is strong leadership in place and if the leaders are approachable, available and able to garner support for the initiative	*My project has supportive and respected leaders and/or champions who advocate for the improvement, communicate the vision, and effectively manage the process.*	Do you believe your project has strong leadership?<br>Are your project leaders actively involved in the project?<br>Are they able to garner support and enthusiasm for the work?<br>Are they available and approachable to members of the team if necessary?<br>Do the project leads effectively communicate the need for the change?<br>How do you think leadership could be strengthened?
**5. Team functioning:**	Explores the accountability and responsibilities for the workload involved in the initiative and ask if the team is working well together	*My project team is working well together. There are clear responsibilities for individuals and the work is shared across the team and does not rely on particular individuals.*	How well do you feel your project team is working together? Does the project team meet and communicate on a regular basis?<br><br>Have clear roles and responsibilities for project team members been established?<br>In your opinion, are team members fulfilling these roles and responsibilities?<br><br>Are skills and expertise of team members considered and put to use? What do you think can be done to improve team functioning?
**Practice**			
**6. Resources in place**	Explores if the necessary resources such as staff time, equipment and facilities have been dedicated to the initiative	*My project has financial support that will allow the improvement to achieve long term success. We have the necessary staff, material and equipment and I am given sufficient time to dedicate to the improvement.*	In your opinion, have enough resources been dedicated to support the project?<br><br>Do you believe the financial support provided will allow the improvement the project is trying to achieve to become part of normal working practice in the long term?<br><br>Does the project have enough staff to achieve the project aims? Do staff have enough time to spend on the improvement?<br><br>Are the materials needed (e.g. physical facilities, sites, equipment etc.) available to staff when they need them?<br><br>Are resources needed discussed by the team on a regular basis?<br>What resources do you think are lacking?
**7. Progress monitored for feedback and learning**	Encourages teams to consider what systems are in place to monitor the initiative overtime and how this information will be used to inform staff of further changes needed	*There is a monitoring system in place that allows the team to collect, manage and regularly review data. Feedback from the project is shared with me and other stakeholders on a regular basis.*	Have measures to enable continuous monitoring for your project been defined by the team?<br><br>Do you think these measures are able to assess the impact of the improvement? Can you suggest any changes to improve this?<br><br>Are these measures regularly assessed? Is this information used to make changes and improve project progress?<br>If the measures show lack of progress are the causes for this investigated?<br><br>Are project members and staff regularly informed about what is working well and what could be better?

For peer review only - http://bmjopen.bmj.com/site/about/guidelines.xhtml

**8. Evidence of benefits**	Asks if and how the benefits of the initiative are communicated to both staff and patients overtime	*There is evidence of benefits emerging from the project and this evidence is regularly communicated and visible to staff and patients.*	Does the evidence for your project include both the impact on physical and mental well-being of patients? Is there evidence (process and outcome measures) that the project is producing the desired impact on patients?<br><br>Is evidence of the projects' impact regularly shared with staff, patients and other stakeholders?<br>If evidence shows lack of progress, does the team investigate reasons for this?
**9. Robust and adaptable processes**	Reflects upon the need for initiatives to be adapted to local processes and emerging needs. It also asks about the process for recording successes and failures of changes made	*There is the opportunity to adapt the project to reflect local needs, setting and emerging evidence. Adaptations are documented and the successes and failures of changes are reported.*	Is there regular review of how the project is working?<br>How well does the project fit within current practices?<br><br>Do staff and team members need to adapt how they implement the improvement in response to challenges or changing care needs?<br><br>Does your team use PDSA cycles, Statistical Process Control and other quality improvement methods to test and document the changes made to the improvement?
**Setting**			
**10. Alignment with organisational culture and priorities**	Encourages teams to consider the need to align improvement initiatives to organisational strategies to gain executive buy-in and support as well as have the initiative become part of organisational policies and procedures	*The improvement my project is trying to achieve is aligned with the strategic aims and priorities of the organisation(s) we work within and our work contributes to these aims. Our work is supported by the policies and procedures within the organisation.*	Is the improvement your project is trying to achieve aligned with the organisational priorities?<br>Has this been promoted as something to help further the organisation's aims and priorities?<br><br>From your perspective, how well is the work of the project being integrated into the everyday operations of the organisation?<br><br>Does the project conflict with any other changes taking place within the organisation?<br>What could be done to better align your improvement to these priorities?
**11. Support for Improvement**	Explores the values and beliefs held within organisations related to continuous improvement and looks at the support given to staff and patients to be involved	*There are values and beliefs in my organisation(s) that emphasise the need to improve. Staff and management are supportive of improvement initiatives and continuous improvement is a priority for the organisation, staff and patients.*	Do you feel continuous improvement is a priority within your organisation?<br><br>Are staff and senior management receptive to improvement initiatives?<br><br>Are you supported by your leaders to participate in the improvement initiatives? Do senior leaders actively participate in improvement projects?

For peer review only - http://bmjopen.bmj.com/site/about/guidelines.xhtml

**12. Alignment with external political and financial environment**	Looks at the need for teams to be aware of the potential political and financial changes that may impact the initiative	*My project exists in a supportive economic and political environment. My team is aware of external pressures and incentives that may influence the project.*	Has your team considered the impact of the external environment on the project? For example are there economic pressures or political developments that may impact the project?<br><br>Is there political support for the implementation of your project?<br><br>Does your project help address external political or economic concerns? Does it contribute to the achievement of political objectives?<br><br>Are there plans to mitigate risks due to the external environment?

For peer review only

For peer review only - http://bmjopen.bmj.com/site/about/guidelines.xhtml

How it works:

The LTST is designed to create a platform for people to share their own views on sustainability as well as learn about the different views held within their team and to prompt discussion on any difference in opinion. To ensure teams are aware of how systems are evolving overtime, teams are encouraged to use the tool approximately every 3 months to assess progress and identify emerging risks continuously. Team members are asked to provide their overall impression of how their team is performing in each factor. Responses are collected on a paper questionnaire form or on the CLAHRC NWL Web Improvement System for Healthcare (WISH).[34] The full paper questionnaire can be found in Appendix B.

For each factor, team members are provided with a statement intended to prompt reflection. Supporting questions are available for each factor if team members would like more detail on what to potentially consider (Table 2). Team members score each factor individually and anonymously using a simple 5 point Likert scale (as well as no opinion and don't know options). Team members provide comments to suggest actions, explanations of their rating or concerns about progress against each factor.

Team scores are then brought together to produce aggregated outputs demonstrating how the initiative is performing against the given factors. Figure 2 shows an example a visual chart produced highlighting risks and differences in opinions. Table 3 shows an example of aggregated comments and actions highlighted within the tool.

Visual charts and comments are intended to facilitate discussion, bring differences of opinion or concerns into the open, and encourage actions to increase the chances of improvements being sustained. For CLAHRC NWL teams, time is allocated at progress meetings to review scores and plan actions.

Figure 2: Team level graph highlighting areas where the team is doing well, where more work is needed and differences of opinion.

Table 3: Comments and actions provided by team members during scoring which can be a starting point for discussion

Factor	Comments and Actions
Commitment to the Improvement	* Clear summary of project components and effects now in place from last time<br>* make sure all stakeholders attend meeting<br>* As a commissioner I didn't understand expectations and my role in the group - others seem very committed.<br>* Need to look at those engaging with the project
Involvement	
a)	* difficulties moving forward as until all stakeholders are engaged - unable to move forward

For peer review only

	* need to consider who is not involved and who would bring influence and value to the project
b)	* More patient/parent engagement at local level helpful<br>* more needed<br>* Patient and Public Involvement needs to be broadened<br>* No public / patient - don't feel it would be appropriate<br>* Patients/ Patient group and primary care practices poorly represented.
Skills and Capabilities of those involved	* of current clinical staff that I'm aware of<br>* capacity issues potentially can limit progress<br>* more nurse input<br>* not enough nursing staff employed to deliver project currently<br>* needs consultant/ general practitioner and nurse shadowing and specific training<br>* limited number of staff needs expansion

Discussion

The aim of this work was to develop a relevant and practical tool for sustainability that meets the needs of people in improvement initiatives. We explored how sustainability factors identified in the literature resonated with those in improvement projects and the features or characteristics which make a sustainability tool most valuable in real world healthcare practice. This work has shown that the majority of factors from the literature resonated well with stakeholders and were recognised as relevant to healthcare settings. In some cases, the literature findings needed to be adapted through changes to the language used to align with stakeholder preferences and understanding. Engaging stakeholder in the design process demonstrated that stakeholders valued clarity, conciseness, and simplicity for tool design with simple data interpretation and visual graphs. Receiving ongoing feedback during the development period from those who will use the tool has allowed us to design an approach that has responded to user needs and has addressed issues with language, length, and practicality along the way.

The LTST provides a mechanism for improvement teams to identify risks to sustainability and importantly can create an environment for team members to highlight specific actions to be taken and comment on ways to influence sustainability over time. The LTST builds on established literature and aligns well with other sustainability models and frameworks with all LTST factors reflected in one or more of the other approaches.[1,2,4,9,24,35–44] LTST is distinguished from other approaches by its practical design and ability to draw on team suggestions for action planning. Using participant ideas as a platform for action is a unique feature of the tool that is not present in other tools currently used in this area. Also unique to the LTST is that the allotted time for use, a identified barrier and challenge to other method use, has been explicitly tested and informed by end-users.[8,45] While many other methods involve either unknown or substantial time commitments, the LTST can be completed in approximately 10-15 minutes.[42,45]

For peer review only

There is also potential to supplement the use of other models or frameworks to complement the LTST. For example, if a project receives a low rating for the factor 'Robust and Adaptable Processes', The Model for Highly Adoptable Improvement toolkit may be used to aid the team in further understanding of where the intervention can be adapted.[46]

Limitations: A limitation of this work is the use of a snowballing scoping review opposed to a systematic review. Conducting a full systematic review may have uncovered further articles and/or approaches but due to the practical time constraints of our programme this was not possible. The results of our review have fed into a protocol for a full systematic review on available sustainability approaches which is now underway.[47] The results of this review will inform future adaptation of the LTST.

Another limitation of this work is the potential for responder bias throughout development stages. Prior relationships between researchers and participants was identified as a possible source of bias, namely, social desirability bias, as participants may have responded in ways that were seen as more desirable to the researchers.[48] Another source of possible responder bias stems from the sustainability themes and factors being presented to participants during development stages which may have directed participant responses and reaction. Although participants were given the opportunity to provide their views on the resonance of these themes as well as identify alternative themes, participants may have been more likely to agree with presented findings which may have impacted our findings. As the development of the tool was centred on user preferences, attempts were made to communicate and reiterate there were no 'right' answer to questions. We also attempted to mitigate this effect by having multiple stages for feedback, with diverse facilitators and a wide variety of participants. We also had a researcher unknown to the majority of the interviewees conduct the interviews.

Another possible limitation is related to the generalizability of the tool to teams with little or no QI experience. Although the tool was developed by people with significant QI experience, the tool is intended to be used by those with all levels of QI knowledge. The process of involving those would use the tool in the design and piloting of the tool sought to ensure the tool could be used by all people involved in an improvement project. Tailoring of the tool language and the instructions were done to ensure people with little QI experience or knowledge would be able to use the tool. Further observation and study of the application of the tool is needed to assess if application is impacted by this design.

For peer review only

Future Research: While attempts have been made to respond to user preferences and create a practical tool, further research is required to assess tool effectiveness and engagement over time. A 3-year programme of research with teams at CLAHRC NWL and other groups internationally is currently underway to investigate tool impact on initiative processes and practices and examine actions taken by improvement teams to sustain improvements across diverse settings and environments. This longitudinal study will also investigate tool links to sustainability outcomes to assess what impact tool use may have on sustained QI projects. In order to facilitate and study the use of the tool by those outside of Northwest London, the tool is freely available along with a structured excel spreadsheet for data entry to produce automated graphs and charts (Table 4).

Table 4: Information on using the Long Term Success Tool

Using the Long Term Success Tool in Your Setting
The Long Term Success tool has been designed on the CLAHRC NWL WISH system. For those who do not have access to this system, the Long Term Success Tool questionnaire form and Excel spreadsheet can be downloaded with this paper. The tool can be used along with Table 2 which provides supporting questions to describe the potential items to consider within each factor. The tool can be used by individuals and teams. Responses can be input into the Excel spreadsheet which enables users to produce similar graphs and outputs to ones shown in this paper. The spreadsheet enables 8 possible entry points for a team (up to 20 team members) and will aggregate team data overtime for review and action planning. (Appendices B and C)

Conclusion

The development of the LTST has reinforced the importance of working with stakeholders to design strategies which respond to their needs and preferences and can practicality be implemented in real-world settings. This study provides valuable information on the process of developing a new approach to sustainability that is both conceptually rigorous and practical for use with healthcare improvement teams.

Acknowledgments: The authors would like to thank Dr Alan Poots for his contribution to designing and developing the Long Term Success Tool spreadsheets. We would also like to thank the stakeholders involved throughout the development process.

Competing Interests: None

Author Contribution: LL, CD, JR and DB were involved in conceiving the study. LL conducted the interviews. LL, CD, JR and DB contributed to data collection in the group discussions and piloting. CD and LL conducted the analysis. LL drafted the initial manuscript. CD, JR and DB contributed to revisions and the final draft of the manuscript.

For peer review only

Funding: This work was funded by the National Institute for Health Research in the Collaboration for Leadership in Applied Health Research and Care for Northwest London (CLAHRC) programme.

Disclaimer: This article presents independent research commissioned by the National Institute for Health Research (NIHR) under the Collaborations for Leadership in Applied Health Research and Care (CLAHRC) programme for North West London. The views expressed in this publication are those of the author(s) and not necessarily those of the NHS, the NIHR or the Department of Health.

Ethics statement: Ethics approval was not required for this work as it was part of a service evaluation and improvement project. All interviewees provided verbal consent for the recording of the interviews and were informed that all data would be anonymised for publication.

Data sharing statement: No further data is available.

For peer review only

References

1 Shediac-Rizkallah MC, Bone LR. Planning for the sustainability of community-based health programs: conceptual frameworks and future directions for research, practice and policy. *Health Educ Res* 1998;**13**:87–108.

2 Sirkin HL, Keenan P, Jackson A. The hard side of change mangement. *Harv Bus Rev* 2005;:109–18.

3 Stirman SW, Kimberly J, Cook N, *et al.* The sustainability of new programs and innovations : a review of the empirical literature and recommendations for future research. *Implement Sci* 2012;**7**:17. doi:10.1186/1748-5908-7-17

4 Bowman CC, Sobo EJ, Asch SM, *et al.* Measuring persistence of implementation: QUERI Series. *Implement Sci* 2008;**3**. doi:10.1186/1748-5908-3-21

5 Williams L, Daggett V, Slaven JE, *et al.* A cluster-randomised quality improvement study to improve two inpatient stroke quality indicators. *BMJ Qual Saf* 2015;:bmjqs-2015-004188. doi:10.1136/bmjqs-2015-004188

6 Scheirer MA. Is Sustainability Possible? A Review and Commentary on Empirical Studies of Program Sustainability. *Am J Eval* 2005;**26**:320–47. doi:10.1177/1098214005278752

7 Martin GP, Weaver S, Currie G, *et al.* Innovation sustainability in challenging health-care contexts: embedding clinically led change in routine practice. *Heal Serv Manag Res* 2012;**25**. doi:10.1177/0951484812474246

8 Doyle C, Howe C, Woodcock T, *et al.* Making change last: applying the NHS institute for innovation and improvement sustainability model to healthcare improvement. *Implement Sci* 2013;**8**:127. doi:10.1186/1748-5908-8-127

9 Feldstein AC, Glasgow RE. A Practical , Robust Implementation for Integrating Research Findings into Practice. *Jt Comm J Qual patient Saf* 2008;**34**:228–43.

10 Chambers D a, Glasgow RE, Stange KC. The dynamic sustainability framework: addressing the paradox of sustainment amid ongoing change. *Implement Sci* 2013;**8**:117. doi:10.1186/1748-5908-8-117

11 Moullin JC, Sabater-hernández D, Fernandez-llimos F, *et al.* A systematic review of

implementation frameworks of innovations in healthcare and resulting generic implementation framework. *Heal Res Policy Syst* 2015;**13**:1–11. doi:10.1186/s12961-015-0005-z

12 Scheirer MA, Dearing JW. An agenda for research on the sustainability of public health programs. *Am J Public Health* 2011;**101**:2059–67. doi:10.2105/AJPH.2011.300193

13 Wiek A, Ness B, Schweizer-Ries P, *et al.* From complex systems analysis to transformational change: A comparative appraisal of sustainability science projects. *Sustain Sci* 2012;**7**:5–24. doi:10.1007/s11625-011-0148-y

14 Grol RPTM, Bosch MC, Hulscher MEJL, *et al.* Planning and Studying Improvement in Patient Care: The Use of Theoretical Perspectives. *Milbank Q* 2007;**85**:93–138.

15 Ullrich PM, Sahay A, Stetler CB. Use of implementation theory: A focus on PARIHS. *Worldviews Evidence-Based Nurs* 2014;**11**:26–34. doi:10.1111/wvn.12016

16 Drew S, Judge A, May C, *et al.* Implementation of secondary fracture prevention services after hip fracture: a qualitative study using extended Normalization Process Theory. *Implement Sci* 2015;**10**:57.

17 Leon N, Lewin S, Mathews C, *et al.* Implementing a provider-initiated testing and counselling (PITC) intervention in Cape town, South Africa: a process evaluation using the normalisation process model. *Implement Sci* 2013;**8**:97.

18 Chilundo BGM, Cliff JL, Mariano ARE, *et al.* Relaunch of the official community health worker programme in Mozambique : is there a sustainable basis for iCCM policy ? 2015;:54–64. doi:10.1093/heapol/czv036

19 Reed JE, McNicholas C, Woodcock T, *et al.* Designing quality improvement initiatives: the action effect method, a structured approach to identifying and articulating programme theory. *BMJ Qual Saf* 2014;:1–9.

20 Reed JE, Card AJ. The problem with Plan-Do-Study-Act cycles. *BMJ Qual Saf* 2015;:1–6. doi:10.1136/bmjqs-2015-005076

21 Waring JJ, Bishop S. Lean healthcare: Rhetoric, ritual and resistance. *Soc Sci Med* 2010;**71**:1332–40. doi:10.1016/j.socscimed.2010.06.028

22 Taylor MJ, McNicholas C, Nicolay C, *et al.* Systematic review of the application of the plan-do-study-act method to improve quality in healthcare. *BMJ Qual Saf* 2014;**23**:290–8. doi:10.1136/bmjqs-2013-001862

23 Howe C, Randall K, Chalkley S, *et al.* Supporting improvement in a quality collaborative. *Br J Healthc Manag* 2013;**19**.

24 Maher L, Gustafson D, Evans A. *Sustainability Model and guide*. Coventry, UK: : NHS Institute for Innovation and Improvement 2005.

25 Caldwell S, Mays N. Studying policy implementation using a macro, meso and micro frame analysis: the case of the Collaboration for Leadership in Applied Health Research & Care (CLAHRC) programme nationally and in North West London. *Heal Res Policy Syst* 2012;**10**. doi:doi:10.1186/1478-4505-10-32

26 Gold R. Roles in sociological field observation. *Soc Forces* 1958;**36**:213–7.

27 Arksey H, O'Malley L. Scoping studies: towards a methodological framework. *Int J Soc*

For peer review only

Res Methodol 2004;**8**:19–32.

28 Buchanan D, Fitzgerald L, Ketley D, *et al.* No going back: A review of the literature on sustaining organizational change. *Int J Manag Rev* 2005;**7**:189–205. doi:10.1111/j.1468-2370.2005.00111.x

29 Ltd Xm. XMind. 2016.

30 Onwuegbuzie AJ, Leech NL. Sampling Designs in Qualitative Research : Making the Sampling Process More Public. *Qual Rep* 2007;**12**:19–20. doi:10.1007/s11135-006-9000-3

31 Wainwright M, Russell A. Using NVivo Audio-Coding: Practical, Sensorial and Epistemological Considerations. *Soc Res Updat* 2010;:1–4. doi:10.1177/147332501000900210002

32 Elo S, Kyngäs H. The qualitative content analysis process. *J Adv Nurs* 2008;**62**:107–15. doi:10.1111/j.1365-2648.2007.04569.x

33 NHS Modernisation Agency. Complexity of sustaining healthcare improvements: what have we learned so far : Research into Practice report 13. 2004.

34 Curcin V, Woodcock T, Poots AJ, *et al.* Model-driven approach to data collection and reporting for quality improvement. *J Biomed Inform* 2014;**52**:151–62.

35 Goodman RM, McLeroy KR, Steckler AB, *et al.* Development of level of institutionalization scales for health promotion programs. *Health Educ Q* 1993;**20**:161–78.

36 Dale BG, Boaden RJ, Wilcox M, *et al.* Total quality management sustaining audit tool: Description and use. *Total Qual Manag* 1997;**8**:395–408. doi:10.1080/0954412979406

37 Glasgow RE, Vogt TM, Boles SM. Evaluating the public health impact of health promotion interventions: the RE-AIM framework. *Am J Public Health* 1999;**89**:1322–7.

38 Johnson K, Hays C, Center H, *et al.* Building capacity and sustainable prevention innovations: a sustainability planning model. *Eval Program Plann* 2004;**27**:135–49. doi:10.1016/j.evalprogplan.2004.01.002

39 Gruen RL, Elliott JH, Nolan ML, *et al.* Sustainability science: an integrated approach for health-programme planning. *Lancet* 2008;**372**:1579–89. doi:10.1016/S0140-6736(08)61659-1

40 Luke D a, Calhoun A, Robichaux CB, *et al.* The Program Sustainability Assessment Tool: a new instrument for public health programs. *Prev Chronic Dis* 2014;**11**:130184. doi:10.5888/pcd11.130184

41 Kaplan HC, Provost LP, Froehle CM, *et al.* The Model for Understanding Success in Quality (MUSIQ): building a theory of context in healthcare quality improvement. *BMJ Qual Saf* 2012;**21**:13–20.

42 Johnson K, Fisher D, Wandersman A, *et al.* A Sustainability Toolkit for Prevention: Using getting to Outcomes. 2012. doi:10.1073/pnas.0703993104

43 Kotter P. Leading change : why transformation efforts fail. *Harv Bus Rev* 1995;:59–67.

44 Damschroder LJ, Aron DC, Keith RE, *et al.* Fostering implementation of health services

For peer review only

research findings into practice: a consolidated framework for advancing implementation science. *Implement Sci* 2009;**4**:50. doi:10.1186/1748-5908-4-50

45 Hooker L, Small R, Humphreys C, *et al.* Applying normalization process theory to understand implementation of a family violence screening and care model in maternal and child health nursing practice: a mixed method process evaluation of a randomised controlled trial. *Implement Sci* 2015;**10**:39.

46 Hayes CW, Batalden PB, Goldmann D. A 'work smarter, not harder' approach to improving healthcare quality. *BMJ Qual Saf* 2014;**24**:100–2. doi:10.1136/bmjqs-2014-003673

47 Lennox L, Maher L, Bell D, *et al.* The sustainability landscape: a systematic review of sustainability strategies and their impact in healthcare settings. *PROSPERO Int Prospect Regist Syst Rev* 2016;**CRD4201604**:1–4.

48 Kalton G, Schuman H. The Effect of the Question on Survey Responses: A Review. *J R Stat Soc* 1982;**145**:42–73.

Figure Legend:

Figure	Page
**Figure 1.** Tool Development Stage and Iterations	9
**Figure 2:** Team level graph highlighting areas where the team is doing well, where more work is needed and differences of opinion	18

For peer review only

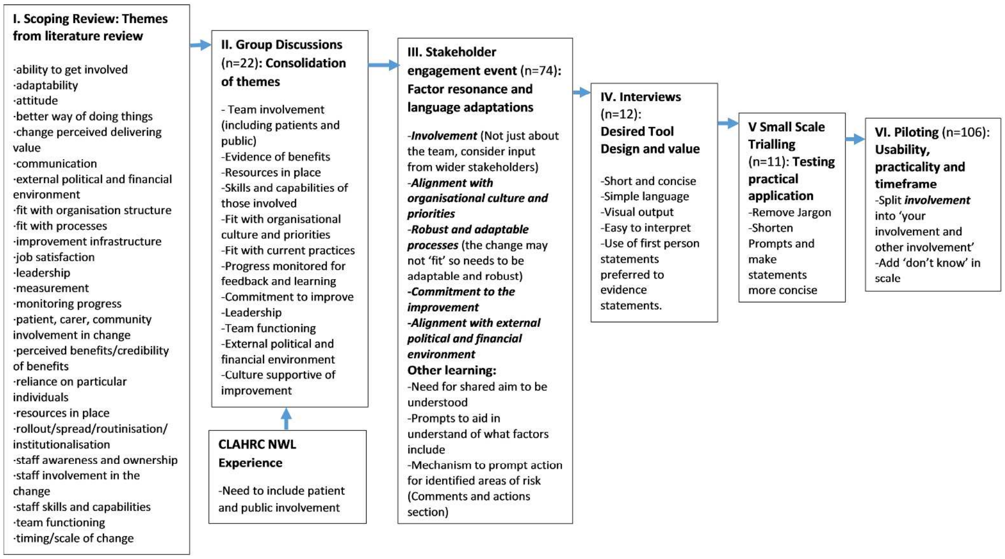

Fig 1

261x144mm (300 x 300 DPI)

review only

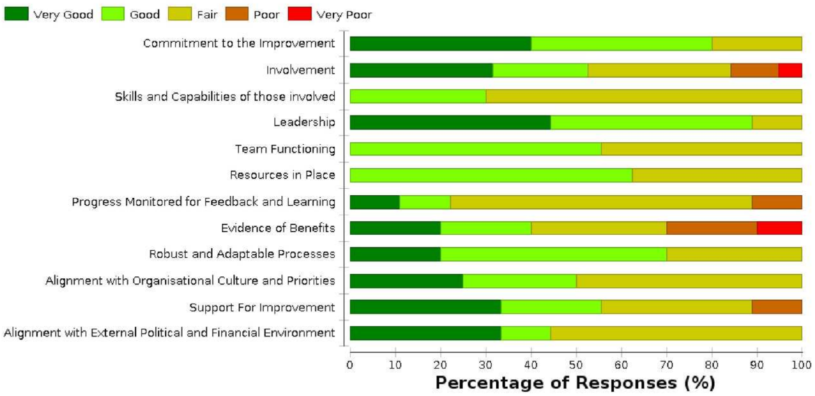

Fig. 2

297x210mm (300 x 300 DPI)

Appendix A

Authors	Year	Model/Concept	Purpose	Type	Sustainability Constructs Identified	Scoring
Goodman	1993	Level of institutionalization (LOIN) scales	Research instrument and diagnostic tool	Scale	8 factor model- 15 three part items. Production routine, Production niche saturation, Maintenance routine, Maintenance niche saturation, supportive routine, Supportive niche saturation, Managerial routine, Managerial niche saturation	Likert scale
Kotter	1995	Anchoring Change	Raise awareness of the common errors made during change initiatives	Model	Establishing a sense of urgency, Formation of a powerful guiding coalition, Creating a vision, Communicating the vision, Empowering others to act on the vision, Planning for and creating short term wins, Consolidating improvements and producing more change, Institutionalising new approaches.	none
Dale et al.	1997	The TQM sustainability audit tool (TQMSAT)	Looks for a specific set of predetermined negative factors; that have been identified as detrimental to sustaining TQM	Tool	Internal/external environment, Management Style, Policies, Organisation structure, Process of change. 23 sub themes including: communication, human resource, improvement infrastructure, education and training	Rate each of the issues on a scale of 1 to 6
Shediac-Rizkallah& Bone	1998	Conceptual framework for planning for sustainability of community based health programs	An organizing framework for conceptualizing and measuring sustainability and tentative guidelines to facilitate sustainability in community programs	Conceptual Framework	(1) Project design and implementation factors, Project negotiation process. Project effectiveness. Project duration. Project financing Project type, Training<br>(2) Factors within the organizational setting: Institutional strength Integration with existing programs/services Program champion/leadership<br>(3) Factors in the broader community environment: Socioeconomic and political considerations Community participation.	none
Glasgow et al.	1999	RE-AIM	Provides a framework for determining what programs are worth sustained investment and for identifying those that work in real-world environments	Framework	Reach- patient and staff participation, Efficacy- positive and negative effects of interventions, Adoption-proportion and representation of settings, Implementation-extent to which intervention is delivered as intended and Maintenance-institutionalisation, routine, culture and norms.	0 to 1 (or 0% to 100%) scale.

Appendix A

Johnson et al	2004	A sustainability Planning Model	Addresses factors known to inhibit efforts to sustain an innovation	Model	Sustainability Capacity, Administrative structures and linkages, Champion roles and leadership actions, resources, policies and procedures, Expertise, Sustainable innovation attributes, Alignment, positive relationships, implementation quality and effectiveness, sustainable actions	none
Sirkin	2005	DICE tool	To rate and track transformation initiative progress	Tool	Duration, Integrity of performance, Senior management commitment, Local-level commitment, effort	Likert scale (1-4) Low scores being better.
Edwards	2007	Catholic Healthcare partners HF-GAP Sustainability Assessment (AHRQ)	To trigger planning for sustainability early in a project's design	Framework and checklist	Five components for developing sustainability: identity goals, infrastructure, incentives, incremental opportunities for participation, and integration	Rate your organisation: -1: Problem, 0: Neither positive or negative, 1: Strength
Gruen	2008	Health-programme sustainability	To provide comprehensive sustainability planning	Model	Context and resource availability, encompassing health concerns, interventions, and drivers, emphasising dynamic interactions between components	none
Feldstein & Glasgow	2008	A practical robust implementation and sustainability model (PRISM)	To enhance implementation and sustainability	Model	Program, external environment, Implementation and Sustainability Infrastructure, Recipients with 39 sub-elements to assess	none
Bowman	2008	QUERI Implementation Model	Proposes a number of recommendations regarding the design of a sustainability analysis	Framework and model	1) intervention fit, 2) intervention fidelity, 3) intervention dose, and<br>4) level of the intervention target	none
Johnson et al	2009	A sustainability toolkit for prevention using getting to outcomes	To provide a process to organize, implement, and evaluate an effective plan for sustaining prevention infrastructure and interventions	Toolkit	Capacity, Infrastructure, Intervention, Routinization, Benefits to diverse populations	none

Appendix A

Damschroder	2009	Consolidated Framework For Implementation Research	To guide formative evaluations and build the implementation knowledge base across multiple studies and settings	Framework	8 constructs related to the intervention (e.g., evidence strength and quality), 4 constructs to outer setting (e.g., patient needs and resources), 12 constructs to inner setting (e.g., culture, leadership engagement), 5 constructs related to individual characteristics, and 8 constructs related to process (e.g., plan, evaluate, and reflect)	none
Maher	2010	NHS sustainability Model	To predict the likelihood of sustainability and guide teams to things they could do to increase the chances that the change for improvement will be sustained	Model	10 factors: Staff involvement and training, Staff attitudes towards sustaining the change, Senior leadership engagement, Clinical leadership, Fit with the organisation's strategic aims and culture, Infrastructure for sustainability, Benefits beyond helping patient, Credibility of the benefits, Adaptability of improved process, Effectiveness of the system to monitor progress	Weighted scoring system- Max 30 points
Douglas	2012	The Program Sustainability Assessment Tool	To assess and plan for sustainability risks and develop an action plans	Tool	Political support, funding stability, partnerships organisational capacity, program evaluation, program adaptation, communications, strategic planning	7 point Likert scale (To little or no extent- To a very great extent)
Kaplan	2012	The Model for Understanding Success in Quality (MUSIQ)	To examine the role of context in QI and how context influences QI success	Model	Factors effecting context: External Environment, Organisation, QI support and Capacity, Microsystem, QI Team, Miscellaneous	none

For peer review only - http://bmjopen.bmj.com/site/about/guidelines.xhtml

Long Term Success Tool

This tool aims to aid you in planning for long term success of your work. You will be asked to rate 12 factors that have been identified to impact long term success from current literature and evidence.

Each rating should represent an overall impression of how you believe your project is doing. Please use the boxes to highlight any comments or actions needed to address the factors.

A. Please specify which project you are completing the form for:

B. Please rate the project in the following factors:

1. Commitment to the improvement

My team understands what the project is trying to achieve and believe this work will lead to improved processes and outcomes.

❍ Very Good ❍ Good ❍ Fair ❍ Poor ❍ Very Poor ❍ No Opinion ❍ Don't Know

Comments and actions:

2. Involvement

a) I have the opportunity to input into the project and I feel a sense of ownership towards the work. I am able to express my ideas freely which are openly considered by the team.

❍ Very Good ❍ Good ❍ Fair ❍ Poor ❍ Very Poor ❍ No Opinion ❍ Don't Know

Comments and actions:

b) There is wide breadth of involvement from stakeholders including patients and members of the public who regularly feed into the project.

❍ Very Good ❍ Good ❍ Fair ❍ Poor ❍ Very Poor ❍ No Opinion ❍ Don't Know

Comments and actions:

3. Skills and capabilities

Staff have the necessary skills to deliver the improvement. Training and development opportunities are available to all staff, volunteers and other people involved.

❍ Very Good ❍ Good ❍ Fair ❍ Poor ❍ Very Poor ❍ No Opinion ❍ Don't Know

Comments and actions:

4. Leadership

My project has supportive and respected leaders and/or champions who advocate for the improvement, communicate the vision, and effectively manage the process.

❍ Very Good ❍ Good ❍ Fair ❍ Poor ❍ Very Poor ❍ No Opinion ❍ Don't Know

Comments and actions:

5. Team functioning

My project team is working well together. There are clear responsibilities for individuals and the work is shared across the team and does not rely on particular individuals.

❍ Very Good ❍ Good ❍ Fair ❍ Poor ❍ Very Poor ❍ No Opinion ❍ Don't Know

Comments and actions:

Please turn over

©NIHR CLAHRC
Northwest London

For peer review only

Long Term Success Tool

6. Resources in place

My project has financial support that will allow the improvement to achieve long term success. We have the necessary staff, material and equipment. I am given sufficient time to dedicate to the improvement.

❍ Very Good ❍ Good ❍ Fair ❍ Poor ❍ Very Poor ❍ No Opinion ❍ Don't Know

Comments and actions:

7. Progress monitored for feedback and learning

There is a monitoring system in place that allows the team to collect, manage and regularly review data. Feedback from the project is shared with me and other stakeholders on a regular basis.

❍ Very Good ❍ Good ❍ Fair ❍ Poor ❍ Very Poor ❍ No Opinion ❍ Don't Know

Comments and actions:

8. Evidence of benefits

There is evidence of benefits emerging from the project and this evidence is regularly communicated and visible to staff and patients.

❍ Very Good ❍ Good ❍ Fair ❍ Poor ❍ Very Poor ❍ No Opinion ❍ Don't Know

Comments and actions:

9. Robust and adaptable processes

There is the opportunity to adapt the project to reflect local needs, setting and emerging evidence. Adaptations are documented and the successes and failures of changes are reported.

❍ Very Good ❍ Good ❍ Fair ❍ Poor ❍ Very Poor ❍ No Opinion ❍ Don't Know

Comments and actions:

10. Alignment with organisational culture and priorities

The improvement my project is trying to achieve is aligned with the strategic aims and priorities of the organisation(s) we work within and our work contributes to these aims. Our work is supported by the policies and procedures within the organisation.

❍ Very Good ❍ Good ❍ Fair ❍ Poor ❍ Very Poor ❍ No Opinion ❍ Don't Know

Comments and actions:

11. Support for improvement

There are values and beliefs in my organisation(s) that emphasise the need to improve. Staff and management are supportive of improvement initiatives and continuous improvement is a priority for the organisation, staff and patients.

❍ Very Good ❍ Good ❍ Fair ❍ Poor ❍ Very Poor ❍ No Opinion ❍ Don't Know

Comments and actions:

12. Alignment with the political and financial environment

My project exists in a supportive economic and political environment. My team is aware of external pressures and incentives that may influence the project.

❍ Very Good ❍ Good ❍ Fair ❍ Poor ❍ Very Poor ❍ No Opinion ❍ Don't Know

Comments and actions:

Thank you for your time

©NIHR CLAHRC
Northwest London

For peer review only

The Long Term Success Tool- Excel spreadsheet for Data input

Instructions for Use

This tool aims to help you ensure the improvements you are attempting to make with your project deliver lasting benefits to patients.

This tool is designed to encourage reflection on 12 factors identified in the literature as important to long term success and to identify what action your team can take. The tool provides a simple way to rate your impressions on these factors (e.g. to give an overall impression of how your project is doing in relation to each of these factors) and suggest action that is likely to increase the prospects of your work having lasting benefits. Although some factors may feel more relevant at different stages in the project, there is value in considering all of these factors at an early stage.

Your rating may be influenced by how you feel about a particular factor (e.g. do you personally feel committed to this project?) and/or your overall impression of how your team views a factor (e.g. do you get the impression there is commitment across the team?). A comment box is provided for you to explain concerns you may have and to suggest actions for your team.

Your ratings, comments and suggested actions will be combined with those of other team members and an overall graphs representing the team's views will be produced. These outputs can be brought to designated meetings where the team can discuss results and actions that should be taken to improve specific factors.

The tool will take approximately 15 minutes to complete.

How to:

1. Every team member should use the Long Term Success Paper form to score and comment on each factor. The Long Term Success Questions within the framework can be used to support team members in scoring the factors.
2. Team scores should be entered in the Excel spreadsheet for each time point.
3. The scores are aggreagted in included in the automated table and visual charts.
4. Outputs can be used to begin a team conversation on risks and possible next steps to enhance the chances of making lasting improvements.
5. The spreadsheet enables 8 possible entry points for a team (up to 20 team members) and will aggregate team data overtime for review and action planning.

For peer review only

	factor	Commitment to the Improvement	Involvement (A)	Involvement (B)	Skills and Capabilities of those involved	Leadership	Team Functioning	Resources in Place	Progress Monitored for Feedback and Learning	Evidence of Benefits	Robust and Adaptable Processes	Alignment with Organisational Culture and Priorities	Support for Improvement	Alignment with External Political and Financial Environment
person	1	very good	very good	good	very good	very good	very good	good	very good	good	don't know	no opinion	good	good
	2	poor	very poor	fair	fair	fair	poor	poor	don't know	no opinion	good	very good	very good	fair
	3	good	fair	very good	good	poor	fair	good	poor	no opinion	don't know	good	fair	good
	4													
	5													
	6													
	7													
	8													
	9													
	10													
	11													
	12													
	13													
	14													
	15													
	16													
	17													
	18													
	19													
	20													

Comments	Commitment to the Improvement	Involvement (A)	Involvement (B)	Skills and Capabilities of those involved	Leadership	Team Functioning	Resources in Place	Progress Monitored for Feedback and Learning	Evidence of Benefits	Robust and Adaptable Processes	Alignment with Organisational Culture and Priorities	Support for Improvement	Alignment with External Political and Financial Environment
1													
2													
3													
4													
5													
6													
7													
8													
9													
10													
11													
12													
13													
14													
15													
16													
17													
18													
19													
20													

For peer review only - http://bmjopen.bmj.com/site/about/guidelines.xhtml

factor	Commitment to the Improvement	Involvement (A)	Involvement (B)	Skills and Capabilities of those involved	Leadership	Team Functioning	Resources in Place	Progress Monitored for Feedback and Learning	Evidence of Benefits	Robust and Adaptable Processes	Alignment with Organisational Culture and Priorities	Support for Improvement	Alignment with External Political and Financial Environment
very good	1	1	1	1	1	1	0	1	0	0	1	1	0
good	1	0	1	1	0	0	2	0	1	1	1	1	2
fair	0	1	1	1	1	1	0	0	0	0	0	1	1
poor	1	0	0	0	1	1	1	1	0	0	0	0	0
very poor	0	1	0	0	0	0	0	0	0	0	0	0	0
total respons	3	3	3	3	3	3	3	2	1	1	2	3	3
don't know	0	0	0	0	0	0	0	1	0	2	0	0	0
no opinion	0	0	0	0	0	0	0	0	2	0	1	0	0
not able to ra	0	0	0	0	0	0	0	1	2	2	1	0	0
Total answers	3	3	3	3	3	3	3	3	3	3	3	3	3
max	very good	very good	very good	very good	very good	very good	good	very good	good	good	very good	very good	good
min	poor	very poor	fair	fair	poor	poor	poor	poor	good	good	good	fair	fair
MEDIAN	good	fair	good	good	fair	fair	good	G/F	good	good	VG/G	good	good

For peer review only - http://bmjopen.bmj.com/site/about/guidelines.xhtml

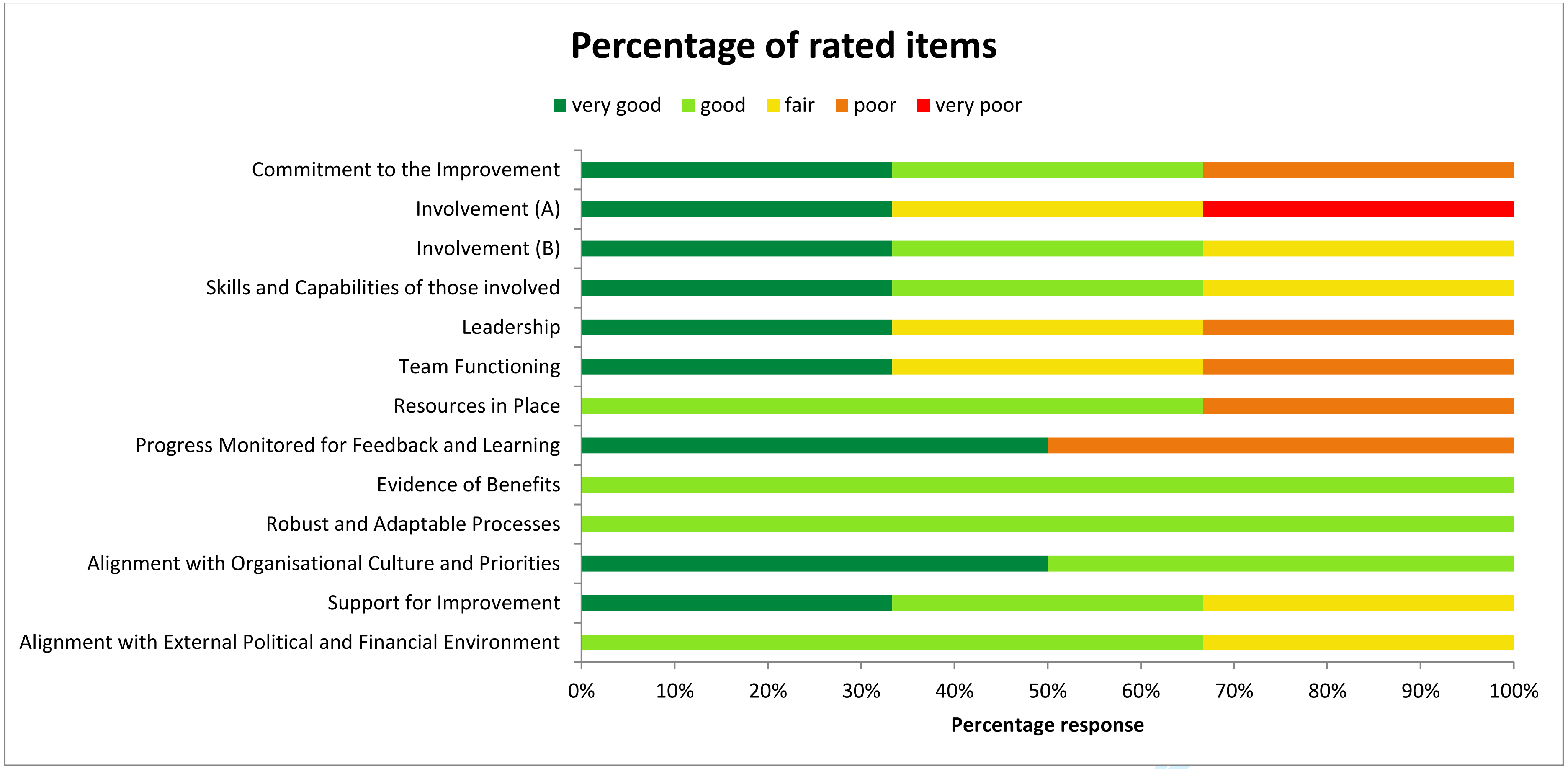
Percentage of rated items
very good
good
fair
poor
very poor
Commitment to the Improvement
Involvement (A)
Involvement (B)
Skills and Capabilities of those involved
Leadership
Team Functioning
Resources in Place
Progress Monitored for Feedback and Learning
Evidence of Benefits
Robust and Adaptable Processes
Alignment with Organisational Culture and Priorities
Support for Improvement
Alignment with External Political and Financial Environment
0%
10%
20%
30%
40%
50%
60%
70%
80%
90%
100%
Percentage response

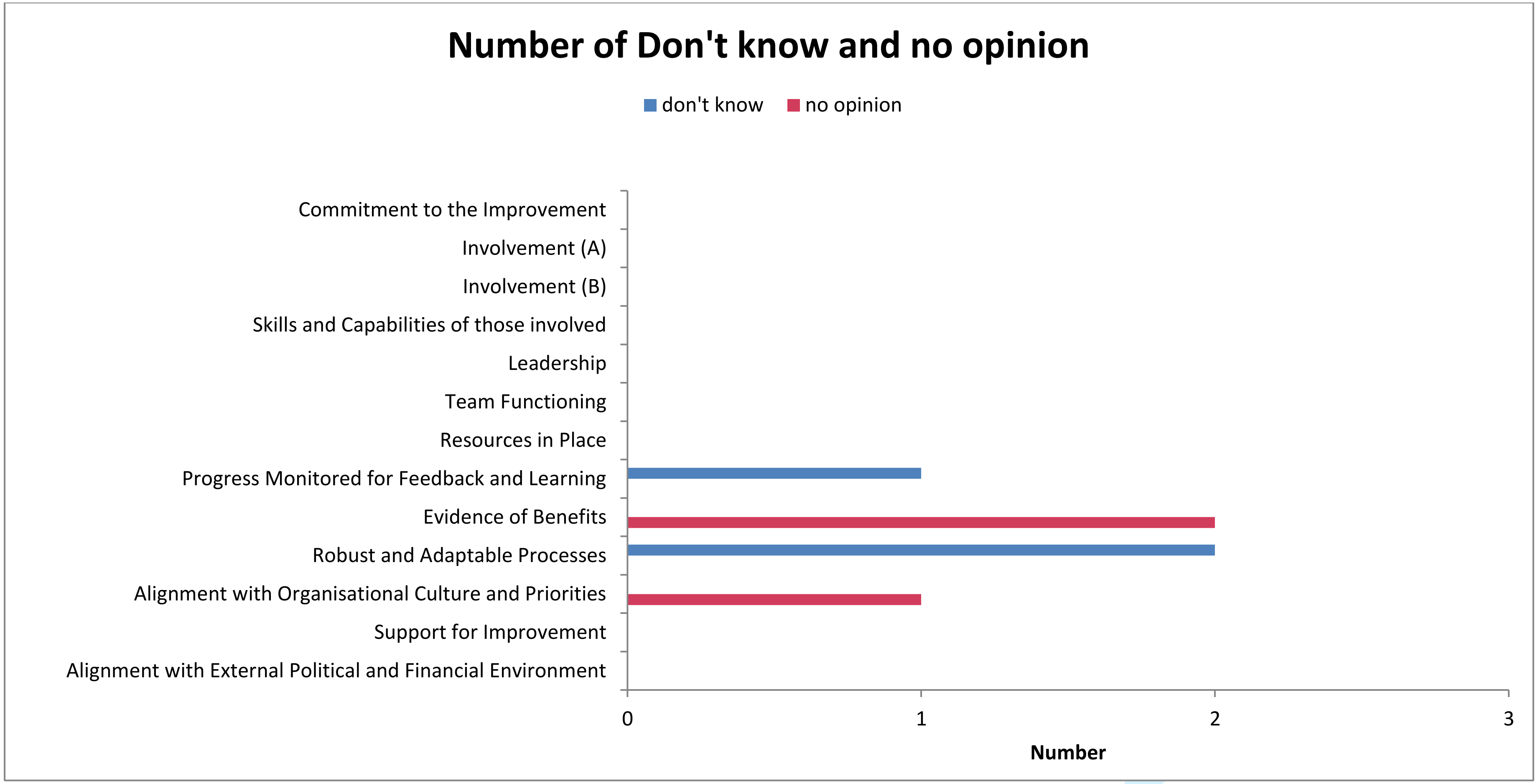
Number of Don't know and no opinion
don't know
no opinion
Commitment to the Improvement
Involvement (A)
Involvement (B)
Skills and Capabilities of those involved
Leadership
Team Functioning
Resources in Place
Progress Monitored for Feedback and Learning
Evidence of Benefits
Robust and Adaptable Processes
Alignment with Organisational Culture and Priorities
Support for Improvement
Alignment with External Political and Financial Environment
0
1
2
3
Number

Consolidated criteria for reporting qualitative studies (COREQ): 32-item checklist

**No Item Guide questions/description**	**Response**
Personal Characteristics	
1. Interviewer/facilitator: Which author/s conducted the interview or focus group?	Pg. 8
2. Credentials: What were the researcher's credentials? E.g. PhD, MD	RN, MSc PH, PhD Student. Not reported in paper.
3. Occupation: What was their occupation at the time of the study?	Research Assistant, Not reported in paper.
4. Gender: Was the researcher male or female?	Female. Not reported in paper.
5. Experience and training: What experience or training did the researcher have?	4 years research experience, conducting qualitative research. Not reported in paper.
**Relationship with participants**	
6. Relationship established: Was a relationship established prior to study commencement?	The researchers were known to and had prior relationships with the participants in the CLAHRC NWL discussion groups. The researcher conducting the interviews had no prior relationship with any of the interviewees besides 2 QI facilitators who worked with teams but were employed within CLAHRC NWL. Page 20
7. Participant knowledge of the interviewer: What did the participants know about the researcher? e.g. personal goals, reasons for doing the research	Various knowledge based on previous professional encounters. No personal knowledge. Not reported in paper.
8. Interviewer characteristics: What characteristics were reported about the interviewer/facilitator? e.g. Bias, assumptions, reasons and interests in the research topic	PhD student interested in sustainability of improvements. Previous experience of working in healthcare as a registered nursing. Not reported in paper.
**Domain 2: study design**	
Theoretical framework	
9. Methodological orientation and Theory: What methodological orientation was stated to underpin the study? e.g. grounded theory, discourse analysis, ethnography, phenomenology, content analysis	This study was underpinned with thematic content analysis. Pg 8
Participant selection	

For peer review only

10. Sampling: How were participants selected? e.g. purposive, convenience, consecutive, snowball	Purposive Pg 8
11. Method of approach How were participants approached? e.g. face-to-face, telephone, mail, email	Face-to-face Pg. 8
12. Sample size: How many participants were in the study?	Pg. 9<br>CLAHRC NWL Group Discussions: (n=22) Pg.<br>Stakeholder engagement event (74)<br>Interviews (n=12)<br>Small scale trialling (n=11)<br>Piloting (n=106)
13. Non-participation How many people refused to participate or dropped out? Reasons?	For interviews, No participants refused. One participant accepted but was unable to meet with in the interview timeline so was replaced with a colleague. Not reported in paper.<br>For the other group stages (discussion event, testing and piloting) participation was voluntary so no formal refusals were noted.
Setting	
14. Setting of data collection Where was the data collected? e.g. home, clinic, workplace	Workplace or public space for engagement event and piloting. Pg. 8
15. Presence of non-participants Was anyone else present besides the participants and researchers?	No. Not reported in paper.
16. Description of sample What are the important characteristics of the sample? e.g. demographic data, date	CLAHRC NWL team improvement team members and CLAHRC NWL Staff members Pg. 6
Data collection	
17. Interview guide Were questions, prompts, guides provided by the authors? Was it pilot tested?	Yes. Pg 8.
18. Repeat interviews Were repeat interviews carried out? If yes, how many?	none
19. Audio/visual recording Did the research use audio or visual recording to collect the data?	Audio recorded p.8
20. Field notes Were field notes made during and/or after the interview or focus group?	Yes. Pg. 7-9.
21. Duration What was the duration of the interviews or focus group?	30 -60 minutes Not reported in paper.
22. Data saturation Was data saturation discussed?	Saturation was not the goal of the interviews. Not reported in paper.

For peer review only

23. Transcripts returned Were transcripts returned to participants for comment and/or correction?	No.
**Domain 3: analysis and findings**	
Data analysis	
24. Number of data coders How many data coders coded the data?	1 coder. Main themes and coding labels discussed with co-authors and amended as needed. Not reported in paper
25. Description of the coding tree Did authors provide a description of the coding tree?	Not reported in paper.
26. Derivation of themes Were themes identified in advance or derived from the data?	Deductively derived from the data with content analysis with a predefined coding structure to respond to interview questions. Pg. 8
27. Software What software, if applicable, was used to manage the data?	Nvivo software was used pg. 8.
28. Participant checking: Did participants provide feedback on the findings?	Participants were able to comment on the findings when given the chance to test and pilot the tool. Pg. 11-12.
Reporting	
29. Quotations presented Were participant quotations presented to illustrate the themes / findings? Was each quotation identified? e.g. participant number	Yes pg. 10.
30. Data and findings consistent: Was there consistency between the data presented and the findings?	Yes pg. 9-12.
31. Clarity of major themes Were major themes clearly presented in the findings?	Yes pg. 10
32. Clarity of minor themes Is there a description of diverse cases or discussion of minor themes?	Not reported in paper

For peer review only
